# Supplementary material for: Best-BRA (Is subpectoral or prepectoral implant placement best in immediate breast reconstruction?): a protocol for a pilot randomised controlled trial of subpectoral versus prepectoral immediate implant-based breast reconstruction in women following mastectomy
Source: BMJ Open. 2021 Nov 30;11(11):e050886. doi: 10.1136/bmjopen-2021-050886 (PMC8634330; doi:10.1136/bmjopen-2021-050886)
Supplement: Supplementary data [file bmjopen-2021-050886supp001.pdf]

Best-BRA Study
(Is subpectoral or pre-pectoral implant placement **Best** in immediate **BR**e**A**st reconstruction?)

Patient Information Leaflet

We are inviting you to take part in a research study

- We would like to invite you to take part in a research study. Before you decide to take part, we need you to understand why the research is being done and what it will involve for you.
- Please take time to read the following information carefully and talk to others if you wish.
- Please ask questions using the contact details if anything you read is not clear or you would like further information.

Important things that you need to know

- We are inviting women who are having a mastectomy with an immediate implant-based breast reconstruction to take part in a research study called the Best-BRA study.
- We want to find out whether placing the implant underneath the chest muscle (subpectoral) or above the muscle (pre-pectoral) is best for women who are suitable for either procedure.
- Both procedures are standard practice commonly performed across the UK; neither are new or experimental.
- In the Best-BRA Study, we want to initially find out if such a study is possible before we then do a much larger definitive study.
- Women suitable for both procedures who take part will be allocated to one of two groups: subpectoral or pre-pectoral implant-based breast reconstruction
- We will look at women's satisfaction with their implant-based reconstruction by asking them to complete three study questionnaires for up to 12 months.
- Women who take part can leave the study at any time and will have no impact on the care they receive.
- In this research study we will use information from you and your medical records. We will only use information that we need for the research study.
- We will let very few people know your name or contact details, and only if they really need it for this study.

Roberts K, *et al. BMJ Open* 2021; 11:e050886. doi: 10.1136/bmjopen-2021-050886

- Everyone involved in this study will keep your data safe and secure. We will also follow all privacy rules.
- At the end of the study we will save some of the clinical data in case we need to check it and for future research.
- We will make sure no-one can work out who you are from the reports we write.
- ***If you would like more information on what is involved, please turn over and read Section A***

Contact details:
Name: <INSERT Name of Local Research Nurse>,
Address: <Insert Local hospital address>
Tel: <INSERT Local hospital phone number>
Email: bestbra-study@bristol.ac.uk

BMJ Publishing Group Limited (BMJ) disclaims all liability and responsibility arising from any reliance placed on this supplemental material which has been supplied by the author(s)

University of BRISTOL

North Bristol NHS Trust

PART A: Why is this study being done and what will happen if you take part?

What is the purpose of the study?

Breast cancer affects one in eight women in the UK and 40% of all women with breast cancer have a mastectomy (removal of the breast) as their surgical treatment. Losing a breast may affect women's self-esteem and body image and breast reconstruction is offered to improve their quality of life.

Breast reconstruction using implants is the most commonly performed procedure in the UK. There are two ways in which implants can be placed during implant-based breast reconstruction; the implants can be placed under the chest muscle (a subpectoral implant) or placed on top of the chest muscle (pre-pectoral implant). Both are standard procedures commonly performed across the UK.

In the Best-BRA study, we want to find out if surgeons and women would be willing to take part in a study to find out if putting the implant on top of the muscle or underneath it improves women's satisfaction with the outcome of their reconstruction. There is currently no good evidence which implant procedure is best for patients (see below).

**Subpectoral reconstruction**	**Pre-pectoral reconstruction**
Implant placed <u>under</u> the chest (pectoral) muscle, implant may or may not be partially covered in mesh	Implant placed <u>on top</u> of the chest (pectoral) muscle, implant usually covered in mesh
Will involve 1-2 nights in hospital	May involve 1 night in hospital or you may be allowed home on the same day
Your surgeon is likely to place 1 or more drains at the time of surgery	Your surgeon is likely to place 1 or more drains at the time of surgery
The implant may move when the chest muscle contracts (this is called animation)	As the implant is on top of the muscle it does not move as much when the chest wall muscle contracts
The muscle covers the implant so the edge of the implant and any ripples on the surface are less easy to see.	The edges of the implant and ripples on the implant surface may be easier to see
May require further surgery to improve the appearance of the reconstruction over time	May be require further surgery to the reconstruction (such as fat transfer) to hide the implant over time

We also want to capture how information about the study is conveyed to patients. This will help us understand how research studies are explained to people and if there are any improvements we can make.

Why have I been invited?

Your surgeon is working with the research team to understand more about which implant-based technique is best. You have been asked to take part in this study because you are having a mastectomy and are suitable for an immediate implant-based breast reconstruction.

We hope to recruit women having immediate implant-based breast reconstruction from UK hospitals.

Do I have to take part?

No. It is your choice whether or not to take part in the Best-BRA study. If you decide to not take part, the treatment and care you receive from your doctors will not be affected in any way. If you do decide to take part, you are also free to leave the study at any time you wish, without giving a reason.

What is involved in the study?

If you take part in the Best-BRA study, following your mastectomy, you will have an immediate implant-based breast reconstruction where either the implant will be placed on top of the chest (pre-pectoral) muscle or below it (subpectoral), both of which are standard procedures. As we do not know which is best for women, the type of surgery you will have will be allocated through a process called

University of BRISTOL | independent cancer patients' voice www.independentcancerpatientsvoice.org.uk | FUNDED BY NIHR National Institute for Health Research | BRISTOL TRIALS CENTRE

Roberts K, *et al. BMJ Open* 2021; 11:e050886. doi: 10.1136/bmjopen-2021-050886

randomisation. This means you will have an equal chance of having a pre-pectoral implant-based breast reconstruction procedure or a subpectoral implant-based breast reconstruction procedure. Randomisation is used as it creates groups of patients that are similar except for whether the implant is placed under or on top of the muscle. This will enable a fair comparison of the different techniques so we can assess at the end of the study which technique is best. Neither you, the surgeons, or the research team can choose which group you go in, as this could result in the groups being unequal and the findings unreliable. It is important that you only agree to take part if you are prepared to accept either of the two implant-based breast reconstruction procedures.

With your consent, we plan to record conversations where the Best-BRA study is discussed with you. We would like to see how the study is explained to you and if we can improve this. This is optional. As part of this, we may also invite some women to have an optional discussion (research 'interview') with a researcher to explore their views on the study and whether they chose to take part or not. This may take up to an hour, it will be over the telephone and will be recorded with consent.

If I decide to take part, what happens next?

The diagram on page 5 illustrates the flow of the study, which we detail here;

A) First discussion about the Best-BRA study

You will have an appointment with your surgeon and breast care nurse to discuss your surgery and the study in more detail. Following this discussion, a member of the local research team will meet with you at one of your routine clinical appointments, or telephone you, to discuss the study in more detail, answer any questions you may have and arrange your first study visit at your local hospital (see below).

With your consent we would like to record this discussion and any future discussions you have about the study, regardless of whether you decide to take part in the main study or not.

B) First study appointment at your local hospital

This appointment will be at your local hospital if it coincides with one of your routine appointments, otherwise it will be carried out over the phone or using video conferencing if you are happy to do this. At this appointment, a member of the local research team will confirm that you are still happy to take part.

You will be asked to:

- **Sign 2 consent forms** confirming that you agree to the recording of discussions (unless agreed earlier) and that you agree to take part in the study. The consent forms can be completed online or over the phone and they can be posted to you if you are not at the hospital;
- **Complete a study questionnaire** about your general health, how you feel about your body, your experiences and your quality of life before the surgery. The questionnaire should take no more than 20 minutes to complete.
- **Provide some additional information** for the purposes of the study, e.g. contact details, GP information, date of birth.
- If you are having **pre and post-operative photographs** taken as part of your care, you will be asked at this appointment if you are happy for the surgeon or breast care nurse to share these with the research team and they will be reviewed as part of the study. This is optional.

You will then be informed whether you will be in the "Pre-pectoral implant group" or the "Subpectoral implant Group".

We will inform your GP of your participation in the study and let them know which group you have been allocated to.

Roberts K, *et al. BMJ Open* 2021; 11:e050886. doi: 10.1136/bmjopen-2021-050886

University of BRISTOL

North Bristol NHS Trust

Your first consultation to discuss treatment options
Study will be introduced briefly*

- Read Patient Information Leaflet
- Ask any questions

A) First discussion about the Best-BRA study if you decide to have implant-based reconstruction*

At routine clinical appointment or via telephone

Research nurse will arrange your first study appointment

B) First study appointment remotely or at your local hospital*

- Discuss study and ask questions
- Sign consent form
- Request to contact for optional research interview
- Complete study questionnaires
- Allocation to study group

**Pre-pectoral** implant-based breast reconstruction	**OR**	**Subpectoral** implant-based breast reconstruction

C) Surgery at your local hospital

- Preparation for surgery by clinical team- this will include a pre-operative assessment clinic
- You will receive your allocated implant-based breast reconstruction surgery
- Recovery after surgery and clinical follow-up
- Pain Assessment 24 hours and 1 week

D) 3 month questionnaire at home

- Complete study questionnaire 3 months after your first study visit, this can be done via post or online

E) 12 month questionnaire at home

- Complete another study questionnaire 12 months after your first study visit, this can be done via post or online

* These consultations with your consent will be audio recorded

Roberts K, *et al. BMJ Open* 2021; 11:e050886. doi: 10.1136/bmjopen-2021-050886

C) The day of your surgery

You will be invited to attend your local hospital to **have your surgery** (a mastectomy followed by either pre-pectoral or subpectoral implant-based breast reconstruction). The clinical team will prepare you for your surgery as they would during normal NHS care. A member of the research team will also record some other information from your hospital notes about your planned surgery.

The surgeons who will carry out your breast reconstruction in this study perform them regularly. You will receive the usual NHS treatment and aftercare at your hospital (e.g. medical tests, procedures, and follow-up appointments). If you have any doubts, concerns or questions about your operation you should talk to your consultant surgeon, before agreeing to the surgery or to take part in the research.

Please note: you may need to attend a pre-operative assessment clinic before your surgery. Your clinical team will advise you on local procedures.

24 hours and 1 week following your surgery we will ask you about any pain you are experiencing following the surgery. This will be a single question asking you to record this using a scale which can be completed online or with help from the research team.

D) Questionnaire to complete at home: 3 months after your first study visit

You will be asked to **complete a study questionnaire** about your general health, how you feel about your body, your experiences and your quality of life after the surgery. The questionnaire should take no longer than 20 minutes to complete and can be done by post (we will provide a pre-paid envelope) or online.

E) Questionnaire to complete at home: 12 months after your first study visit

12 months after your implant-based breast reconstruction, you will be asked to **complete another study questionnaire** about your general health, how you feel about your body, your experiences and your quality of life after the surgery.

What are the possible benefits of taking part?

This study will not benefit you directly but the information that you provide will help to improve the future management of patients who undergo implant breast reconstructive surgery. Also, some people enjoy being part of a research study because of the close contact with research staff and their opportunity to share their opinions and experiences of their condition and treatments.

What are the possible disadvantages and risks of taking part?

There should be no additional risks to routine NHS practice of either implant-based reconstruction procedure, and neither are new or experimental. You will have the same risks as anyone having immediate implant-based reconstruction. Your doctor will explain the risks and benefit of each procedure, and they will provide relevant hospital leaflets. The questionnaires will take approximately 20 minutes of your time.

What happens after the study is over?

After completing your 12 months questionnaire, you will return to standard NHS care. However, we would like to be able to contact you after this, with your permission. We would like to contact you again to a) check on your long-term health, for example by sending you other questionnaires to add information to what we already know about you, or by checking NHS medical records; and b) to ask you if you would like to take part in other relevant studies. You will not have to reply to any questionnaires or take part in other studies unless you want to at that time.

What if I change my mind and I don't want to carry on with the study?

Roberts K, *et al. BMJ Open* 2021; 11:e050886. doi: 10.1136/bmjopen-2021-050886

You are free to withdraw from the study at any point without giving reason. Your medical treatment will not be affected. If you wish to leave the study, please speak to your doctor or research nurse. If you decide you no longer wish to take part, any information we have collected up until the point you leave will be retained and used in the analysis of the trial results.

What about expenses and travel?

We will provide you with prepaid envelopes to return the study questionnaires if you opt to not complete them online or by telephone. We can offer to cover some travel expenses for study specific hospital visit(s).

What should I do now?

After reading this information sheet, we hope that you will be interested in taking part. You can contact the research nurse if you have any questions. If you would like to take part please also read section B of this information sheet.

PART B: Further general information about the study and what will happen to your data if you decide to take part

Will the information I provide be kept confidential?

Yes. We will follow ethical and legal practice and all information about you will be handled in confidence.

Any information that is collected about you during the study will be kept strictly confidential. All information collected for the study at any time will be stored using a 'study identity number' for confidentiality and will be kept secure using passwords on a University of Bristol server. The information will be handled in line with data protection requirements and will only be available to those responsible for maintaining research standards. We will need to use information from you and/or from your medical records for this research project. This information will include your:

• Initials

• NHS number

• Name

• Gender

• Date of birth

• Contact details (for example: postcode, telephone number, email address)

Your data will be stored and used in compliance with the relevant, current data protection laws; Data Protection Act 2018 and General Data Protection Regulation (GDPR). North Bristol NHS Trust is the sponsor for this study based in the United Kingdom. We will be using information from you and/or your medical records in order to undertake this study and North Bristol NHS Trust and the Bristol Trials Centre (BTC, University of Bristol) will act as joint data controllers for this study. This means that we are responsible for looking after your information safely and using it properly. The BTC at the University of Bristol, who manage the study on behalf of North Bristol NHS Trust, will keep identifiable information about you for 5 years after the study has finished, which is considered good practice for clinical trials. After that period, the data will be securely destroyed (except audio-recordings which will be retained for future studies with your consent).

Your rights to access, change or move your information are limited, as we need to manage your information in specific ways in order for the research to be reliable and accurate. If you withdraw from the study, we will keep the information about you that we have already obtained. To safeguard your rights, we will use the minimum personally identifiable information possible.

Other researchers may wish to access anonymised data from this study in the future. If you take part in the Best-BRA study, anonymous data collected in this study may be used in future studies with the necessary approval. This will not include names,

addresses or dates of birth, and it will not be possible to identify you.

If you consent to recording of discussions with study staff and/or a research interview, then all recorded data will be handled in the strictest confidence. Data will be captured on an encrypted audio-recorder or using the video conferencing facilities, transferred securely to the Best-BRA study team at the University of Bristol, and held on a password protected computer. If video conferencing is used, only the audio part of the recording will be retained. Files will be labelled with a reference number (as opposed to your name or any personal information). Recordings will be transcribed (i.e. written word for word) by University of Bristol employees or their authorised representatives. Transcripts (i.e. a written account of what was said) will be anonymised so that you cannot be identified. Data will be analysed and used for training, teaching, research and publication purposes in Best-BRA and future studies. Any quotes used will be anonymised and extracts of recordings will be voice-modified so that you cannot be identified.

Anonymised transcripts of recordings may be made available to other researchers outside of the Best-BRA study via controlled access if they secure the necessary approvals. Data from the anonymised transcripts may be used for purposes not related to this study, but it will not be possible to identify you from them.

You may change your mind about having these consultations or the research interview recorded at any time without it affecting your legal rights or medical care, although any recordings collected before your withdrawal may be retained and used anonymously in a way that will not identify you. Agreeing to record your consultations or taking part in a research interview does not commit you to taking part in the Best-BRA research study.

You can find out more about how we use your information at:

- www.hra.nhs.uk/information-about-patients/
- Our leaflet available from www.nbt.nhs.uk/PatientResearchdata
- By asking one of the research team at bestbra-study@bristol.ac.uk.
- By contacting Helen Williamson (Head of Information Governance) at helen.e.williamson@nbt.nhs.uk or by ringing 0117 41 44767.

Your hospital will collect information from you and/or your medical records for this research study in accordance with our instructions.

Your hospital will use your name, NHS number and contact details to contact you about the research study, and make sure that relevant information about the study is recorded for your care, and to oversee the quality of the study. Individuals from North Bristol NHS Trust, the BTC and regulatory organisations may look at your medical and research records to check the accuracy of the research study. Your hospital will pass these details to the BTC along with the information collected from you and/or your medical records. The only people in North Bristol NHS Trust and the BTC who will have access to information that identifies you will be people who need to contact you to audit the data collection process. The people who analyse the information will not be able to identify you and will not be able to find out your name, NHS number or contact details.

Your hospital will keep identifiable information about you from this study for 5 years after the study has finished (this does not include the audio-recordings).

What will happen to the results of the research study?

The results will be published in medical journals, presented at conferences with other healthcare professionals and specialists, reported on open access databases and open platform research registries and made publicly available. A short version will also be available to you. No one will be able to identify you from any of the study reports.

Who is organising or sponsoring the research?

The study is being funded by the National Institute of Health Research (NIHR) (reference 2016-16-019) The research is being carried out by a group of experienced doctors and researchers at each of the hospitals involved in the study and the University of Bristol. This study is sponsored by North Bristol NHS Trust, and the Bristol Trials Centre are responsible for managing the study. The study is being conducted by Miss Shelley Potter, Consultant Senior Lecturer at the Centre for Surgical Research University of Bristol.

Who has reviewed the study?

This study has been reviewed by the Health Research Authority and NHS Research Ethics Committee <INSERT Name of REC Committee> who have provided approval for this study to be conducted in the NHS.

What if I have any concerns?

If you have a concern regarding your care as a patient, please discuss this with your surgeon or nurse. If you become unable or unwilling to continue taking part in the Best-BRA Study, we would withdraw you from it. Your medical treatment will continue as usual with your hospital team and GP.

In the unlikely event that something does go wrong, and you are harmed during the research study there are no special compensation arrangements. If you are harmed and this is due to someone's negligence, then you may have grounds for a legal action for compensation against North Bristol NHS Trust, but you may have to pay your legal costs. The normal National Health Service complaints mechanisms will still be available to you.

If you have any questions about the study, or any aspect of your treatment or health whilst on the study, please ask to speak to your Best-BRA study research nurse or surgeon <INSERT name, address, email address, telephone numbers including the 24 hour emergency contact number>.

Alternatively, you can contact the Trial Manager (email: bestbra-study@bristol.ac.uk; phone: 0117 9287351).

The Patient Advice and Liaison Service (PALS) /Advice & Complaints Team (ACT) [delete as appropriate] should be contacted for any complaints. Your local PALS/ACT [delete as appropriate] is:

<INSERT local details>

THANK YOU FOR TAKING THE TIME TO READ THIS INFORMATION.
PLEASE KEEP A COPY FOR YOUR RECORDS.

Funding Acknowledgement: *This project is funded by the National Institute for Health Research (NIHR) (project reference CS-2016-16-019). The views expressed are those of the author(s) and not necessarily those of the NIHR or the Department of health and Social Care.*

BTC Acknowledgement: *This study was designed and delivered in collaboration with the Bristol Trials Centre (BTC), a UKCRC registered clinical trials unit which, as part of the Bristol Trials Centre, is in receipt of National Institute for Health Research CTU support funding.*

Roberts K, *et al. BMJ Open* 2021; 11:e050886. doi: 10.1136/bmjopen-2021-050886
