# Supplementary material for: Best-BRA (Is subpectoral or prepectoral implant placement best in immediate breast reconstruction?): a protocol for a pilot randomised controlled trial of subpectoral versus prepectoral immediate implant-based breast reconstruction in women following mastectomy
Source: BMJ Open. 2021 Nov 30;11(11):e050886. doi: 10.1136/bmjopen-2021-050886 (PMC8634330; doi:10.1136/bmjopen-2021-050886)
Supplement: Supplementary data [file bmjopen-2021-050886supp002.pdf]

Best-BRA Study

(Is subpectoral or pre-pectoral implant placement **Best** in immediate **BR**e**A**st reconstruction?)

Participant Consent Form

Participant ID:						

Please initial Relevant boxes

1. I confirm that I have read the patient Information leaflet <INSERT version & date> for the above study. I have had the opportunity to consider the information, ask questions and have had these answered satisfactorily.

2. I understand that my participation is voluntary and that I am free to withdraw at any time without giving any reason, without my medical care or legal rights being affected. I understand that all information collected up until the point I withdraw will be retained for analysis.

3. I understand that after the study ends, the research data (results) collected may be made "open data". This means the data will be publicly available and may be used for purposes not related to this study. However, any personal information that could identify me will be removed or changed before files are shared with other researchers or results are made public. This does not apply to QRI data that is 'controlled access' (see separate consent form regarding the audio-recording of consultations).

4. I understand that relevant sections of my medical notes and data collected during the study, including personal identifiable data may be looked at by individuals from regulatory authorities, the University of Bristol study research team or from the NHS Trust, to ensure that the research is conducted appropriately. I give permission for these individuals to have access to my records.

5. I agree that my GP will be told that I am taking part in this research study.

6. I give consent for the data collected in this trial to be used in future ethically approved studies on the understanding that all information will continue to be kept securely and remain confidential.

Please continue on next page

Roberts K, *et al. BMJ Open* 2021; 11:e050886. doi: 10.1136/bmjopen-2021-050886

7. *OPTIONAL: I agree for my pre-operative and post-operative photographs if they have been taken, to be shared securely with the research team and to be analysed as part of the study* ☐

8. *OPTIONAL: I agree to being contacted in the future by the research team to provide information about my long-term health status. I understand I can then decide to not take part in any future studies if I change my mind.* ☐

7. I agree to take part in the above Best-BRA Study. ☐

____________________________	________________________	________________________
**Name of patient**	**Signature**	**Date**
____________________________	________________________	________________________
**Name of person taking consent**<br>You must have signed the<br>Site Signature & Delegation Log	**Signature**	**Date**

Original to be kept in the Investigator Site File, 1 copy in hospital notes, 1 copy to the patient, 1 copy to the Trial Office

Funding Acknowledgement: *This project is funded by the National Institute for Health Research (NIHR) HTA programme (project reference 17/95/03). The views expressed are those of the author(s) and not necessarily those of the NIHR or the Department of health and Social Care.*

BRTC Acknowledgement: *This study was designed and delivered in collaboration with the Bristol Randomised Trials Collaboration (BRTC), a UKCRC registered clinical trials unit which, as part of the Bristol Trials Centre, is in receipt of National Institute for Health Research CTU support funding.*

Roberts K, *et al. BMJ Open* 2021; 11:e050886. doi: 10.1136/bmjopen-2021-050886
