# Supplementary material for: Best-BRA (Is subpectoral or prepectoral implant placement best in immediate breast reconstruction?): a protocol for a pilot randomised controlled trial of subpectoral versus prepectoral immediate implant-based breast reconstruction in women following mastectomy
Source: BMJ Open. 2021 Nov 30;11(11):e050886. doi: 10.1136/bmjopen-2021-050886 (PMC8634330; doi:10.1136/bmjopen-2021-050886)
Supplement: Supplementary data [file bmjopen-2021-050886supp003.pdf]

Appendix 3: Administrative information

Title

Best-BRA (Is subpectoral or pre-pectoral implant placement best in immediate breast reconstruction?): a protocol for a pilot randomised controlled trial of subpectoral versus pre-pectoral immediate implant-based breast reconstruction in women following mastectomy.

Trial registration number

ISRCTN: 15397185 (12 January 2021)

World Health Organization Trial Registration Data Set

Data category	Information
Primary registry and trial identifying number	ISRCTN 10081873
Date of registration in primary registry	12 January 2021
Secondary identifying numbers	IRAS: 279460<br>NHS REC: 20/WA/0338
Source(s) of monetary or material support	National Institute for Health Research (NIHR)
Primary sponsor	North Bristol NHS Trust
Secondary sponsor(s)	Not applicable
Contact for public queries	Dr Kirsty Roberts, bestbra-study@bristol.ac.uk, 07989981816
Contact for scientific queries	Ms Shelley Potter, shelley.potter@bristol.ac.uk, 0117 9287218
Public title	Best-BRA
Scientific title	Is subpectoral or pre-pectoral implant placement best in immediate breast reconstruction? The Best-BRA pilot RCT
Countries of recruitment	England, Wales, Scotland, Northern Ireland
Health condition(s) or problem(s) studied	Breast cancer
Intervention(s)	Subpectoral or pre-pectoral immediate implant-based breast reconstruction following mastectomy
Key inclusion and exclusion criteria	Inclusion: Women aged 18 or over; Require a mastectomy for breast cancer (invasive or preinvasive disease) or risk-reduction; Elect to undergo immediate IBBR; Considered eligible for either pre or subpectoral reconstruction by the surgical team.<br>Exclusion: Delayed reconstruction; Revisional surgery
Study type	Intervention
Date of first enrolment	To be determined

Target sample size	Pilot trial so not applicable
Recruitment status	Open to recruitment
Primary outcome(s)	Recruitment (number of sites recruiting; proportion of eligible women approached that are randomised, women recruited per site per month), adherence to trial allocation and outcomes completion rates will be reviewed at 12 months. At 12 months the parameters required for a fully powered trial will also be assessed (e.g number of sites and sample size).
Key secondary outcomes	Feasibility of collecting the following outcomes:<br>• The difference between groups in satisfaction with breasts using the validated BREAST-Q questionnaire at 12 months<br>• Differences between the groups in pain scores at 24 hours and 1 week<br>• The difference between groups in surgical complications, in particular implant loss, infection, re-admission and reoperation at 3 and 12 months<br>• The difference between groups in the need for additional surgery to the reconstruction or the contralateral breast at 12 months<br>• Differences between the groups in objective cosmetic outcome at 12 months assessed using patient photographs<br>• The difference between groups in their average EQ-5D-5L health related quality of life score and ICECAP-A scores at 1 year<br>• The difference between groups with respect to other key patient reported outcome domains included in the breast reconstruction core outcome set[1] including physical well-being; emotional well-being; and animation assessed using appropriate subscales of the BREAST-Q<br>• The cost effectiveness of pre-pectoral and subpectoral IBBR

Protocol version

Version 2.1 (16 March 2021)

Version		Notes
**Number**	**Date**	
2.1	16.03.21	This was approved by REC/HRA

Funding

NIHR

Contributorship

See main manuscript

Sponsor contact information

Trial sponsor: North Bristol NHS Trust
Sponsor's reference: 4716
Contact name: Mr Paolo Buscemi
Address: Research & Innovation, North Bristol NHS Trust, Level 3, Learning & Research building, Southmead Hospital, Westbury on Trym, Bristol, BS10 5NB
Tel: 0117 41 49345
E-mail: researchSponsor@nbt.nhs.uk

Role of study sponsor and funder

The funder and sponsor had no role in the design of this study and will not have any role during its execution, analyses, interpretation of the data, or decision to submit results.

Committees

The Trial Management Group (TMG) comprises all investigators, the trial manager, research and staff, with input from a patient/public representative. Members of the TMG will contribute to the trial in the following ways: trial design and methods; participant recruitment and trial conduct; trial management; trial logistics and cost management; economic evaluation; qualitative (QRI) study; statistical data analysis; and publication. The TMG will meet on a regular basis to oversee the management of the trial. The TMG will be provided with detailed information by the centre staff regarding trial progress. Meetings will be via videoconference facilities.

This study was designed and is being delivered in collaboration with the Bristol Trials Centre (BTC), a UKCRC registered clinical trials unit which is in receipt of National Institute for Health Research CTU support funding. Members of the BTC will attend the TMG.

Because this is a low-risk trial, the funder has agreed that the roles of both guiding the Trial Management Group and monitoring trial data will be undertaken by a single Trial Steering/Data Monitoring Committee (TS/DMC). The TS/DMC will meet at least three times over the course of the study and comprises three independent members: a chairperson, a statistician, and a patient representative. Their role will be to provide overall supervision of the trial on behalf of the funder, with a focus on progress of the trial, adherence to the protocol, patient safety and consideration of new information. The committee will review the accruing data and assess whether there are any safety issues that should be brought to the Sponsor's or the participants' attention or any reasons for the trial not to continue. Terms of reference will be drawn up and agreed with members of the TS/DMC.
