# Supplementary material for: Best-BRA (Is subpectoral or prepectoral implant placement best in immediate breast reconstruction?): a protocol for a pilot randomised controlled trial of subpectoral versus prepectoral immediate implant-based breast reconstruction in women following mastectomy
Source: BMJ Open. 2021 Nov 30;11(11):e050886. doi: 10.1136/bmjopen-2021-050886 (PMC8634330; doi:10.1136/bmjopen-2021-050886)
Supplement: Author's manuscript [file bmjopen-2021-050886.draft_revisions.pdf]

BMJ Open is committed to open peer review. As part of this commitment we make the peer review history of every article we publish publicly available.

When an article is published we post the peer reviewers' comments and the authors' responses online. We also post the versions of the paper that were used during peer review. These are the versions that the peer review comments apply to.

The versions of the paper that follow are the versions that were submitted during the peer review process. They are not the versions of record or the final published versions. They should not be cited or distributed as the published version of this manuscript.

BMJ Open is an open access journal and the full, final, typeset and author-corrected version of record of the manuscript is available on our site with no access controls, subscription charges or pay-per-view fees (http://bmjopen.bmj.com).

If you have any questions on BMJ Open's open peer review process please email info.bmjopen@bmj.com

BMJ Open

Best-BRA (Is subpectoral or pre-pectoral implant placement best in immediate breast reconstruction?) A protocol for a pilot randomised controlled trial of subpectoral versus pre-pectoral immediate implant-based breast reconstruction in women following mastectomy.

Journal:	*BMJ Open*
Manuscript ID	bmjopen-2021-050886
Article Type:	Protocol
Date Submitted by the Author:	03-Mar-2021
Complete List of Authors:	Roberts, Kirsty; University of Bristol Medical School, Population Health Sciences<br>Mills, Nicola; University of Bristol Medical School, Population Health Sciences<br>Metcalfe, Chris; University of Bristol Medical School, Population Health Sciences<br>Lane, Athene; University of Bristol Medical School, Population Health Sciences<br>Clement, Clare; University of Bristol Medical School, Population Health Sciences<br>Hollingworth, William; University of Bristol Medical School, Population Health Sciences<br>Taylor, Jodi; University of Bristol Medical School, Population Health Sciences<br>Holcombe, Chris; Royal Liverpool and Broadgreen University Hospitals NHS Trust, Linda McCartney Centre<br>Skillman, Joanna; University Hospitals Coventry and Warwickshire NHS Trust, Department of Plastic Surgery<br>Fairhurst, Katherine; University of Bristol Medical School, Population Health Sciences<br>Whisker, Lisa; Nottingham University Hospitals NHS Trust, Nottingham Breast Institute<br>Cutress, RI ; University of Southampton<br>Thrush, Steven; Worcestershire Acute Hospitals NHS Trust, Breast Unit<br>Fairbrother, Patricia; Independent Cancer Patients' Voice<br>Potter, Shelley; University of Bristol Medical School, Population Health Sciences; North Bristol NHS Trust, Bristol Breast Care Centre
Keywords:	Breast surgery < SURGERY, Breast tumours < ONCOLOGY, PLASTIC & RECONSTRUCTIVE SURGERY, QUALITATIVE RESEARCH

SCHOLARONE™
Manuscripts

I, the Submitting Author has the right to grant and does grant on behalf of all authors of the Work (as defined in the below author licence), an exclusive licence and/or a non-exclusive licence for contributions from authors who are: i) UK Crown employees; ii) where BMJ has agreed a CC-BY licence shall apply, and/or iii) in accordance with the terms applicable for US Federal Government officers or employees acting as part of their official duties; on a worldwide, perpetual, irrevocable, royalty-free basis to BMJ Publishing Group Ltd ("BMJ") its licensees and where the relevant Journal is co-owned by BMJ to the co-owners of the Journal, to publish the Work in this journal and any other BMJ products and to exploit all rights, as set out in our licence.

The Submitting Author accepts and understands that any supply made under these terms is made by BMJ to the Submitting Author unless you are acting as an employee on behalf of your employer or a postgraduate student of an affiliated institution which is paying any applicable article publishing charge ("APC") for Open Access articles. Where the Submitting Author wishes to make the Work available on an Open Access basis (and intends to pay the relevant APC), the terms of reuse of such Open Access shall be governed by a Creative Commons licence – details of these licences and which Creative Commons licence will apply to this Work are set out in our licence referred to above.

Other than as permitted in any relevant BMJ Author's Self Archiving Policies, I confirm this Work has not been accepted for publication elsewhere, is not being considered for publication elsewhere and does not duplicate material already published. I confirm all authors consent to publication of this Work and authorise the granting of this licence.

For peer review only

Best-BRA (Is subpectoral or pre-pectoral implant placement best in immediate breast reconstruction?) A protocol for a pilot randomised controlled trial of subpectoral versus pre-pectoral immediate implant-based breast reconstruction in women following mastectomy.

Kirsty Roberts[1], Nicola Mills[1], Chris Metcalfe[1], Athene Lane[1], Clare Clement[1], William Hollingworth[1], Jodi Taylor[1], Chris Holcombe[2], Jo Skillman[3], Katherine Fairhurst[4], Lisa Whisker[5], Ramsey I Cutress[6], Steven Thrush[7], Patricia Fairbrother[8], Shelley Potter[4,9].

[1]Population Health Sciences, Bristol Medical School, University of Bristol, Bristol, UK,

[2]Linda McCartney Centre, Royal Liverpool and Broadgreen University Hospital, Liverpool, UK,

[3] University Hospitals Coventry and Warwickshire NHS Trust, Coventry, UK,

[4] Bristol Centre for Surgical Research, Population Health Sciences, Bristol Medical School, University of Bristol, Bristol, UK,

[5] Nottingham Breast Institute, Nottingham University Hospitals NHS Trust, Nottingham, UK,

[6] University of Southampton, University Hospitals Southampton, Southampton, UK

[7]Breast Unit, Worcester Royal Hospital, Worcester, UK.

[8] Independent Cancer Patients' Voice, London, UK,

[9] Bristol Breast Care Centre, North Bristol NHS Trust, Bristol, UK.

Corresponding author

Shelley Potter, Associate Professor of Oncoplastic Breast Surgery

For peer review only - http://bmjopen.bmj.com/site/about/guidelines.xhtml

UOB Confidential

Postal address: Bristol Centre for Surgical Research, Population Health Sciences, Bristol Medical School, University of Bristol, Canynge Hall, 39 Whatley Road, Bristol BS8 2PS

Email: shelley.potter@bristol.ac.uk

Telephone: 0117 9287218

Keywords

Implant-based breast reconstruction, pre-pectoral, subpectoral, pilot trial, randomised controlled trial, Quintet Recruitment Intervention

For peer review only

For peer review only - http://bmjopen.bmj.com/site/about/guidelines.xhtml

UOB Confidential

Abstract

Background

Implant-based breast reconstruction (IBBR) is the most commonly performed reconstructive procedure following mastectomy. IBBR techniques are evolving rapidly, with mesh-assisted subpectoral reconstruction becoming the standard of care and more recently, pre-pectoral techniques being introduced. These muscle-sparing techniques may reduce post-operative pain, avoid implant animation and improve cosmetic outcomes and have been widely adopted into practice. Although small observational studies have failed to demonstrate any differences in the clinical or patient-reported outcomes of pre or subpectoral reconstruction, high-quality comparative evidence of clinical or cost-effectiveness is lacking. A well-designed, adequately powered randomised controlled trial (RCT) is needed to compare the techniques, but breast reconstruction RCTs are challenging. We, therefore, aim to undertake an external pilot RCT (Best-BRA) with an embedded QuinteT Recruitment Intervention (QRI) to determine the feasibility of undertaking a trial comparing pre and subpectoral techniques.

Methods and analysis

Best-BRA is a pragmatic, two-arm, external pilot randomised controlled trial with an embedded QRI and economic scoping for resource use. Women who require a mastectomy for either breast cancer or risk-reduction, elect to have an IBBR and are considered suitable for both pre and subpectoral reconstruction will be recruited and randomised 1:1 between the techniques.

The QRI will be implemented in two phases: Phase 1, in which sources of recruitment difficulties are rapidly investigated to inform the delivery in Phase 2 of tailored interventions to optimise recruitment of patients.

For peer review only - http://bmjopen.bmj.com/site/about/guidelines.xhtml

UOB Confidential

Primary outcomes will be i) recruitment of patients, ii) adherence to trial allocation and iii) outcome completion rates. Outcomes will be reviewed at 12 months to determine the feasibility of a definitive trial.

Ethics and dissemination

The study has been approved by the NHS Wales REC 6 (20/WA/0338). Findings will be presented at conferences and in peer-reviewed journals.

Trial registration

ISRCTN10081873.

Word Count: 289/300

Strengths and Limitations of this study

- This external pilot RCT with an embedded QuinteT Recruitment Intervention will determine whether it is possible to recruit and randomise patients to a trial comparing pre and subpectoral approaches for implant-based breast reconstruction.
- The QuinteT Recruitment Intervention will allow recruitment challenges to be identified, understood, and addressed in real-time, allowing a rapid decision about the feasibility of a definitive trial.
- The external pilot RCT will only address the feasibility of recruitment within an implant reconstruction study; a full-scale main study will still be necessary to compare the clinical and cost-effectiveness of pre and subpectoral implant-based breast reconstruction.

For peer review only - http://bmjopen.bmj.com/site/about/guidelines.xhtml

UOB Confidential

For peer review only

Introduction

55,000 women per year in the UK are diagnosed with breast cancer (1) of whom 40% will require a mastectomy (2). The loss of a breast may dramatically impact women's quality of life (3) and in the UK, the National Institute for Health and Care Excellence (NICE) recommends offering immediate breast reconstruction to improve outcomes (4).

Implant-based breast reconstruction (IBBR) is the most commonly performed breast reconstruction procedure in the UK accounting for almost 70% of all immediate reconstructions performed following mastectomy (5). The introduction of biological or synthetic mesh over the last 10 years has had a major impact on the practice of IBBR. Initially, mesh was sutured between the lower border of the pectoralis muscle and the chest wall extending the subpectoral pocket (6). This allowed a definitive fixed-volume implant to be placed under the muscle at the time of surgery, avoiding the need for tissue expansion and a second procedure and resulting in better cosmetic outcomes through improved lower pole projection (7-14). Although there was limited data to support the safety or effectiveness of mesh-assisted subpectoral techniques (15-19), they became established as the standard of care in the UK (18). More recently, pre-pectoral techniques have been introduced in which the implant, fully or partially wrapped in mesh, is placed on top of rather than under the pectoralis major muscle (20). These 'muscle-sparing' techniques may be less painful and avoid the potentially distressing implant 'animation' seen when the pectoralis muscle contracts (21).

Due to the perceived advantages of the procedure, pre-pectoral reconstruction is being widely adopted into practice without high-quality evidence to support its use. Early results are promising (22-29), but caution is required as subcutaneous implant reconstruction without mesh was previously abandoned by the reconstructive community due to high complication rates (30-33). Few studies have directly compared the outcomes of pre and subpectoral mesh-assisted techniques (18, 34-38). These are small, often single centre studies with limited follow up, but have demonstrated

For peer review only - http://bmjopen.bmj.com/site/about/guidelines.xhtml

UOB Confidential

For peer review only

no differences in short-term clinical (34-37) or patient-reported (35) outcomes between pre and subpectoral implant placement. The iBRA prospective multicentre cohort study demonstrated that the short-term complications of pre and subpectoral reconstruction were equivalent (18) but suggested that at 18 months, satisfaction with outcome, assessed using the validated BREAST-Q, may be greater in patients receiving pre-pectoral compared with subpectoral techniques (19). Caution is required, however as the numbers of patients undergoing pre-pectoral reconstruction in the iBRA study were small.

High-quality evidence is lacking and there is therefore a need for a well-designed, adequately powered randomised controlled trial (RCT) to evaluate the clinical and cost-effectiveness of IBBR techniques. RCTs in breast reconstruction however are challenging due to patient (39) and surgeon preference (40) and expert opinion suggesting that RCTs in breast reconstruction would be 'unethical', 'impractical' and/or 'inappropriate'(3, 41-44). Previous trials in breast reconstruction have been unsuccessful due to failure to recruit (39, 45). Careful feasibility work to inform the design and conduct of a future study is therefore required before a definitive trial can be planned.

The UK iBRA study (46) used mixed-methods including a national practice questionnaire (47, 48), a randomisation acceptability survey (49) and semi-structured interviews (50) with key stakeholders to explore the most appropriate design for a future RCT. This work suggested that a well-designed trial would be feasible and that key areas of uncertainty included the use of biological vs. synthetic mesh and pre vs. subpectoral implant positioning (49). Pre-pectoral techniques, however, only emerged during the iBRA study and have gained popularity since with two-thirds of survey respondents now offering the technique. Ongoing work in the Pre-BRA study, an IDEAL 2a/2b evaluating the safety and stability of pre-pectoral reconstruction prior to definitive evaluation suggests that the technique is safe and sufficiently stable for evaluation in a clinical trial (51).

While the majority of respondents felt that an appropriately designed RCT in IBBR may be possible (49), qualitative interviews with key stakeholders identified a number of barriers to the successful

For peer review only - http://bmjopen.bmj.com/site/about/guidelines.xhtml
UOB Confidential

conduct of a future trial (50). These included limited understanding of a pragmatic study design and the role of randomisation in minimising bias, issues around clinician equipoise and aspects of surgical culture not being supportive of RCTs. Previous work has shown that embedding qualitative methods such as the QuinteT Recruitment Intervention (QRI) within an RCT can allow these issues to be effectively addressed and overcome. The QRI has facilitated successful completion of several surgical trials that had previously been considered impossible (52, 53). Adopting this approach in any future IBBR RCT would therefore be vital to identify, understand and address recruitment issues in this context to optimise the success of the trial.

Although the iBRA study addressed many of the feasibility issues relating to a future trial including the most appropriate comparators, it did not include a randomised study. It is therefore not clear whether surgeons will recruit patients to an RCT comparing two approaches to IBBR or whether patients will consent to be randomised and agree to receive their allocated treatment. Further feasibility work is therefore needed.

We aimed to undertake an external pilot RCT (Best-BRA study- is subpectoral or pre-pectoral implant placement Best in immediate BReAst reconstruction?) with an embedded QuinteT Recruitment Intervention to determine whether it is possible to recruit and randomise women to a study comparing pre and subpectoral implant-based breast reconstruction to assess the feasibility of progression to a definitive large-scale trial.

Methods

This protocol adheres to the Standard Protocol Items for Randomised Trials (SPIRIT) (54) guidelines.

For peer review only - http://bmjopen.bmj.com/site/about/guidelines.xhtml

UOB Confidential

For peer review only

Study Design

The study will consist of a pragmatic multi-centre external pilot RCT with an embedded QRI to determine the feasibility of recruitment and randomisation to either a subpectoral or pre-pectoral implant reconstruction following mastectomy.

Study Setting

NHS breast and plastic surgery units in the UK.

Recruitment

Participants will be recruited and randomised over a 12-month period. All female patients undergoing mastectomy who elect to have an IBBR will form the target population. Each site will maintain a trial screening log following the SEAR framework (55) to determine the proportion of:

i. Patients considered eligible for the study (with reasons for non-eligibility)

ii. Eligible patients approached about the study (with reasons for not being approached)

iii. Eligible patients randomised (with reasons for not being randomised)

iv. Randomised patients who receive their allocated treatment (with reasons for not receiving allocated treatments)

The screening log will be reviewed monthly to provide feedback to recruiters and aid understanding of surgeons' and patients' preferences for types of surgery as part of the QRI.

Potential participants who require a mastectomy for breast cancer or risk-reduction will be identified from breast cancer and oncoplastic multidisciplinary team meetings (MDTs) (Figure 1). The study will be introduced when reconstructive options are discussed, and patients will be given written

For peer review only - http://bmjopen.bmj.com/site/about/guidelines.xhtml

UOB Confidential

For peer review only

information outlining the study. As a key objective of the study is to understand recruitment challenges, these initial consultations will be recorded with patient consent.

Women will provide written consent prior to study participation. This will include optional consent to be contacted for future studies, and consent to assess long-term patient reported outcomes through linkage to routine data sources.

Eligibility and Allocation

Inclusion criteria

Patients will be included for the trial if they meet all of the following criteria:

1) They are aged 18 years or above.
2) They require a mastectomy for breast cancer or risk-reduction.
3) They elect to undergo immediate IBBR.
4) They are considered eligible for both pre and subpectoral reconstruction by the surgical team.

Exclusion criteria

Patients will be excluded from the trial if they meet any of the following criteria:

1) They have delayed reconstruction
2) They are having revision breast reconstruction surgery

Patients who smoke, have high body mass index (BMI) or have had previous radiotherapy to the breast will not be excluded for participation in the trial but surgeons will be encouraged to offer mesh-assisted IBBR in line with joint Association of Breast Surgery (ABS) and British Association of Plastic, Reconstructive and Aesthetic Surgeons (BAPRAS)' guidelines for the use of mesh in reconstructive procedures (56).

For peer review only - http://bmjopen.bmj.com/site/about/guidelines.xhtml

UOB Confidential

For peer review only

Participants will be randomised after eligibility and consent have been confirmed at the final clinic visit prior to admission to surgery. The randomisation sequence will be generated by a statistician independent of participant recruitment using the random number generator in Stata statistical software (version 16, StataCorp, College Station, Texas, 2019). Patients will be randomly allocated to the techniques in a 1:1 ratio to either subpectoral or pre-pectoral immediate IBBR stratified by hospital. Women undergoing bilateral surgery will receive the same procedure on both sides. Allocation will be concealed until the patient has been logged into the system and a study ID number generated so ensuring that judgements about eligibility are made without knowledge of the next allocation.

Intervention

All patients will undergo a skin or nipple-preserving, or skin-reducing mastectomy followed by an IBBR. Participating surgeons will undertake the procedure as per their standard practice. Mesh choice (biological or synthetic and the product used) and implant selection (fixed volume; adjustable implants or tissue expanders) will be as per surgeon preference. Two surgeon/two team operating (both procedures performed simultaneously) will be encouraged in bilateral cases to minimise operative time. The following steps of the IBBR procedure will be considered mandatory, prohibited and flexible/discretionary according to the typology described by Blencowe et al (57)**.**

- **Mandatory** – Insertion of a tissue expander/adjustable implant or fixed volume implant
- **Prohibited** –Raising the pectoralis muscle **IF allocated to pre-pectoral trial arm**
- **Flexible/discretionary** – all other steps of the procedure

Standard Care during and post procedure

Strategies to minimise infection (e.g. use of laminar flow, cavity irrigation, glove change) will be as per local practice but participating centres will be encouraged to adhere to best practice (58) and

For peer review only - http://bmjopen.bmj.com/site/about/guidelines.xhtml
UOB Confidential
For peer review only

use the evidence-based Manchester "TIC List" wherever possible (59). Use of drains and other concomitant interventions will be permitted as per local practice. Post-operative complications will be assessed using internationally agreed standardised definitions (60) by local clinical teams at routine postoperative hospital visits i.e., routine standard of care.

Clinically relevant complications assessed at 3 months and 12 months include:

- Implant loss defined as removal of the expander/implant without replacement
- Infection requiring treatment with antibiotics and/or surgical debridement
- Unplanned re-operation for complications relating to the implant reconstruction
- Readmission to hospital for complications related to the implant reconstruction

Outcome measures

In the definitive trial, the proposed primary outcome will be women's satisfaction with the cosmetic outcome of their reconstruction at 12 months following surgery assessed using the 'Satisfaction with Breasts' domain of the validated BREAST-Q questionnaire (61). On this basis, for the main trial, 100 patients will be required in each group to allow a true 0.5 standard deviation difference in the BREAST-Q 'Satisfaction with Breasts' domain scale to be detected at 5% significance and 90% power, assuming more than 85% of participants complete the questionnaire 12-months following surgery.

The purpose of this study is to determine the feasibility of conducting a future main trial, specifically whether it will be possible to recruit and randomise 200 patients undergoing IBBR and to collect the required data for the main trial.

The **primary outcomes** for the Best-BRA study are therefore

i) recruitment (number of sites recruiting; proportion of eligible women approached that are randomised, women recruited per site per month)

ii) Adherence to trial allocation

For peer review only - http://bmjopen.bmj.com/site/about/guidelines.xhtml

UOB Confidential

iii) Outcome completion rates at 3 and 12 months.

Secondary outcomes will be the feasibility of collecting the proposed primary and secondary outcomes for the main study. These will include:

i. Satisfaction with breasts using the validated BREAST-Q questionnaire at 12 months. We anticipate that this will be the primary outcome for the definitive trial
ii. Surgical complications, in particular implant loss, infection, re-admission and reoperation within 3 and 12 months of random allocation
iii. The need for additional surgery to the reconstruction or the contralateral breast within 12 months of random allocation
iv. Pain scores assessed using a 10 point Likert scale at 24 hours and 1 week
v. Objective panel assessment of cosmetic outcome at 12 months assessed using routinely collected patient photographs
vi. EQ-5D-5L health related quality of life score (62) and ICECAP-A capability scores (63) at 1 year
vii. Other key patient-reported outcome domains included in the breast reconstruction core outcome set (64) including physical well-being (chest); emotional well-being; and animation assessed using appropriate subscales of the BREAST-Q
viii. The costs of pre-pectoral and subpectoral IBBR procedures

Data collection

Schedule of assessments are summarised in Table 2. All data will be entered directly into electronic case report forms (CRFs) by local research teams and patients will complete electronic patient reported outcome measures (PROMs) on REDCap (65). The feasibility of uploading routinely taken anonymised pre- and post-operative photographs directly onto the REDCap database following

For peer review only
For peer review only - http://bmjopen.bmj.com/site/about/guidelines.xhtml
UOB Confidential

specific patient consent will be explored. Reminders will be sent to participants by email/text message for up to 4 weeks if questionnaires at 3 and 12 months have not been completed.

Participants can choose to discontinue their participation in the trial for any reason. With participant consent, research data obtained up to the point of discontinuation will be retained for analysis. Participants who decide to stop completing patient reported outcome measures will continue to be followed up for complications and adverse event reporting through the review of their medical records unless they specifically object. Participants who are unwilling or unable to go ahead with their allocated treatment will be encouraged to continue in trial follow-up as per protocol and their alternative treatment recorded by the clinical staff team.

Sample size calculation

A formal sample size calculation is not required for this external pilot study. At 12 months, the number of sites recruiting; proportion of eligible women that are approached and randomised; the number of women recruited per site per month will be reviewed and adherence to trial allocation will be reviewed. If the study has opened and is successfully recruiting at an acceptable number of sites with high adherence to treatment allocation and follow up rates, a main trial will be considered feasible and parameters for the full study determined.

Statistical Analysis

Screening, recruitment, adherence to allocated procedure and completeness of primary outcome data will be presented by study group as a CONSORT flow chart, overall and per centre. Secondary outcome measures will be presented as summary statistics at each assessment point. Summary statistics for the two study groups combined will be presented at the end of the pilot phase if the study continues into a definitive trial, with pilot data included in the definitive trial analysis.

For peer review only - http://bmjopen.bmj.com/site/about/guidelines.xhtml

UOB Confidential

For peer review only

Health Economics

The feasibility of applying a novel micro-costing framework to compare the relative costs of pre-pectoral and subpectoral IBBR will be explored (66). A process map will be developed to track the patient pathway from first reconstruction consultation to last routine postoperative visit (usually up to 4 weeks following surgery). The process map will include a list of resources, equipment, consumables, and implants involved in the procedures. Process mapping will be undertaken at several sites to ensure that all appropriate factors and patient pathways are considered. Resources that may potentially differ between the two procedures (e.g. size of mesh; operative time; length of stay and number of postoperative visits) will be identified by reviewing the process maps and details collected on the CRFs. Data may also be gathered from review of electronic notes/medical records. National (where available) and local unit costs will be used to estimate the incremental cost difference between the two procedures.

QuinteT Recruitment Intervention (QRI)

A QRI will be embedded within the Best-BRA study to optimise recruitment and informed consent. The QRI will be implemented in two key phases – Phase 1, in which sources of recruitment difficulties are rapidly investigated to inform the delivery in Phase 2 of tailored interventions to optimise recruitment. This will be supplemented with up-front pre-emptive recruitment training in study set up, tailored to anticipated issues and informed by previous QRIs (40, 50, 53, 67, 68).

A multi-faceted, flexible approach will be adopted in Phase 1 using one or more of the following methods:

- Mapping of eligibility and recruitment pathways to collate basic data about the levels of eligibility and recruitment, and identify points at which patients opt in or out of the study;

For peer review only - http://bmjopen.bmj.com/site/about/guidelines.xhtml
UOB Confidential

- In-depth, semi-structured interviews with a purposive sample of staff/site members involved with aspects of study design/management and recruitment across centres. Interviews will explore perspectives on the RCT and issues around recruitment, including views about the study design and protocol, treatment options, existing evidence, and current practice. Interviews may also be undertaken with eligible patients to explore their views on the study, treatment options and provision of information. Interview topic guides will be used to ensure similar topic areas are covered across interviews, while still providing the scope for participants to raise issues of pertinence to them.
- Recording of consultations between healthcare staff and potentially eligible patients in which the study is discussed to explore information provision in relation to key study concepts and treatment options, recruitment techniques, engagement with patient treatment preferences, and randomisation decisions to identify recruitment difficulties and improve information provision. Consultations will be listened to and qualitatively analysed, documenting instances such as unclear, insufficient or imbalanced information provision and unintentional transferring of clinician treatment preferences to patients, as well as aspects of good practice.
- Attendance at Trial Management Group (TMG) and investigator meetings to gain an overview of trial conduct and overarching challenges.

An account of the anonymised findings from all the data will be fed back to the Chief Investigator (CI). The QRI team, with the CI and TMG, will formulate a plan of action grounded in these findings to improve recruitment and information provision, with its format dependent on the nature of recruitment barriers identified (Phase 2). Supportive and responsive group or individual feedback and training is likely to be a core component of the plan of action, including written recruitment ‘tips’ documents and suggested modifications to study pathways and patient-facing study material.

For peer review only - http://bmjopen.bmj.com/site/about/guidelines.xhtml

UOB Confidential

Phases 1 and 2 will be undertaken in an iterative and cyclical manner, continuing throughout the recruitment period with close monitoring of changes in screening log data and recruiter practice to optimise recruitment and informed consent, all in close collaboration with the CI and wider study team.

Data Management

Data will be entered onto the study REDCap database (65). The system will incorporate data entry and validation rules to reduce data entry errors, and management functions to facilitate auditing and data quality assurance. The Chief Investigator (CI) will have access to and act as custodian of the full dataset. Participants' data will be held securely held on the in line with data protection legislation. Personal data will not be kept for longer than is required for the purpose for which it has been acquired. Data will be held in compliance with the sponsor's standard operating procedures. Anonymous data sets will be made 'open data' following publication and stored in the University of Bristol's Research Data storage facility.

Monitoring, Safety and Audit

As this is a low-risk external pilot trial, comparing two procedures both of which are in routine clinical practice, the trial is overseen and audited by an independent joint Trial Steering/Data Monitoring Committee (TS/DMC). The TSC/DMC will comprise of an independent clinician, trials statistician, and PPI member. The TSC/DMC will meet once prior to recruitment of the first participant and convene at least annually to review adverse event data and any other ethical aspects that arise.

Research sites are responsible for reporting serious adverse events (SAEs) for their trial participants during the course of the trial on the electronic CRFs. The following adverse events are expected after surgery in this patient population:

- Anaesthetic complications, e.g. stroke or cardiac events such as myocardial infarction

For peer review only - http://bmjopen.bmj.com/site/about/guidelines.xhtml

UOB Confidential

- Return to theatre or readmission for complications of surgery
- Wound infection requiring treatment with antibiotics, readmission or reoperation
- Postoperative nausea and vomiting
- Pain
- Other infections (sepsis, septicaemia, abscess, respiratory)
- Other procedure specific complication including implant loss, mastectomy skin flap necrosis, infection, seroma
- Allergic or anaphylactic reaction of Patent V blue dye used for sentinel node biopsy

Adverse events will be documented and reported in accordance with the Sponsor's (North Bristol NHS Trust) Safety Reporting Standard Operating Procedure (SOP).

Protocol Amendments

Any amendments to the protocol will be reported to the appropriate regulatory bodies, with a full copy of the current protocol available for download from the study website. There are no amendments to date.

Ancillary and post-trial care

Participants will be treated according to standard care beyond their 12 months in the study.

Public and Patient Involvement (PPI)

PPI members sit on the Trial oversight committees and members of Independent Cancer Patients Voice have provided additional PPI input into all aspects of the study. PPI representatives have been actively involved in all stages of study design including the development of patient information leaflets and planning and refining recruitment strategies. Specifically, they have advised on how and

For peer review only - http://bmjopen.bmj.com/site/about/guidelines.xhtml

UOB Confidential

when to approach women undergoing IBBR to minimise the burden of participation and on the selection of outcome measures. PPI involvement will ensure that the patients' perspective is considered and remains the focus of the study. The PPI members will also assist in writing lay summaries and sharing findings of the study with a wider audience. They will advise on methods and content of communication with participants including newsletters and social media.

Ethics and dissemination

The NHS Wales Research Ethics Committee 6 (20/WA/0338) reviewed and approved the study. Potential participants will be given at least 24 hours to decide whether they wish to take part and will provide written informed consent (either in person or electronically using the REDCap e-consent function) prior to entering the trial. Specific consent will be obtained for i) upload of pre and postoperative clinical photographs; ii) future contact for long-term follow up with PROMs or via routinely collected data and iii) the collection of anonymised clinical data about their care that will be kept indefinitely after the close of the study as publicly "open data" to support future research studies.

Results of the study will be presented at national and international meetings and published in peer reviewed journals. We will work with our PPI contributors to produce study summaries for patients. If the study is feasible, further funding will be sought for a full-scale trial comparing pre and subpectoral implant-based reconstructive techniques.

Trial Status: Site set-up

Protocol: Version 2.0, 5th January 2021

Open to recruitment: No

Planned recruitment duration: 12 months

For peer review only - http://bmjopen.bmj.com/site/about/guidelines.xhtml

UOB Confidential

References

1. UK CR. Breast Cancer Statistics [Website]. 2018 [updated Accessed 18th Nov 2018. Available from: http://www.cancerresearchuk.org/health-professional/cancer-statistics/statistics-by-cancer-type/breast-cancer.

2. Malata CM, McIntosh SA, Purushotham AD. Immediate breast reconstruction after mastectomy for cancer. The British journal of surgery. 2000;87(11):1455-72.

3. Harcourt DM, Rumsey NJ, Ambler NR, Cawthorn SJ, Reid CD, Maddox PR, et al. The psychological effect of mastectomy with or without breast reconstruction: a prospective, multicenter study. Plast Reconstr Surg. 2003;111(3):1060-8.

4. NICE. Early and locally advanced breast cancer: diagnosis and management NICE guideline [NG101] 2018 [Available from: https://www.nice.org.uk/guidance/ng101.

5. Mennie JC, Mohanna PN, O'Donoghue JM, Rainsbury R, Cromwell DA. National trends in immediate and delayed post-mastectomy reconstruction procedures in England: A seven-year population-based cohort study. Eur J Surg Oncol. 2017;43(1):52-61.

6. Salzberg CA. Nonexpansive immediate breast reconstruction using human acellular tissue matrix graft (AlloDerm). Annals of Plastic Surgery. 2006;57(1):1-5.

7. Salzberg CA. Nonexpansive immediate breast reconstruction using human acellular tissue matrix graft (AlloDerm). Ann Plast Surg. 2006;57(1):1-5.

8. Gamboa-Bobadilla GM. Implant breast reconstruction using acellular dermal matrix. Ann Plast Surg. 2006;56(1):22-5.

9. Cassileth L, Kohanzadeh S, Amersi F. One-stage immediate breast reconstruction with implants: a new option for immediate reconstruction. Ann Plast Surg. 2012;69(2):134-8.

10. Breuing KH, Colwell AS. Inferolateral AlloDerm hammock for implant coverage in breast reconstruction. Ann Plast Surg. 2007;59(3):250-5.

11. Breuing KH, Warren SM. Immediate bilateral breast reconstruction with implants and inferolateral AlloDerm slings. Ann Plast Surg. 2005;55(3):232-9.

12. Zienowicz RJ, Karacaoglu E. Implant-based breast reconstruction with allograft. Plast Reconstr Surg. 2007;120(2):373-81.

13. Namnoum JD. Expander/implant reconstruction with AlloDerm: recent experience. Plast Reconstr Surg. 2009;124(2):387-94.

14. Ashikari RH, Ashikari AY, Kelemen PR, Salzberg CA. Subcutaneous mastectomy and immediate reconstruction for prevention of breast cancer for high-risk patients. Breast Cancer. 2008;15(3):185-91.

15. Dikmans REG, Negenborn VL, Bouman M-B, Winters HAH, Twisk JWR, Ruhé PQ, et al. Two-stage implant-based breast reconstruction compared with immediate one-stage implant-based breast reconstruction augmented with an acellular dermal matrix: an open-label, phase 4, multicentre, randomised, controlled trial. The Lancet Oncology. 2017;18(2):251-8.

16. Lohmander F, Lagergren J, Johansson H, Roy PG, Frisell J, Brandberg Y. Quality of life and patient satisfaction after implant-based breast reconstruction with or without acellular dermal matrix: randomized clinical trial. BJS Open. 2020;4(5):811-20.

For peer review only - http://bmjopen.bmj.com/site/about/guidelines.xhtml

UOB Confidential

17. Lohmander F, Lagergren J, Roy PG, Johansson H, Brandberg Y, Eriksen C, et al. Implant Based Breast Reconstruction With Acellular Dermal Matrix: Safety Data From an Open-label, Multicenter, Randomized, Controlled Trial in the Setting of Breast Cancer Treatment. Ann Surg. 2019;269(5):836-41.

18. Potter S, Conroy EJ, Cutress RI, Williamson PR, Whisker L, Thrush S, et al. Short-term safety outcomes of mastectomy and immediate implant-based breast reconstruction with and without mesh (iBRA): a multicentre, prospective cohort study. Lancet Oncol. 2019;20(2):254-66.

19. Sewart E, Turner NL, Conroy EJ, Cutress RI, Skillman J, Whisker L, et al. Patient-reported outcomes of immediate implant-based breast reconstruction with and without biological or synthetic mesh. BJS Open. 2021;5(1).

20. Tasoulis MK, Iqbal FM, Cawthorn S, MacNeill F, Vidya R. Subcutaneous implant breast reconstruction: Time to reconsider? European Journal of Surgical Oncology (EJSO). 2017;43(9):1636-46.

21. Cattelani L, Polotto S, Arcuri MF, Pedrazzi G, Linguadoca C, Bonati E. One-Step Prepectoral Breast Reconstruction With Dermal Matrix-Covered Implant Compared to Submuscular Implantation: Functional and Cost Evaluation. Clin Breast Cancer. 2018;18(4):e703-e11.

22. Berna G, Cawthorn SJ, Papaccio G, Balestrieri N. Evaluation of a novel breast reconstruction technique using the Braxon((R)) acellular dermal matrix: a new muscle-sparing breast reconstruction. ANZ J Surg. 2017;87(6):493-8.

23. Reitsamer R, Peintinger F. Prepectoral implant placement and complete coverage with porcine acellular dermal matrix: a new technique for direct-to-implant breast reconstruction after nipple-sparing mastectomy. J Plast Reconstr Aesthet Surg. 2015;68(2):162-7.

24. Casella D, Bernini M, Bencini L, Roselli J, Lacaria MT, Martellucci J, et al. TiLoop(R) Bra mesh used for immediate breast reconstruction: comparison of retropectoral and subcutaneous implant placement in a prospective single-institution series. Eur J Plast Surg. 2014;37(11):599-604.

25. Casella D, Calabrese C, Bianchi S, Meattini I, Bernini M. Subcutaneous Tissue Expander Placement with Synthetic Titanium-Coated Mesh in Breast Reconstruction: Long-term Results. Plast Reconstr Surg Glob Open. 2015;3(12):e577.

26. Bernini M, Calabrese C, Cecconi L, Santi C, Gjondedaj U, Roselli J, et al. Subcutaneous Direct-to-Implant Breast Reconstruction: Surgical, Functional, and Aesthetic Results after Long-Term Follow-Up. Plast Reconstr Surg Glob Open. 2015;3(12):e574.

27. Becker H, Lind JG, 2nd, Hopkins EG. Immediate Implant-based Prepectoral Breast Reconstruction Using a Vertical Incision. Plast Reconstr Surg Glob Open. 2015;3(6):e412.

28. Vidya R, Masia J, Cawthorn S, Berna G, Bozza F, Gardetto A, et al. Evaluation of the effectiveness of the prepectoral breast reconstruction with Braxon dermal matrix: First multicenter European report on 100 cases. Breast J. 2017;23(6):670-6.

29. Chandarana M, Harries S, Group NBAS. Multicentre study of prepectoral breast reconstruction using acellular dermal matrix. BJS Open. 2020;4:71-7.

For peer review only

For peer review only - http://bmjopen.bmj.com/site/about/guidelines.xhtml
UOB Confidential

30. Snyderman RK, Guthrie RH. Reconstruction of the female breast following radical mastectomy. Plast Reconstr Surg. 1971;47(6):565-7.

31. Gruber RP, Kahn RA, Lash H, Maser MR, Apfelberg DB, Laub DR. Breast reconstruction following mastectomy: a comparison of submuscular and subcutaneous techniques. Plast Reconstr Surg. 1981;67(3):312-7.

32. Rebowe RE, Allred LJ, Nahabedian MY. The Evolution from Subcutaneous to Prepectoral Prosthetic Breast Reconstruction. Plast Reconstr Surg Glob Open. 2018;6(6):e1797.

33. Wagner RD, Braun TL, Zhu H, Winocour S. A systematic review of complications in prepectoral breast reconstruction. J Plast Reconstr Aesthet Surg. 2019;72(7):1051-9.

34. Bettinger LN, Waters LM, Reese SW, Kutner SE, Jacobs DI. Comparative Study of Prepectoral and Subpectoral Expander-Based Breast Reconstruction and Clavien IIIb Score Outcomes. Plastic and reconstructive surgery Global open. 2017;5(7):e1433-e.

35. Baker BG, Irri R, MacCallum V, Chattopadhyay R, Murphy J, Harvey JR. A Prospective Comparison of Short-Term Outcomes of Subpectoral and Prepectoral Strattice-Based Immediate Breast Reconstruction. Plast Reconstr Surg. 2018;141(5):1077-84.

36. Chandarana MN, Jafferbhoy S, Marla S, Soumian S, Narayanan S. Acellular dermal matrix in implant-based immediate breast reconstructions: a comparison of prepectoral and subpectoral approach. Gland surgery. 2018;7(Suppl 1):S64-S9.

37. Thangarajah F, Treeter T, Krug B, Hellmich M, Eichler C, Hanstein B, et al. Comparison of Subpectoral versus Prepectoral Immediate Implant Reconstruction after Skin- and Nipple-Sparing Mastectomy in Breast Cancer Patients: A Retrospective Hospital-Based Cohort Study. Breast Care (Basel). 2019;14(6):382-7.

38. Manrique OJ, Banuelos J, Abu-Ghname A, Nguyen M-D, Tran NV, Martinez-Jorge J, et al. Surgical Outcomes of Prepectoral Versus Subpectoral Implant-based Breast Reconstruction in Young Women. Plastic and reconstructive surgery Global open. 2019;7(3):e2119-e.

39. Winters ZE, Emson M, Griffin C, Mills J, Hopwood P, Bidad N, et al. Learning from the QUEST multicentre feasibility randomization trials in breast reconstruction after mastectomy. The British journal of surgery. 2015;102(1):45-56.

40. Potter S, Mills N, Cawthorn SJ, Donovan J, Blazeby JM. Time to be BRAVE: is educating surgeons the key to unlocking the potential of randomised clinical trials in surgery? A qualitative study. Trials. 2014;15:80.

41. Mullan MH, Wilkins EG, Goldfarb S, Lowery JC, Smith DM, Wickman M, et al. Prospective analysis of psychosocial outcomes after breast reconstruction: cross-cultural comparisons of 1-year postoperative results. J Plast Reconstr Aesthet Surg. 2007;60(5):503-8.

42. Atisha D, Alderman AK, Lowery JC, Kuhn LE, Davis J, Wilkins EG. Prospective analysis of long-term psychosocial outcomes in breast reconstruction: two-year postoperative results from the Michigan Breast Reconstruction Outcomes Study. Ann Surg. 2008;247(6):1019-28.

For peer review only - http://bmjopen.bmj.com/site/about/guidelines.xhtml

UOB Confidential

43. Alderman AK, Wilkins EG, Kim HM, Lowery JC. Complications in postmastectomy breast reconstruction: two-year results of the Michigan Breast Reconstruction Outcome Study. Plast Reconstr Surg. 2002;109(7):2265-74.

44. Fogarty BJ, Brown AP, Miller R, Khan K. TRAM flap versus nonautologous breast reconstruction: what do patients really think? Plast Reconstr Surg. 2004;113(4):1146-52.

45. McCarthy CM, Lee CN, Halvorson EG, Riedel E, Pusic AL, Mehrara BJ, et al. The use of acellular dermal matrices in two-stage expander/implant reconstruction: a multicenter, blinded, randomized controlled trial. Plast Reconstr Surg. 2012;130(5 Suppl 2):57S-66S.

46. Potter S, Conroy E, Williamson P, Thrush S, Whisker LJ, Skillman JM, et al. The iBRA (implant Breast Reconstruction evAluation) Study: Protocol for a prospective multicentre cohort study to inform the feasibility, design and conduct of a pragmatic randomised clinical trial comparing new techniques of implant-based breast reconstruction. . Pilot and Feasibility Studies. 2016;in press.

47. Mylvaganam S, Conroy EJ, Williamson PR, Barnes NLP, Cutress RI, Gardiner MD, et al. Adherence to best practice consensus guidelines for implant-based breast reconstruction: Results from the iBRA national practice questionnaire survey. Eur J Surg Oncol. 2018;44(5):708-16.

48. Mylvaganam S, Conroy E, Williamson PR, Barnes NLP, Cutress RI, Gardiner MD, et al. Variation in the provision and practice of implant-based breast reconstruction in the UK: Results from the iBRA national practice questionnaire. Breast. 2017;35:182-90.

49. The i BRASG. P106. Appropriately-designed randomised trials may be an acceptable method for addressing uncertainties in implant-based breast reconstruction: Preliminary findings from the iBRA Randomisation Acceptability Survey. European Journal of Surgical Oncology. 2019;45(5):912.

50. Davies G, Mills N, Holcombe C, Potter S, i BRASG. Perceived barriers to randomised controlled trials in breast reconstruction: obstacle to trial initiation or opportunity to resolve? A qualitative study. Trials. 2020;21(1):316.

51. Harvey KL, Mills N, White P, Holcombe C, Potter S. The Pre-BRA (pre-pectoral Breast Reconstruction EvAluation) feasibility study: protocol for a mixed-methods IDEAL 2a/2b prospective cohort study to determine the safety and effectiveness of prepectoral implant-based breast reconstruction. 2020;10(1):e033641.

52. Donovan JL, Rooshenas L, Jepson M, Elliott D, Wade J, Avery K, et al. Optimising recruitment and informed consent in randomised controlled trials: the development and implementation of the Quintet Recruitment Intervention (QRI). Trials. 2016;17(1):283.

53. Rooshenas L, Scott LJ, Blazeby JM, Rogers CA, Tilling KM, Husbands S, et al. The QuinteT Recruitment Intervention supported five randomized trials to recruit to target: a mixed-methods evaluation. Journal of clinical epidemiology. 2019;106:108-20.

54. Chan AW, Tetzlaff JM, Gotzsche PC, Altman DG, Mann H, Berlin JA, et al. SPIRIT 2013 explanation and elaboration: guidance for protocols of clinical trials. Bmj. 2013;346:e7586.

55. Wilson C, Rooshenas L, Paramasivan S, Elliott D, Jepson M, Strong S, et al. Development of a framework to improve the process of recruitment to randomised controlled

For peer review only - http://bmjopen.bmj.com/site/about/guidelines.xhtml
UOB Confidential

trials (RCTs): the SEAR (Screened, Eligible, Approached, Randomised) framework. Trials. 2018;19(1):50.

56. Martin L, O'Donoghue JM, Horgan K, Thrush S, Johnson R, Gandhi A, et al. Acellular dermal matrix (ADM) assisted breast reconstruction procedures: joint guidelines from the Association of Breast Surgery and the British Association of Plastic, Reconstructive and Aesthetic Surgeons. Eur J Surg Oncol. 2013;39(5):425-9.

57. Blencowe NS, Mills N, Cook JA, Donovan JL, Rogers CA, Whiting P, et al. Standardizing and monitoring the delivery of surgical interventions in randomized clinical trials. British Journal of Surgery. 2016;103(10):1377-84.

58. Knight HJ, Musgrove JJ, Youssef MMG, Ferguson DJ, Olsen SB, Tillett RL. Significantly reducing implant loss rates in immediate implant-based breast reconstruction: A protocol and completed audit of quality assurance. J Plast Reconstr Aesthet Surg. 2020;73(6):1043-9.

59. Barr SP, Topps AR, Barnes NLP, Henderson J, Hignett S, Teasdale RL, et al. Infection prevention in breast implant surgery - A review of the surgical evidence, guidelines and a checklist. European Journal of Surgical Oncology. 2016;42:591-603.

60. Potter S, Davies C, Holcombe C, Weiler-Mithoff E, Skillman J, Vidya R, et al. International development and implementation of a core measurement set for research and audit studies in implant-based breast reconstruction: a study protocol. 2020;10(1):e035505.

61. Pusic A, Klassen A, Scott A, Klok J, ordeiro P G, Cano S. Development of a new patient-reported outcome measure for breast surgery: the BREAST-Q. Plastic and Reconstructive Surgery. 2009;124(2):345-53.

62. Herdman M, Gudex C, Lloyd A, Janssen M, Kind P, Parkin D, et al. Development and preliminary testing of the new five-level version of EQ-5D (EQ-5D-5L). Quality of life research : an international journal of quality of life aspects of treatment, care and rehabilitation. 2011;20(10):1727-36.

63. Al-Janabi H, Flynn TN, Coast J. Development of a self-report measure of capability wellbeing for adults: the ICECAP-A. Quality of life research : an international journal of quality of life aspects of treatment, care and rehabilitation. 2012;21(1):167-76.

64. Potter S, Holcombe C, Ward JA, Blazeby JM. Development of a core outcome set for research and audit studies in reconstructive breast surgery. Br J Surg. 2015;102(11):1360-71.

65. Harris PA, Taylor R, Thielke R, Payne J, Gonzalez N, Conde JG. Research electronic data capture (REDCap)--a metadata-driven methodology and workflow process for providing translational research informatics support. Journal of biomedical informatics. 2009;42(2):377-81.

66. Potter S, Davies C, Davies G, Rice C, Hollingworth W. The use of micro-costing in economic analyses of surgical interventions: a systematic review. Health Econ Rev. 2020;10(1):3.

67. Rooshenas L, Elliott D, Wade J, Jepson M, Paramasivan S, Strong S, et al. Conveying Equipoise during Recruitment for Clinical Trials: Qualitative Synthesis of Clinicians' Practices across Six Randomised Controlled Trials. PLoS Med. 2016;13(10):e1002147.

For peer review only - http://bmjopen.bmj.com/site/about/guidelines.xhtml

UOB Confidential

68. Donovan JL, de Salis I, Toerien M, Paramasivan S, Hamdy FC, Blazeby JM. The intellectual challenges and emotional consequences of equipoise contributed to the fragility of recruitment in six randomized controlled trials. J Clin Epidemiol. 2014;67(8):912-20.

For peer review only

For peer review only - http://bmjopen.bmj.com/site/about/guidelines.xhtml
UOB Confidential

Statements

Author Statement

SP conceived the study and obtained funding; KR, NM, CM, CC, WH, JT, CH, JS, KF, LW, RIC, ST, SC, PF and SP contributed to the study design; CM provided statistical support; NM and CC led the QRI; KR is the Trial Manager responsible for the day-to-day running of the trial. KR prepared the first draft of this manuscript. All authors have read and approved the final manuscript.

Funding

This work is funded by a National Institute for Health Research Clinician Scientist award to SP (CS-2016-16-019). The views expressed in this publication are those of the authors and not necessarily those of the NHS, the National Institute for Health Research or the Department of Health and Social Care.

Acknowledgements

This study is being undertaken with the support of the NIHR Biomedical Research Centre at University Hospitals Bristol and Weston NHS Foundation Trust and the University of Bristol. This study was designed and delivered in collaboration with the Bristol Trials Centre (BTC), a UKCRC Registered Clinical Trials Unit in receipt of National Institute for Health Research CTU support funding.

Trial steering and data monitoring committee members: Matthew Gardiner (chair), Dr Isabelle Smith (statistician) and Elizabeth Teasdale (PPI member).

Declaration of Interests

None

Word Count

3722/4000

For peer review only - http://bmjopen.bmj.com/site/about/guidelines.xhtml

UOB Confidential

For peer review only

Tables

Table 1: Schedule of assessment visits and outcome measurements

Timepoint	*0*	*0*	*24 hours*	*1 week*	*3 months*	*$12^{+/-10}$ months*
Activity	*Baseline*	*Day of surgery*				
Procedures						
Eligibility criteria review	●					
Informed consent	●					
Randomisation	●					
Sociodemographics	●					
Medical history	●					
Drug history	●					
Collection of routinely taken photographs	●					●
Pain Scores			●	●		
BREAST-Q	●				●	●
Operative Assessments		●				
EQ5D-5L and ICECAP-A	●				●	●
Post-operative histology MDT treatment decisions (e.g. chemotherapy and RT)					●	
Safety						
Adverse events		●			●	●

For peer review only

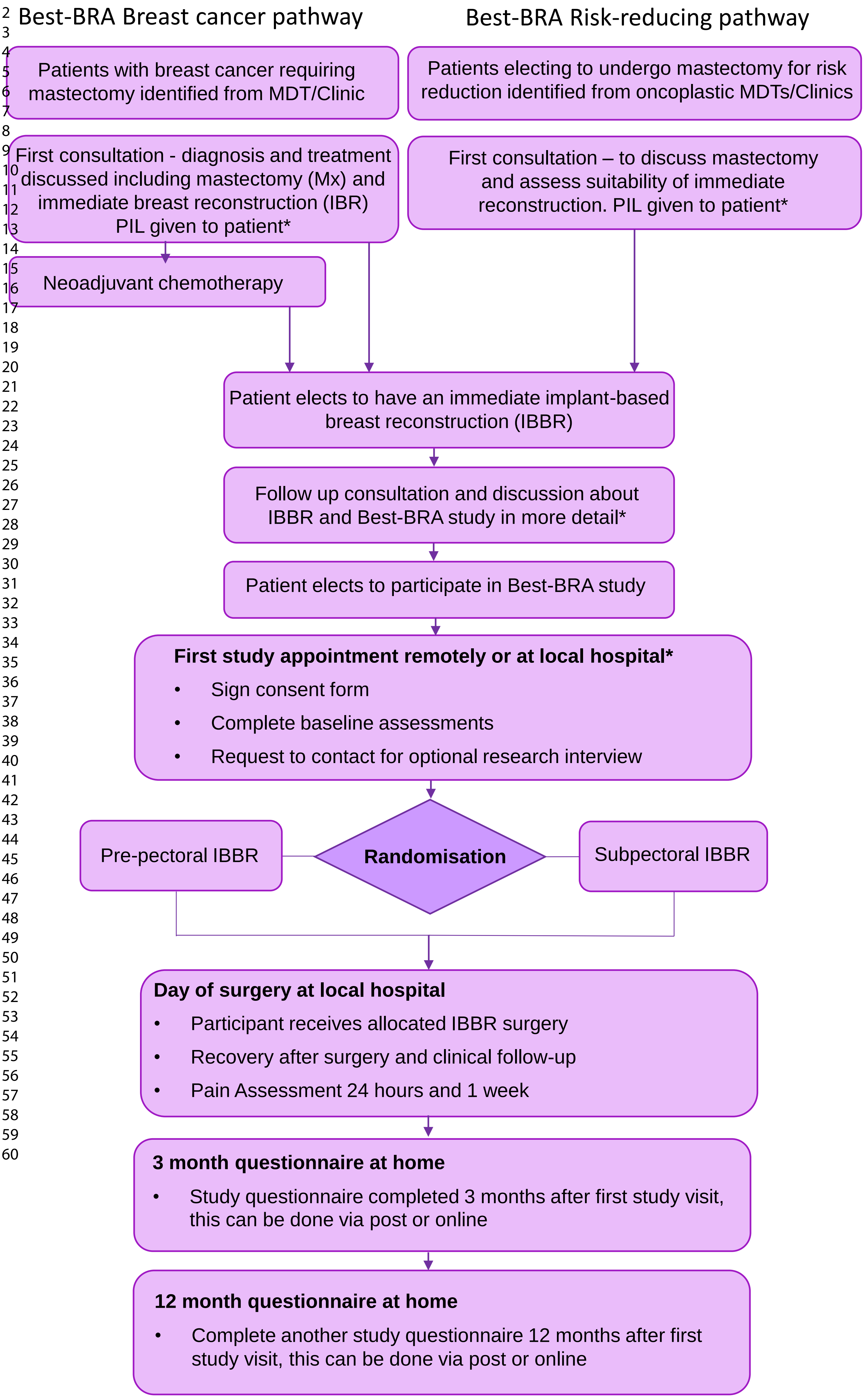

For peer review only - http://bmjopen.bmj.com/site/about/guidelines.xhtml

* These consultations will be recorded with participants' and health care professionals' consent

Appendix 1: Administrative information

Title

Best-BRA (Is subpectoral or pre-pectoral implant placement best in immediate breast reconstruction?): a protocol for a pilot randomised controlled trial of subpectoral versus pre-pectoral immediate implant-based breast reconstruction in women following mastectomy.

Trial registration number

ISRCTN: 15397185 (12 January 2021)

World Health Organization Trial Registration Data Set

Data category	Information
Primary registry and trial identifying number	ISRCTN 10081873
Date of registration in primary registry	12 January 2021
Secondary identifying numbers	IRAS: 279460<br>NHS REC: 20/WA/0338
Source(s) of monetary or material support	National Institute for Health Research (NIHR)
Primary sponsor	North Bristol NHS Trust
Secondary sponsor(s)	Not applicable
Contact for public queries	Dr Kirsty Roberts, bestbra-study@bristol.ac.uk, 07989981816
Contact for scientific queries	Ms Shelley Potter, shelley.potter@bristol.ac.uk, 0117 9287218
Public title	Best-BRA
Scientific title	Is subpectoral or pre-pectoral implant placement best in immediate breast reconstruction? The Best-BRA pilot RCT
Countries of recruitment	England, Wales, Scotland, Northern Ireland
Health condition(s) or problem(s) studied	Breast cancer
Intervention(s)	Subpectoral or pre-pectoral immediate implant-based breast reconstruction following mastectomy
Key inclusion and exclusion criteria	Inclusion: Women aged 18 or over; Require a mastectomy for breast cancer (invasive or preinvasive disease) or risk-reduction; Elect to undergo immediate IBBR; Considered eligible for either pre or subpectoral reconstruction by the surgical team.<br>Exclusion: Delayed reconstruction; Revisional surgery
Study type	Intervention
Date of first enrolment	To be determined

For peer review only

Target sample size	Pilot trial so not applicable
Recruitment status	Site set-up
Primary outcome(s)	Recruitment (number of sites recruiting; proportion of eligible women approached that are randomised, women recruited per site per month), adherence to trial allocation and outcomes completion rates will be reviewed at 12 months. At 12 months the parameters required for a fully powered trial will also be assessed (e.g number of sites and sample size).
Key secondary outcomes	Feasibility of collecting the following outcomes:<br>• The difference between groups in satisfaction with breasts using the validated BREAST-Q questionnaire at 12 months<br>• Differences between the groups in pain scores at 24 hours and 1 week<br>• The difference between groups in surgical complications, in particular implant loss, infection, re-admission and reoperation at 3 and 12 months<br>• The difference between groups in the need for additional surgery to the reconstruction or the contralateral breast at 12 months<br>• Differences between the groups in objective cosmetic outcome at 12 months assessed using patient photographs<br>• The difference between groups in their average EQ-5D-5L health related quality of life score and ICECAP-A scores at 1 year<br>• The difference between groups with respect to other key patient reported outcome domains included in the breast reconstruction core outcome set[1] including physical well-being; emotional well-being; and animation assessed using appropriate subscales of the BREAST-Q<br>• The cost effectiveness of pre-pectoral and subpectoral IBBR

Protocol version

Version 2.0 (18 October 2018)

**Version**		**Notes**
**Number**	**Date**	
2.0	05.01.21	This was the approved version by REC/HRA

Funding

NIHR

Contributorship

See main manuscript

Sponsor contact information

Trial sponsor: North Bristol NHS Trust
Sponsor's reference: 4716
Contact name: Mr Paolo Buscemi
Address: Research & Innovation, North Bristol NHS Trust, Level 3, Learning & Research building, Southmead Hospital, Westbury on Trym, Bristol, BS10 5NB
Tel: 0117 41 49345
E-mail: researchSponsor@nbt.nhs.uk

Role of study sponsor and funder

The funder and sponsor had no role in the design of this study and will not have any role during its execution, analyses, interpretation of the data, or decision to submit results.

Committees

The Trial Management Group (TMG) comprises all investigators, the trial manager, research and staff, with input from a patient/public representative. Members of the TMG will contribute to the trial in the following ways: trial design and methods; participant recruitment and trial conduct; trial management; trial logistics and cost management; economic evaluation; qualitative (QRI) study; statistical data analysis; and publication. The TMG will meet on a regular basis to oversee the management of the trial. The TMG will be provided with detailed information by the centre staff regarding trial progress. Meetings will be via videoconference facilities.

This study was designed and is being delivered in collaboration with the Bristol Trials Centre (BTC), a UKCRC registered clinical trials unit which is in receipt of National Institute for Health Research CTU support funding. Members of the BTC will attend the TMG.

Because this is a low-risk trial, the funder has agreed that the roles of both guiding the Trial Management Group and monitoring trial data will be undertaken by a single Trial Steering/Data Monitoring Committee (TS/DMC). The TS/DMC will meet at least three times over the course of the study and comprises three independent members: a chairperson, a statistician, and a patient representative. Their role will be to provide overall supervision of the trial on behalf of the funder, with a focus on progress of the trial, adherence to the protocol, patient safety and consideration of new information. The committee will review the accruing data and assess whether there are any safety issues that should be brought to the Sponsor's or the participants' attention or any reasons for the trial not to continue. Terms of reference will be drawn up and agreed with members of the TS/DMC.

For peer review only

University of BRISTOL

NHS North Bristol NHS Trust

Best-BRA Study

(Is subpectoral or pre-pectoral implant placement **Best** in immediate **BR**e**A**st reconstruction?)

Patient Information Leaflet

We are inviting you to take part in a research study

- We would like to invite you to take part in a research study. Before you decide to take part, we need you to understand why the research is being done and what it will involve for you.
- Please take time to read the following information carefully and talk to others if you wish.
- Please ask questions using the contact details if anything you read is not clear or you would like further information.

Important things that you need to know

- We are inviting women who are having a mastectomy with an immediate implant-based breast reconstruction to take part in a research study called the Best-BRA study.
- We want to find out whether placing the implant underneath the chest muscle (subpectoral) or above the muscle (pre-pectoral) is best for women who are suitable for either procedure.
- Both procedures are standard practice commonly performed across the UK; neither are new or experimental.
- In the Best-BRA Study, we want to initially find out if such a study is possible before we then do a much larger definitive study.
- Women suitable for both procedures who take part will be allocated to one of two groups: subpectoral or pre-pectoral implant-based breast reconstruction
- We will look at women's satisfaction with their implant-based reconstruction by asking them to complete three study questionnaires for up to 12 months.
- Women who take part can leave the study at any time and will have no impact on the care they receive.
- In this research study we will use information from you and your medical records. We will only use information that we need for the research study.
- We will let very few people know your name or contact details, and only if they really need it for this study.

For peer review only - http://bmjopen.bmj.com/site/about/guidelines.xhtml

University of BRISTOL · independent cancer patients' voice · NIHR National Institute for Health Research · BRISTOL TRIALS CENTRE

- Everyone involved in this study will keep your data safe and secure. We will also follow all privacy rules.
- At the end of the study we will save some of the clinical data in case we need to check it and for future research.
- We will make sure no-one can work out who you are from the reports we write.
- ***If you would like more information on what is involved, please turn over and read Section A***

Contact details:
Name: <INSERT Name of Local Research Nurse>,
Address: <Insert Local hospital address>
Tel: <INSERT Local hospital phone number>
Email: bestbra-study@bristol.ac.uk

For peer review only

For peer review only - http://bmjopen.bmj.com/site/about/guidelines.xhtml

University of BRISTOL

NHS North Bristol NHS Trust

PART A: Why is this study being done and what will happen if you take part?

What is the purpose of the study?

Breast cancer affects one in eight women in the UK and 40% of all women with breast cancer have a mastectomy (removal of the breast) as their surgical treatment. Losing a breast may affect women's self-esteem and body image and breast reconstruction is offered to improve their quality of life.

Breast reconstruction using implants is the most commonly performed procedure in the UK. There are two ways in which implants can be placed during implant-based breast reconstruction; the implants can be placed under the chest muscle (a subpectoral implant) or placed on top of the chest muscle (pre-pectoral implant). Both are standard procedures commonly performed across the UK.

In the Best-BRA study, we want to find out if surgeons and women would be willing to take part in a study to find out if putting the implant on top of the muscle or underneath it improves women's satisfaction with the outcome of their reconstruction. There is currently no good evidence which implant procedure is best for patients (see below).

**Subpectoral reconstruction**	**Pre-pectoral reconstruction**
Implant placed under the chest (pectoral) muscle, implant may or may not be partially covered in mesh	Implant placed on top of the chest (pectoral) muscle, implant usually covered in mesh
Will involve 1-2 nights in hospital	May involve 1 night in hospital or you may be allowed home on the same day
Your surgeon is likely to place 1 or more drains at the time of surgery	Your surgeon is likely to place 1 or more drains at the time of surgery
The implant may move when the chest muscle contracts (this is called animation)	As the implant is on top of the muscle it does not move as much when the chest wall muscle contracts
The muscle covers the implant so the edge of the implant and any ripples on the surface are less easy to see.	The edges of the implant and ripples on the implant surface may be easier to see
May require further surgery to improve the appearance of the reconstruction over time	May be require further surgery to the reconstruction (such as fat transfer) to hide the implant over time

We also want to capture how information about the study is conveyed to patients. This will help us understand how research studies are explained to people and if there are any improvements we can make.

Why have I been invited?

Your surgeon is working with the research team to understand more about which implant-based technique is best. You have been asked to take part in this study because you are having a mastectomy and are suitable for an immediate implant-based breast reconstruction.

We hope to recruit women having immediate implant-based breast reconstruction from UK hospitals.

Do I have to take part?

No. It is your choice whether or not to take part in the Best-BRA study. If you decide to not take part, the treatment and care you receive from your doctors will not be affected in any way. If you do decide to take part, you are also free to leave the study at any time you wish, without giving a reason.

What is involved in the study?

If you take part in the Best-BRA study, following your mastectomy, you will have an immediate implant-based breast reconstruction where either the implant will be placed on top of the chest (pre-pectoral) muscle or below it (subpectoral), both of which are standard procedures. As we do not know which is best for women, the type of surgery you will have will be allocated through a process called

University of BRISTOL independent cancer patients' voice www.independentcancerpatientsvoice.org.uk NIHR National Institute for Health Research BRISTOL TRIALS CENTRE

For peer review only - http://bmjopen.bmj.com/site/about/guidelines.xhtml

randomisation. This means you will have an equal chance of having a pre-pectoral implant-based breast reconstruction procedure or a subpectoral implant-based breast reconstruction procedure. Randomisation is used as it creates groups of patients that are similar except for whether the implant is placed under or on top of the muscle. This will enable a fair comparison of the different techniques so we can assess at the end of the study which technique is best. Neither you, the surgeons, or the research team can choose which group you go in, as this could result in the groups being unequal and the findings unreliable. It is important that you only agree to take part if you are prepared to accept either of the two implant-based breast reconstruction procedures.

With your consent, we plan to record conversations where the Best-BRA study is discussed with you. We would like to see how the study is explained to you and if we can improve this. This is optional. As part of this, we may also invite some women to have an optional discussion (research 'interview') with a researcher to explore their views on the study and whether they chose to take part or not. This may take up to an hour, it will be over the telephone and will be recorded with consent.

If I decide to take part, what happens next?

The diagram on page 5 illustrates the flow of the study, which we detail here;

A) First discussion about the Best-BRA study

You will have an appointment with your surgeon and breast care nurse to discuss your surgery and the study in more detail. Following this discussion, a member of the local research team will meet with you at one of your routine clinical appointments, or telephone you, to discuss the study in more detail, answer any questions you may have and arrange your first study visit at your local hospital (see below).

With your consent we would like to record this discussion and any future discussions you have about the study, regardless of whether you decide to take part in the main study or not.

B) First study appointment at your local hospital

This appointment will be at your local hospital if it coincides with one of your routine appointments, otherwise it will be carried out over the phone or using video conferencing if you are happy to do this. At this appointment, a member of the local research team will confirm that you are still happy to take part.

You will be asked to:

- **Sign 2 consent forms** confirming that you agree to the recording of discussions (unless agreed earlier) and that you agree to take part in the study. The consent forms can be completed online or over the phone and they can be posted to you if you are not at the hospital;
- **Complete a study questionnaire** about your general health, how you feel about your body, your experiences and your quality of life before the surgery. The questionnaire should take no more than 20 minutes to complete.
- **Provide some additional information** for the purposes of the study, e.g. contact details, GP information, date of birth.
- If you are having **pre and post-operative photographs** taken as part of your care, you will be asked at this appointment if you are happy for the surgeon or breast care nurse to share these with the research team and they will be reviewed as part of the study. This is optional.

You will then be informed whether you will be in the "Pre-pectoral implant group" or the "Subpectoral implant Group".

We will inform your GP of your participation in the study and let them know which group you have been allocated to.

For peer review only - http://bmjopen.bmj.com/site/about/guidelines.xhtml

University of BRISTOL

NHS North Bristol NHS Trust

Your first consultation to discuss treatment options
Study will be introduced briefly*

- Read Patient Information Leaflet
- Ask any questions

A) First discussion about the Best-BRA study if you decide to have implant-based reconstruction*

At routine clinical appointment or via telephone

Research nurse will arrange your first study appointment

B) First study appointment remotely or at your local hospital*

- Discuss study and ask questions
- Sign consent form
- Request to contact for optional research interview
- Complete study questionnaires
- Allocation to study group

Pre-pectoral implant-based breast reconstruction **OR** **Subpectoral** implant-based breast reconstruction

C) Surgery at your local hospital

- Preparation for surgery by clinical team- this will include a pre-operative assessment clinic
- You will receive your allocated implant-based breast reconstruction surgery
- Recovery after surgery and clinical follow-up
- Pain Assessment 24 hours and 1 week

D) 3 month questionnaire at home

- Complete study questionnaire 3 months after your first study visit, this can be done via post or online

E) 12 month questionnaire at home

- Complete another study questionnaire 12 months after your first study visit, this can be done via post or online

* These consultations with your consent will be audio recorded

For peer review only - http://bmjopen.bmj.com/site/about/guidelines.xhtml

University of BRISTOL independent cancer patients' voice www.independentcancerpatientsvoice.org.uk NIHR National Institute for Health Research BRISTOL TRIALS CENTRE

University of BRISTOL

North Bristol NHS Trust

C) The day of your surgery

You will be invited to attend your local hospital to **have your surgery** (a mastectomy followed by either pre-pectoral or subpectoral implant-based breast reconstruction). The clinical team will prepare you for your surgery as they would during normal NHS care. A member of the research team will also record some other information from your hospital notes about your planned surgery.

The surgeons who will carry out your breast reconstruction in this study perform them regularly. You will receive the usual NHS treatment and aftercare at your hospital (e.g. medical tests, procedures, and follow-up appointments). If you have any doubts, concerns or questions about your operation you should talk to your consultant surgeon, before agreeing to the surgery or to take part in the research.

Please note: you may need to attend a pre-operative assessment clinic before your surgery. Your clinical team will advise you on local procedures.

24 hours and 1 week following your surgery we will ask you about any pain you are experiencing following the surgery. This will be a single question asking you to record this using a scale which can be completed online or with help from the research team.

D) Questionnaire to complete at home: 3 months after your first study visit

You will be asked to **complete a study questionnaire** about your general health, how you feel about your body, your experiences and your quality of life after the surgery. The questionnaire should take no longer than 20 minutes to complete and can be done by post (we will provide a pre-paid envelope) or online.

E) Questionnaire to complete at home: 12 months after your first study visit

12 months after your implant-based breast reconstruction, you will be asked to **complete another study questionnaire** about your general health, how you feel about your body, your experiences and your quality of life after the surgery.

What are the possible benefits of taking part?

This study will not benefit you directly but the information that you provide will help to improve the future management of patients who undergo implant breast reconstructive surgery. Also, some people enjoy being part of a research study because of the close contact with research staff and their opportunity to share their opinions and experiences of their condition and treatments.

What are the possible disadvantages and risks of taking part?

There should be no additional risks to routine NHS practice of either implant-based reconstruction procedure, and neither are new or experimental. You will have the same risks as anyone having immediate implant-based reconstruction. Your doctor will explain the risks and benefit of each procedure, and they will provide relevant hospital leaflets. The questionnaires will take approximately 20 minutes of your time.

What happens after the study is over?

After completing your 12 months questionnaire, you will return to standard NHS care. However, we would like to be able to contact you after this, with your permission. We would like to contact you again to a) check on your long-term health, for example by sending you other questionnaires to add information to what we already know about you, or by checking NHS medical records; and b) to ask you if you would like to take part in other relevant studies. You will not have to reply to any questionnaires or take part in other studies unless you want to at that time.

What if I change my mind and I don't want to carry on with the study?

For peer review only - http://bmjopen.bmj.com/site/about/guidelines.xhtml

University of BRISTOL independent cancer patients' voice NIHR National Institute for Health Research BRISTOL TRIALS CENTRE

You are free to withdraw from the study at any point without giving reason. Your medical treatment will not be affected. If you wish to leave the study, please speak to your doctor or research nurse. If you decide you no longer wish to take part, any information we have collected up until the point you leave will be retained and used in the analysis of the trial results.

What about expenses and travel?

We will provide you with prepaid envelopes to return the study questionnaires if you opt to not complete them online or by telephone. We can offer to cover some travel expenses for study specific hospital visit(s).

What should I do now?

After reading this information sheet, we hope that you will be interested in taking part. You can contact the research nurse if you have any questions. If you would like to take part please also read section B of this information sheet.

PART B: Further general information about the study and what will happen to your data if you decide to take part

Will the information I provide be kept confidential?

Yes. We will follow ethical and legal practice and all information about you will be handled in confidence.

Any information that is collected about you during the study will be kept strictly confidential. All information collected for the study at any time will be stored using a 'study identity number' for confidentiality and will be kept secure using passwords on a University of Bristol server. The information will be handled in line with data protection requirements and will only be available to those responsible for maintaining research standards. We will need to use information from you and/or from your medical records for this research project. This information will include your:

- Initials
- NHS number
- Name
- Gender
- Date of birth
- Contact details (for example: postcode, telephone number, email address)

Your data will be stored and used in compliance with the relevant, current data protection laws; Data Protection Act 2018 and General Data Protection Regulation (GDPR). North Bristol NHS Trust is the sponsor for this study based in the United Kingdom. We will be using information from you and/or your medical records in order to undertake this study and North Bristol NHS Trust and the Bristol Trials Centre (BTC, University of Bristol) will act as joint data controllers for this study. This means that we are responsible for looking after your information safely and using it properly. The BTC at the University of Bristol, who manage the study on behalf of North Bristol NHS Trust, will keep identifiable information about you for 5 years after the study has finished, which is considered good practice for clinical trials. After that period, the data will be securely destroyed (except audio-recordings which will be retained for future studies with your consent).

Your rights to access, change or move your information are limited, as we need to manage your information in specific ways in order for the research to be reliable and accurate. If you withdraw from the study, we will keep the information about you that we have already obtained. To safeguard your rights, we will use the minimum personally identifiable information possible.

Other researchers may wish to access anonymised data from this study in the future. If you take part in the Best-BRA study, anonymous data collected in this study may be used in future studies with the necessary approval. This will not include names,

For peer review only - http://bmjopen.bmj.com/site/about/guidelines.xhtml

University of BRISTOL

NHS North Bristol NHS Trust

addresses or dates of birth, and it will not be possible to identify you.

If you consent to recording of discussions with study staff and/or a research interview, then all recorded data will be handled in the strictest confidence. Data will be captured on an encrypted audio-recorder or using the video conferencing facilities, transferred securely to the Best-BRA study team at the University of Bristol, and held on a password protected computer. If video conferencing is used, only the audio part of the recording will be retained. Files will be labelled with a reference number (as opposed to your name or any personal information). Recordings will be transcribed (i.e. written word for word) by University of Bristol employees or their authorised representatives. Transcripts (i.e. a written account of what was said) will be anonymised so that you cannot be identified. Data will be analysed and used for training, teaching, research and publication purposes in Best-BRA and future studies. Any quotes used will be anonymised and extracts of recordings will be voice-modified so that you cannot be identified.

Anonymised transcripts of recordings may be made available to other researchers outside of the Best-BRA study via controlled access if they secure the necessary approvals. Data from the anonymised transcripts may be used for purposes not related to this study, but it will not be possible to identify you from them.

You may change your mind about having these consultations or the research interview recorded at any time without it affecting your legal rights or medical care, although any recordings collected before your withdrawal may be retained and used anonymously in a way that will not identify you. Agreeing to record your consultations or taking part in a research interview does not commit you to taking part in the Best-BRA research study.

You can find out more about how we use your information at:

- www.hra.nhs.uk/information-about-patients/
- Our leaflet available from www.nbt.nhs.uk/PatientResearchdata
- By asking one of the research team at bestbra-study@bristol.ac.uk.
- By contacting Helen Williamson (Head of Information Governance) at helen.e.williamson@nbt.nhs.uk or by ringing 0117 41 44767.

Your hospital will collect information from you and/or your medical records for this research study in accordance with our instructions.

Your hospital will use your name, NHS number and contact details to contact you about the research study, and make sure that relevant information about the study is recorded for your care, and to oversee the quality of the study. Individuals from North Bristol NHS Trust, the BTC and regulatory organisations may look at your medical and research records to check the accuracy of the research study. Your hospital will pass these details to the BTC along with the information collected from you and/or your medical records. The only people in North Bristol NHS Trust and the BTC who will have access to information that identifies you will be people who need to contact you to audit the data collection process. The people who analyse the information will not be able to identify you and will not be able to find out your name, NHS number or contact details.

Your hospital will keep identifiable information about you from this study for 5 years after the study has finished (this does not include the audio-recordings).

What will happen to the results of the research study?

The results will be published in medical journals, presented at conferences with other healthcare professionals and specialists, reported on open access databases and open platform research registries and made publicly available. A short version will also be available to you. No one will be able to identify you from any of the study reports.

For peer review only - http://bmjopen.bmj.com/site/about/guidelines.xhtml

Who is organising or sponsoring the research?

The study is being funded by the National Institute of Health Research (NIHR) (reference 2016-16-019) The research is being carried out by a group of experienced doctors and researchers at each of the hospitals involved in the study and the University of Bristol. This study is sponsored by North Bristol NHS Trust, and the Bristol Trials Centre are responsible for managing the study. The study is being conducted by Miss Shelley Potter, Consultant Senior Lecturer at the Centre for Surgical Research University of Bristol.

Who has reviewed the study?

This study has been reviewed by the Health Research Authority and NHS Research Ethics Committee <INSERT Name of REC Committee> who have provided approval for this study to be conducted in the NHS.

What if I have any concerns?

If you have a concern regarding your care as a patient, please discuss this with your surgeon or nurse. If you become unable or unwilling to continue taking part in the Best-BRA Study, we would withdraw you from it. Your medical treatment will continue as usual with your hospital team and GP.

In the unlikely event that something does go wrong, and you are harmed during the research study there are no special compensation arrangements. If you are harmed and this is due to someone's negligence, then you may have grounds for a legal action for compensation against North Bristol NHS Trust, but you may have to pay your legal costs. The normal National Health Service complaints mechanisms will still be available to you.

If you have any questions about the study, or any aspect of your treatment or health whilst on the study, please ask to speak to your Best-BRA study research nurse or surgeon <INSERT name, address, email address, telephone numbers including the 24 hour emergency contact number>.

Alternatively, you can contact the Trial Manager (email: bestbra-study@bristol.ac.uk; phone: 0117 9287351).

The Patient Advice and Liaison Service (PALS) /Advice & Complaints Team (ACT) [delete as appropriate] should be contacted for any complaints. Your local PALS/ACT [delete as appropriate] is:

<INSERT local details>

THANK YOU FOR TAKING THE TIME TO READ THIS INFORMATION.
PLEASE KEEP A COPY FOR YOUR RECORDS.

Funding Acknowledgement: *This project is funded by the National Institute for Health Research (NIHR) (project reference CS-2016-16-019). The views expressed are those of the author(s) and not necessarily those of the NIHR or the Department of health and Social Care.*

BTC Acknowledgement: *This study was designed and delivered in collaboration with the Bristol Trials Centre (BTC), a UKCRC registered clinical trials unit which, as part of the Bristol Trials Centre, is in receipt of National Institute for Health Research CTU support funding.*

For peer review only

For peer review only - http://bmjopen.bmj.com/site/about/guidelines.xhtml

University of BRISTOL | FUNDED BY NIHR National Institute for Health Research | BRISTOL TRIALS CENTRE | NHS North Bristol NHS Trust

Best-BRA Study
(Is subpectoral or pre-pectoral implant placement **Best** in immediate **BR**e**A**st reconstruction?)

Participant Consent Form

Participant ID:						

Please initial Relevant boxes

1. I confirm that I have read the patient Information leaflet <INSERT version & date> for the above study. I have had the opportunity to consider the information, ask questions and have had these answered satisfactorily. ☐

2. I understand that my participation is voluntary and that I am free to withdraw at any time without giving any reason, without my medical care or legal rights being affected. I understand that all information collected up until the point I withdraw will be retained for analysis. ☐

3. I understand that after the study ends, the research data (results) collected may be made "open data". This means the data will be publicly available and may be used for purposes not related to this study. However, any personal information that could identify me will be removed or changed before files are shared with other researchers or results are made public. This does not apply to QRI data that is 'controlled access' (see separate consent form regarding the audio-recording of consultations). ☐

4. I understand that relevant sections of my medical notes and data collected during the study, including personal identifiable data may be looked at by individuals from regulatory authorities, the University of Bristol study research team or from the NHS Trust, to ensure that the research is conducted appropriately. I give permission for these individuals to have access to my records. ☐

5. I agree that my GP will be told that I am taking part in this research study. ☐

6. I give consent for the data collected in this trial to be used in future ethically approved studies on the understanding that all information will continue to be kept securely and remain confidential. ☐

Please continue on next page

For peer review only - http://bmjopen.bmj.com/site/about/guidelines.xhtml

University of BRISTOL | FUNDED BY NIHR National Institute for Health Research | BRISTOL TRIALS CENTRE | NHS North Bristol NHS Trust

7. *OPTIONAL: I agree for my pre-operative and post-operative photographs if they have been taken, to be shared securely with the research team and to be analysed as part of the study* ☐

8. *OPTIONAL: I agree to being contacted in the future by the research team to provide information about my long-term health status. I understand I can then decide to not take part in any future studies if I change my mind.* ☐

7. I agree to take part in the above Best-BRA Study. ☐

______________________	______________________	______________________
**Name of patient**	**Signature**	**Date**
______________________	______________________	______________________
**Name of person taking consent**<br>You must have signed the<br>Site Signature & Delegation Log	**Signature**	**Date**

Original to be kept in the Investigator Site File, 1 copy in hospital notes, 1 copy to the patient, 1 copy to the Trial Office

For peer review only

Funding Acknowledgement: *This project is funded by the National Institute for Health Research (NIHR) HTA programme (project reference 17/95/03). The views expressed are those of the author(s) and not necessarily those of the NIHR or the Department of health and Social Care.*

BRTC Acknowledgement: *This study was designed and delivered in collaboration with the Bristol Randomised Trials Collaboration (BRTC), a UKCRC registered clinical trials unit which, as part of the Bristol Trials Centre, is in receipt of National Institute for Health Research CTU support funding.*

For peer review only - http://bmjopen.bmj.com/site/about/guidelines.xhtml

SPIRIT

STANDARD PROTOCOL ITEMS: RECOMMENDATIONS FOR INTERVENTIONAL TRIALS

SPIRIT 2013 Checklist: Recommended items to address in a clinical trial protocol and related documents*

Section/item	Item No	Description	Location
**Administrative information**			
Title	1	Descriptive title identifying the study design, population, interventions, and, if applicable, trial acronym	Page 1
Trial registration	2a	Trial identifier and registry name. If not yet registered, name of intended registry	Page 4
	2b	All items from the World Health Organization Trial Registration Data Set	Appendix 1
Protocol version	3	Date and version identifier	Appendix 1
Funding	4	Sources and types of financial, material, and other support	Page 25
Roles and responsibilities	5a	Names, affiliations, and roles of protocol contributors	Page 25
	5b	Name and contact information for the trial sponsor	Appendix 1
	5c	Role of study sponsor and funders, if any, in study design; collection, management, analysis, and interpretation of data; writing of the report; and the decision to submit the report for publication, including whether they will have ultimate authority over any of these activities	Page 25/Appendix 1
	5d	Composition, roles, and responsibilities of the coordinating centre, steering committee, endpoint adjudication committee, data management team, and other individuals or groups overseeing the trial, if applicable (see Item 21a for data monitoring committee)	Page 16
**Introduction**			

For peer review only

Background and rationale	6a	Description of research question and justification for undertaking the trial, including summary of relevant studies (published and unpublished) examining benefits and harms for each intervention	Page 5
	6b	Explanation for choice of comparators	Page 6/7
Objectives	7	Specific objectives or hypotheses	Page 7
Trial design	8	Description of trial design including type of trial (eg, parallel group, crossover, factorial, single group), allocation ratio, and framework (eg, superiority, equivalence, noninferiority, exploratory)	Page 8
**Methods: Participants, interventions, and outcomes**			
Study setting	9	Description of study settings (eg, community clinic, academic hospital) and list of countries where data will be collected. Reference to where list of study sites can be obtained	Page 8
Eligibility criteria	10	Inclusion and exclusion criteria for participants. If applicable, eligibility criteria for study centres and individuals who will perform the interventions (eg, surgeons, psychotherapists)	Page 9,10
Interventions	11a	Interventions for each group with sufficient detail to allow replication, including how and when they will be administered	Pages 9-10
	11b	Criteria for discontinuing or modifying allocated interventions for a given trial participant (eg, drug dose change in response to harms, participant request, or improving/worsening disease)	N/A
	11c	Strategies to improve adherence to intervention protocols, and any procedures for monitoring adherence (eg, drug tablet return, laboratory tests)	Page 16
	11d	Relevant concomitant care and interventions that are permitted or prohibited during the trial	Pages 9-11

For peer review only - http://bmjopen.bmj.com/site/about/guidelines.xhtml

Outcomes	12	Primary, secondary, and other outcomes, including the specific measurement variable (eg, systolic blood pressure), analysis metric (eg, change from baseline, final value, time to event), method of aggregation (eg, median, proportion), and time point for each outcome. Explanation of the clinical relevance of chosen efficacy and harm outcomes is strongly recommended	Pages 11-12, Table 1
Participant timeline	13	Time schedule of enrolment, interventions (including any run-ins and washouts), assessments, and visits for participants. A schematic diagram is highly recommended (see Figure)	Pages 8-10, Figure 1 and Table 1
Sample size	14	Estimated number of participants needed to achieve study objectives and how it was determined, including clinical and statistical assumptions supporting any sample size calculations	Page 11 & 13
Recruitment	15	Strategies for achieving adequate participant enrolment to reach target sample size	Pages 8 & 14-16 (QRI)
**Methods: Assignment of interventions (for controlled trials)**			
Allocation:			
Sequence generation	16a	Method of generating the allocation sequence (eg, computer-generated random numbers), and list of any factors for stratification. To reduce predictability of a random sequence, details of any planned restriction (eg, blocking) should be provided in a separate document that is unavailable to those who enrol participants or assign interventions	Page 10
Allocation concealment mechanism	16b	Mechanism of implementing the allocation sequence (eg, central telephone; sequentially numbered, opaque, sealed envelopes), describing any steps to conceal the sequence until interventions are assigned	Page 10
Implementation	16c	Who will generate the allocation sequence, who will enrol participants, and who will assign participants to interventions	Page 10
Blinding (masking)	17a	Who will be blinded after assignment to interventions (eg, trial participants, care providers, outcome assessors, data analysts), and how	n/a

For peer review only - http://bmjopen.bmj.com/site/about/guidelines.xhtml

	17b	If blinded, circumstances under which unblinding is permissible, and procedure for revealing a participant's allocated intervention during the trial	n/a
**Methods: Data collection, management, and analysis**			
Data collection methods	18a	Plans for assessment and collection of outcome, baseline, and other trial data, including any related processes to promote data quality (eg, duplicate measurements, training of assessors) and a description of study instruments (eg, questionnaires, laboratory tests) along with their reliability and validity, if known. Reference to where data collection forms can be found, if not in the protocol	Page 12-13 & 16 and Table 1
	18b	Plans to promote participant retention and complete follow-up, including list of any outcome data to be collected for participants who discontinue or deviate from intervention protocols	Page 13
Data management	19	Plans for data entry, coding, security, and storage, including any related processes to promote data quality (eg, double data entry; range checks for data values). Reference to where details of data management procedures can be found, if not in the protocol	Pages 15
Statistical methods	20a	Statistical methods for analysing primary and secondary outcomes. Reference to where other details of the statistical analysis plan can be found, if not in the protocol	Page 13
	20b	Methods for any additional analyses (eg, subgroup and adjusted analyses)	N/A
	20c	Definition of analysis population relating to protocol non-adherence (eg, as randomised analysis), and any statistical methods to handle missing data (eg, multiple imputation)	N/A
**Methods: Monitoring**			
Data monitoring	21a	Composition of data monitoring committee (DMC); summary of its role and reporting structure; statement of whether it is independent from the sponsor and competing interests; and reference to where further details about its charter can be found, if not in the protocol. Alternatively, an explanation of why a DMC is not needed	Page 16

	21b	Description of any interim analyses and stopping guidelines, including who will have access to these interim results and make the final decision to terminate the trial	n/a
Harms	22	Plans for collecting, assessing, reporting, and managing solicited and spontaneously reported adverse events and other unintended effects of trial interventions or trial conduct	Pages 16-17
Auditing	23	Frequency and procedures for auditing trial conduct, if any, and whether the process will be independent from investigators and the sponsor	Page 16
**Ethics and dissemination**			
Research ethics approval	24	Plans for seeking research ethics committee/institutional review board (REC/IRB) approval	Page 4 % 18
Protocol amendments	25	Plans for communicating important protocol modifications (eg, changes to eligibility criteria, outcomes, analyses) to relevant parties (eg, investigators, REC/IRBs, trial participants, trial registries, journals, regulators)	Page 17
Consent or assent	26a	Who will obtain informed consent or assent from potential trial participants or authorised surrogates, and how (see Item 32)	Pages 8-9,
	26b	Additional consent provisions for collection and use of participant data and biological specimens in ancillary studies, if applicable	N/A
Confidentiality	27	How personal information about potential and enrolled participants will be collected, shared, and maintained in order to protect confidentiality before, during, and after the trial	Pages 16
Declaration of interests	28	Financial and other competing interests for principal investigators for the overall trial and each study site	Page 25
Access to data	29	Statement of who will have access to the final trial dataset, and disclosure of contractual agreements that limit such access for investigators	Pages 16
Ancillary and post-trial care	30	Provisions, if any, for ancillary and post-trial care, and for compensation to those who suffer harm from trial participation	Page 10- 11

For peer review only

Dissemination policy	31a	Plans for investigators and sponsor to communicate trial results to participants, healthcare professionals, the public, and other relevant groups (eg, via publication, reporting in results databases, or other data sharing arrangements), including any publication restrictions	Page 18
	31b	Authorship eligibility guidelines and any intended use of professional writers	N/A
	31c	Plans, if any, for granting public access to the full protocol, participant-level dataset, and statistical code	Page 16
**Appendices**			
Informed consent materials	32	Model consent form and other related documentation given to participants and authorised surrogates	Appendix 2 & Appendix 3
Biological specimens	33	Plans for collection, laboratory evaluation, and storage of biological specimens for genetic or molecular analysis in the current trial and for future use in ancillary studies, if applicable	N/A

*It is strongly recommended that this checklist be read in conjunction with the SPIRIT 2013 Explanation & Elaboration for important clarification on the items. Amendments to the protocol should be tracked and dated. The SPIRIT checklist is copyrighted by the SPIRIT Group under the Creative Commons "Attribution-NonCommercial-NoDerivs 3.0 Unported" license.

For peer review only

BMJ Open

Best-BRA (Is subpectoral or pre-pectoral implant placement best in immediate breast reconstruction?) A protocol for a pilot randomised controlled trial of subpectoral versus pre-pectoral immediate implant-based breast reconstruction in women following mastectomy.

Journal:	*BMJ Open*
Manuscript ID	bmjopen-2021-050886.R1
Article Type:	Protocol
Date Submitted by the Author:	18-Aug-2021
Complete List of Authors:	Roberts, Kirsty; University of Bristol Medical School, Population Health Sciences<br>Mills, Nicola; University of Bristol Medical School, Population Health Sciences<br>Metcalfe, Chris; University of Bristol Medical School, Population Health Sciences<br>Lane, Athene; University of Bristol Medical School, Population Health Sciences<br>Clement, Clare; University of Bristol Medical School, Population Health Sciences<br>Hollingworth, William; University of Bristol Medical School, Population Health Sciences<br>Taylor, Jodi; University of Bristol Medical School, Population Health Sciences<br>Holcombe, Chris; Royal Liverpool and Broadgreen University Hospitals NHS Trust, Linda McCartney Centre<br>Skillman, Joanna; University Hospitals Coventry and Warwickshire NHS Trust, Department of Plastic Surgery<br>Fairhurst, Katherine; University of Bristol Medical School, Population Health Sciences<br>Whisker, Lisa; Nottingham University Hospitals NHS Trust, Nottingham Breast Institute<br>Cutress, RI ; University of Southampton<br>Thrush, Steven; Worcestershire Acute Hospitals NHS Trust, Breast Unit<br>Fairbrother, Patricia; Independent Cancer Patients' Voice<br>Potter, Shelley; University of Bristol Medical School, Population Health Sciences; North Bristol NHS Trust, Bristol Breast Care Centre
**Primary Subject Heading**:	Surgery
Secondary Subject Heading:	Oncology
Keywords:	Breast surgery < SURGERY, Breast tumours < ONCOLOGY, PLASTIC & RECONSTRUCTIVE SURGERY, QUALITATIVE RESEARCH

For peer review only - http://bmjopen.bmj.com/site/about/guidelines.xhtml

I, the Submitting Author has the right to grant and does grant on behalf of all authors of the Work (as defined in the below author licence), an exclusive licence and/or a non-exclusive licence for contributions from authors who are: i) UK Crown employees; ii) where BMJ has agreed a CC-BY licence shall apply, and/or iii) in accordance with the terms applicable for US Federal Government officers or employees acting as part of their official duties; on a worldwide, perpetual, irrevocable, royalty-free basis to BMJ Publishing Group Ltd ("BMJ") its licensees and where the relevant Journal is co-owned by BMJ to the co-owners of the Journal, to publish the Work in this journal and any other BMJ products and to exploit all rights, as set out in our licence.

The Submitting Author accepts and understands that any supply made under these terms is made by BMJ to the Submitting Author unless you are acting as an employee on behalf of your employer or a postgraduate student of an affiliated institution which is paying any applicable article publishing charge ("APC") for Open Access articles. Where the Submitting Author wishes to make the Work available on an Open Access basis (and intends to pay the relevant APC), the terms of reuse of such Open Access shall be governed by a Creative Commons licence – details of these licences and which Creative Commons licence will apply to this Work are set out in our licence referred to above.

Other than as permitted in any relevant BMJ Author's Self Archiving Policies, I confirm this Work has not been accepted for publication elsewhere, is not being considered for publication elsewhere and does not duplicate material already published. I confirm all authors consent to publication of this Work and authorise the granting of this licence.

For peer review only

Best-BRA (Is subpectoral or pre-pectoral implant placement best in immediate breast reconstruction?) A protocol for a pilot randomised controlled trial of subpectoral versus pre-pectoral immediate implant-based breast reconstruction in women following mastectomy.

Kirsty Roberts[1], Nicola Mills[1], Chris Metcalfe[1], Athene Lane[1], Clare Clement[1], William Hollingworth[1], Jodi Taylor[1], Chris Holcombe[2], Jo Skillman[3], Katherine Fairhurst[4], Lisa Whisker[5], Ramsey I Cutress[6], Steven Thrush[7], Patricia Fairbrother[8], Shelley Potter[4,9].

[1]Population Health Sciences, Bristol Medical School, University of Bristol, Bristol, UK,

[2]Linda McCartney Centre, Royal Liverpool and Broadgreen University Hospital, Liverpool, UK,

[3] University Hospitals Coventry and Warwickshire NHS Trust, Coventry, UK,

[4] Bristol Centre for Surgical Research, Population Health Sciences, Bristol Medical School, University of Bristol, Bristol, UK,

[5] Nottingham Breast Institute, Nottingham University Hospitals NHS Trust, Nottingham, UK,

[6] University of Southampton, University Hospitals Southampton, Southampton, UK

[7]Breast Unit, Worcester Royal Hospital, Worcester, UK.

[8] Independent Cancer Patients' Voice, London, UK,

[9] Bristol Breast Care Centre, North Bristol NHS Trust, Bristol, UK.

Corresponding author

Shelley Potter, Associate Professor of Oncoplastic Breast Surgery

For peer review only - http://bmjopen.bmj.com/site/about/guidelines.xhtml

UOB Confidential

Postal address: Bristol Centre for Surgical Research, Population Health Sciences, Bristol Medical School, University of Bristol, Canynge Hall, 39 Whatley Road, Bristol BS8 2PS

Email: shelley.potter@bristol.ac.uk

Telephone: 0117 9287218

Keywords

Implant-based breast reconstruction, pre-pectoral, subpectoral, pilot trial, randomised controlled trial, QuinteT Recruitment Intervention

For peer review only

For peer review only - http://bmjopen.bmj.com/site/about/guidelines.xhtml

UOB Confidential

Abstract

Background

Implant-based breast reconstruction (IBBR) is the most commonly performed reconstructive procedure following mastectomy. IBBR techniques are evolving rapidly, with mesh-assisted subpectoral reconstruction becoming the standard of care and more recently, pre-pectoral techniques being introduced. These muscle-sparing techniques may reduce post-operative pain, avoid implant animation and improve cosmetic outcomes and have been widely adopted into practice. Although small observational studies have failed to demonstrate any differences in the clinical or patient-reported outcomes of pre or subpectoral reconstruction, high-quality comparative evidence of clinical or cost-effectiveness is lacking. A well-designed, adequately powered randomised controlled trial (RCT) is needed to compare the techniques, but breast reconstruction RCTs are challenging. We, therefore, aim to undertake an external pilot RCT (Best-BRA) with an embedded QuinteT Recruitment Intervention (QRI) to determine the feasibility of undertaking a trial comparing pre and subpectoral techniques.

Methods and analysis

Best-BRA is a pragmatic, two-arm, external pilot randomised controlled trial with an embedded QRI and economic scoping for resource use. Women who require a mastectomy for either breast cancer or risk-reduction, elect to have an IBBR and are considered suitable for both pre and subpectoral reconstruction will be recruited and randomised 1:1 between the techniques.

The QRI will be implemented in two phases: Phase 1, in which sources of recruitment difficulties are rapidly investigated to inform the delivery in Phase 2 of tailored interventions to optimise recruitment of patients.

For peer review only - http://bmjopen.bmj.com/site/about/guidelines.xhtml

UOB Confidential

For peer review only

Primary outcomes will be i) recruitment of patients, ii) adherence to trial allocation and iii) outcome completion rates. Outcomes will be reviewed at 12 months to determine the feasibility of a definitive trial.

Ethics and dissemination

The study has been approved by the NHS Wales REC 6 (20/WA/0338). Findings will be presented at conferences and in peer-reviewed journals.

Trial registration

ISRCTN10081873.

Word Count: 289/300

Strengths and Limitations of this study

- This external pilot RCT with an embedded QuinteT Recruitment Intervention will determine whether it is possible to recruit and randomise patients to a pragmatic trial comparing pre and subpectoral approaches for implant-based breast reconstruction.
- The QuinteT Recruitment Intervention will allow recruitment challenges to be identified, understood, and addressed in real-time, allowing a rapid decision about the feasibility of a definitive trial.
- The external pilot RCT will only address the feasibility of recruitment within an implant reconstruction study; a full-scale main study will still be necessary to compare the clinical and cost-effectiveness of pre and subpectoral implant-based breast reconstruction.

For peer review only - http://bmjopen.bmj.com/site/about/guidelines.xhtml

UOB Confidential

For peer review only

Introduction

55,000 women per year in the UK are diagnosed with breast cancer (1) of whom 40% will require a mastectomy (2). The loss of a breast may dramatically impact women's quality of life (3) and in the UK, the National Institute for Health and Care Excellence (NICE) recommends offering breast reconstruction to improve outcomes (4).

Implant-based breast reconstruction (IBBR) is the most commonly performed breast reconstruction procedure in the UK accounting for almost 70% of all immediate reconstructions performed following mastectomy (5). The introduction of biological or synthetic mesh over the last 10 years has had a major impact on the practice of IBBR. Initially, mesh was sutured between the lower border of the pectoralis muscle and the chest wall extending the subpectoral pocket (6). This allowed a definitive fixed-volume implant to be placed under the muscle at the time of surgery, avoiding the need for tissue expansion and a second procedure and resulting in better cosmetic outcomes through improved lower pole projection (7-14). There are, however, limited data to support the safety or effectiveness of mesh-assisted subpectoral techniques (15-19). The lack of high-quality evidence to support practice is highlighted in the recently updated UK mesh-assisted breast reconstruction guidelines (20) and in March 2021, the United States Food and Drug Administration (FDA) issued a Safety Communication stating that acellular dermal matrices, the most commonly used form of biological mesh, are not licenced for use in breast reconstruction and recommending careful discussion of the risks and benefits of mesh with women considering surgery (21). Despite this mesh-assisted procedures have become established as the standard of care in the UK, with two-thirds of the 2,108 patients undergoing IBBR between 2014 and 2016 in the UK iBRA multicentre prospective cohort study having mesh (18). More recently, pre-pectoral techniques have been introduced in which the implant, fully or partially wrapped in mesh, is placed on top of rather than under the pectoralis major muscle (22). These 'muscle-sparing' techniques may be less painful and avoid the potentially distressing implant 'animation' seen when the pectoralis muscle contracts (23).

For peer review only

For peer review only - http://bmjopen.bmj.com/site/about/guidelines.xhtml

UOB Confidential

Due to the perceived advantages of the procedure, pre-pectoral reconstruction is being widely adopted into practice without high-quality evidence to support its use. Early results are promising (24-31), but caution is required as subcutaneous implant reconstruction without mesh was previously abandoned by the reconstructive community due to high complication rates (32-35). Few studies have directly compared the outcomes of pre and subpectoral mesh-assisted techniques (18, 36-40). These are small, often single centre studies with limited follow up, but have demonstrated no differences in short-term clinical (36-39) or patient-reported (37) outcomes between pre and subpectoral implant placement. The iBRA prospective multicentre cohort study demonstrated that the short-term complications of pre and subpectoral reconstruction were equivalent (18) but suggested that at 18 months, satisfaction with outcome, assessed using the validated BREAST-Q, may be greater in patients receiving pre-pectoral compared with subpectoral techniques (19). Caution is required, however as the numbers of patients undergoing pre-pectoral reconstruction in the iBRA study were small.

High-quality evidence is lacking and there is therefore a need for a well-designed, adequately powered randomised controlled trial (RCT) to evaluate the clinical and cost-effectiveness of IBBR techniques. RCTs in breast reconstruction however are challenging due to patient (41) and surgeon preference (42) and expert opinion suggesting that RCTs in breast reconstruction would be 'unethical', 'impractical' and/or 'inappropriate'(3, 43-46). Previous trials in breast reconstruction have been unsuccessful due to failure to recruit (41, 47). Careful feasibility work to inform the design and conduct of a future study is therefore required before a definitive trial can be planned.

The UK iBRA study (48) used mixed-methods including a national practice questionnaire (49, 50), a randomisation acceptability survey (51) and semi-structured interviews (52) with key stakeholders to explore the most appropriate design for a future RCT. This work suggested that a well-designed trial would be feasible and that key areas of uncertainty included the use of biological vs. synthetic mesh and pre vs. subpectoral implant positioning (51). Pre-pectoral techniques, however, only emerged

For peer review only - http://bmjopen.bmj.com/site/about/guidelines.xhtml

UOB Confidential

For peer review only

during the iBRA study and have gained popularity since with two-thirds of survey respondents now offering the technique. Ongoing work in the Pre-BRA study, an IDEAL 2a/2b study evaluating the safety and stability of pre-pectoral reconstruction prior to definitive evaluation suggests that the technique is safe and sufficiently stable for evaluation in a clinical trial (53).

While the majority of respondents felt that an appropriately designed RCT in IBBR may be possible (51), qualitative interviews with key stakeholders identified a number of barriers to the successful conduct of a future trial (52). These included limited understanding of a pragmatic study design and the role of randomisation in minimising bias, issues around clinician equipoise and aspects of surgical culture not being supportive of RCTs.

Although the iBRA study addressed many of the feasibility issues relating to a future trial including the most appropriate comparators, it did not include a randomised study. It is therefore not clear whether surgeons will recruit patients to an RCT comparing two approaches to IBBR or whether patients will consent to be randomised and agree to receive their allocated treatment. Further feasibility work is therefore needed.

We therefore aimed to undertake an external pilot RCT (Best-BRA study- is subpectoral or pre-pectoral implant placement Best in immediate BReAst reconstruction?). In anticipation of recruitment challenges a QuinteT Recruitment Intervention (QRI) – a complex intervention that aims to rapidly identify, understand, and address sources of recruitment difficulties (54) – has been embedded in the study protocol. Having previously been applied to over 60 RCTs to date, the QRI has led to insights about recruitment issues and the development of targeted strategies that have facilitated successful completion of surgical trials that had previously been considered impossible (54, 55).

For peer review only - http://bmjopen.bmj.com/site/about/guidelines.xhtml

UOB Confidential

The Best-BRA study will determine whether it is possible to recruit and randomise women to a study comparing pre and subpectoral implant-based breast reconstruction and determine the feasibility of progression to a definitive large-scale pragmatic trial.

Methods

This protocol adheres to the Standard Protocol Items for Randomised Trials (SPIRIT) (56) guidelines.

Study Design

The study will consist of a pragmatic multi-centre external pilot RCT with an embedded QRI to determine the feasibility of recruitment and randomisation to either a subpectoral or pre-pectoral implant reconstruction following mastectomy.

Study Setting

NHS breast and plastic surgery units in the UK offering both subpectoral and pre-pectoral immediate implant-based breast reconstruction to women following mastectomy.

Recruitment

Participants will be recruited and randomised over a 12-18 month period by surgeons and research teams working together at participating centres. All female patients undergoing mastectomy who elect to have an IBBR will form the target population. Research teams at each site will maintain a trial screening log following the SEAR framework (57) to determine the proportion of:

i. Patients considered eligible for the study (with reasons for non-eligibility)

ii. Eligible patients approached about the study (with reasons for not being approached)

iii. Eligible patients randomised (with reasons for not being randomised)

For peer review only - http://bmjopen.bmj.com/site/about/guidelines.xhtml

UOB Confidential

iv. Randomised patients who receive their allocated treatment (with reasons for not receiving allocated treatments)

The screening log will be reviewed monthly to provide feedback to recruiters and aid understanding of surgeons' and patients' preferences for types of surgery as part of the QRI.

Potential participants who will have a mastectomy for breast cancer or risk-reduction will be identified from breast cancer and oncoplastic multidisciplinary team meetings (MDTs) (Figure 1). The study will be introduced by the treating surgeon when reconstructive options are discussed, and patients will be given written information outlining the study (Appendix 1). As a key objective of the study is to understand recruitment challenges, these initial consultations will be recorded with patient consent.

Women will provide written consent prior to study participation (Appendix 2). This will include optional consent to be contacted for future studies, and consent to assess long-term patient reported outcomes through linkage to routine data sources.

Eligibility and Allocation

Inclusion criteria

Patients will be included for the trial if they meet all of the following criteria:

1) They are aged 18 years or above.
2) They require a mastectomy for breast cancer or risk-reduction.
3) They elect to undergo immediate IBBR.
4) They are considered eligible for both pre and subpectoral reconstruction by the surgical team.

Exclusion criteria

For peer review only

For peer review only - http://bmjopen.bmj.com/site/about/guidelines.xhtml

UOB Confidential

Patients will be excluded from the trial if they meet any of the following criteria:

1) They have delayed reconstruction

2) They are having revision breast reconstruction surgery

Patients who smoke, have high body mass index (BMI) or have had previous radiotherapy to the breast will not be excluded for participation in the trial but surgeons will be encouraged to offer mesh-assisted IBBR in line with updated joint Association of Breast Surgery (ABS) and British Association of Plastic, Reconstructive and Aesthetic Surgeons (BAPRAS)' guidelines for the use of mesh in reconstructive procedures.

Participants will be randomised by the local research team after eligibility and consent have been confirmed at the final clinic visit by the treating surgeon prior to admission for surgery. The randomisation sequence will be generated by a statistician independent of participant recruitment using the random number generator in Stata statistical software (version 16, StataCorp, College Station, Texas, 2019). Patients will be randomly allocated to the techniques in a 1:1 ratio to either subpectoral or pre-pectoral immediate IBBR stratified by hospital. Women undergoing bilateral surgery will receive the same procedure on both sides. Allocation will be concealed until the patient has been logged into the system and a study ID number generated so ensuring that judgements about eligibility are made without knowledge of the next allocation.

Intervention

All patients will undergo a skin or nipple-preserving, or skin-reducing mastectomy followed by an IBBR. Participating surgeons will undertake the procedure as per their standard practice. Mesh choice (biological or synthetic and the product used) and implant selection (fixed volume; adjustable implants or tissue expanders) will be as per surgeon preference. Two surgeon/two team operating (both procedures performed simultaneously) will be encouraged in bilateral cases to minimise

For peer review only - http://bmjopen.bmj.com/site/about/guidelines.xhtml

UOB Confidential

operative time. The following steps of the IBBR procedure will be considered mandatory, prohibited and flexible/discretionary according to the typology described by Blencowe et al (58).

- **Mandatory** – Insertion of a tissue expander/adjustable implant or fixed volume implant
- **Prohibited** –Raising the pectoralis muscle **IF allocated to pre-pectoral trial arm**
- **Flexible/discretionary** – all other steps of the procedure

Standard Care during and post procedure

Strategies to minimise infection (e.g. use of laminar flow, cavity irrigation, glove change) will be as per local practice but participating centres will be encouraged to adhere to best practice (59) and use the evidence-based Manchester TIC List wherever possible (60). Use of drains and other concomitant interventions will be permitted as per local practice. Post-operative complications will be assessed using internationally agreed standardised definitions (61) by local clinical teams at routine postoperative hospital visits i.e., routine standard of care.

Clinically relevant complications assessed at 3 months and 12 months include:

- Implant loss defined as removal of the expander/implant without replacement
- Infection requiring treatment with antibiotics and/or surgical debridement
- Unplanned re-operation for complications relating to the implant reconstruction
- Readmission to hospital for complications related to the implant reconstruction

Outcome measures

In the definitive trial, the proposed primary outcome will be women's satisfaction with the cosmetic outcome of their reconstruction at 12 months following surgery assessed using the 'Satisfaction with Breasts' domain of the validated BREAST-Q questionnaire (62). On this basis, for the main trial, 100 patients will be required in each group to allow a true 0.5 standard deviation difference in the

For peer review only - http://bmjopen.bmj.com/site/about/guidelines.xhtml

UOB Confidential

BREAST-Q 'Satisfaction with Breasts' domain scale to be detected at 5% significance and 90% power, assuming more than 85% of participants complete the questionnaire 12-months following surgery.

The purpose of this study is to determine the feasibility of conducting a future main trial, specifically whether it will be possible to recruit and randomise 200 patients undergoing IBBR and to collect the required data for the main trial.

The **primary outcomes** for the Best-BRA study are therefore

i) recruitment (number of sites recruiting; proportion of eligible women approached that are randomised, women recruited per site per month)

ii) Adherence to trial allocation

iii) Outcome completion rates at 3 and 12 months.

Secondary outcomes will be the feasibility of collecting the proposed primary and secondary outcomes for the main study. These will include:

i. Satisfaction with breasts using the validated BREAST-Q questionnaire at 12 months. We anticipate that this will be the primary outcome for the definitive trial

ii. Surgical complications, in particular implant loss, infection, re-admission and reoperation within 3 and 12 months of random allocation

iii. The need for additional surgery to the reconstruction or the contralateral breast within 12 months of random allocation

iv. Pain scores assessed using a 10 point Likert scale at 24 hours and 1 week

v. Objective panel assessment of cosmetic outcome at 12 months assessed using routinely collected patient photographs

vi. EQ-5D-5L health related quality of life score (63) and ICECAP-A capability scores (64) at 1 year

For peer review only - http://bmjopen.bmj.com/site/about/guidelines.xhtml
UOB Confidential

vii. Other key patient-reported outcome domains included in the breast reconstruction core outcome set (65) including physical well-being (chest); emotional well-being; and animation assessed using appropriate subscales of the BREAST-Q

viii. The costs of pre-pectoral and subpectoral IBBR procedures

Data collection

Schedule of assessments are summarised in Table 1. All data will be entered directly into electronic case report forms (CRFs) by local research teams and patients will complete electronic patient reported outcome measures (PROMs) on REDCap (66). The feasibility of uploading routinely taken anonymised pre- and post-operative photographs directly onto the REDCap database following specific patient consent will be explored. Reminders will be sent to participants by email/text message for up to 4 weeks if questionnaires at 3 and 12 months have not been completed.

Timepoint	*0 Baseline*	*0 Day of surgery*	*24 hours*	*1 week*	*3 months*	*12$^{+/-10}$ months*
Procedures						
Eligibility criteria review	●					
Informed consent	●					
Randomisation	●					
Sociodemographics	●					
Medical history	●					
Drug history	●					
Collection of routinely taken photographs	●					●
Pain Scores			●	●		
BREAST-Q	●				●	●
Operative Assessments		●				
EQ5D-5L and ICECAP-A	●				●	●
Post-operative histology MDT treatment decisions (e.g. chemotherapy and RT)					●	
Safety						
Adverse events		●			●	●

Table 1 Schedule of assessment visits and outcomes measurements

For peer review only - http://bmjopen.bmj.com/site/about/guidelines.xhtml

UOB Confidential

Participants can choose to discontinue their participation in the trial for any reason. With participant consent, research data obtained up to the point of discontinuation will be retained for analysis. Participants who decide to stop completing patient reported outcome measures will continue to be followed up for complications and adverse event reporting through the review of their medical records unless they specifically object. Participants who are unwilling or unable to go ahead with their allocated treatment will be encouraged to continue in trial follow-up as per protocol and their alternative treatment recorded by the clinical staff team.

Sample size calculation

A formal sample size calculation is not required for this external pilot study. At 12-18 months, the number of sites recruiting; proportion of eligible women that are approached and randomised; the number of women recruited per site per month will be reviewed and adherence to trial allocation will be reviewed. There are no plans to assess the feasibility of recruitment and randomisation in any specific patient subgroups. If the study has opened and is successfully recruiting at an acceptable number of sites with high adherence to treatment allocation and follow up rates, a main trial will be considered feasible and parameters for the full study determined.

Statistical Analysis

Screening, recruitment, adherence to allocated procedure and completeness of primary outcome data will be presented by study group as a CONSORT flow chart, overall and per centre. Secondary outcome measures will be presented as summary statistics at each assessment point. Summary statistics for the two study groups combined will be presented at the end of the pilot phase if the study continues into a definitive trial, with pilot data included in the definitive trial analysis.

For peer review only - http://bmjopen.bmj.com/site/about/guidelines.xhtml
UOB Confidential

Health Economics

The feasibility of applying a novel micro-costing framework to compare the relative costs of pre-pectoral and subpectoral IBBR will be explored (67). A process map will be developed to track the patient pathway from first reconstruction consultation to last routine postoperative visit (usually up to 4 weeks following surgery). The process map will include a list of resources, equipment, consumables, and implants involved in the procedures. Process mapping will be undertaken at several sites to ensure that all appropriate factors and patient pathways are considered. Resources that may potentially differ between the two procedures (e.g. size of mesh; operative time; length of stay and number of postoperative visits) will be identified by reviewing the process maps and details collected on the CRFs. Data may also be gathered from review of electronic notes/medical records. The cost of resources used in the two procedures will be calculated using national tariffs (where available) or local costing estimates.

QuinteT Recruitment Intervention (QRI)

A QRI will be embedded within the Best-BRA study to optimise recruitment and informed consent. The QRI will be implemented in two key phases – Phase 1, in which sources of recruitment difficulties are rapidly investigated to inform the delivery in Phase 2 of tailored interventions to optimise recruitment and informed consent. This will be supplemented with up-front pre-emptive recruitment training in study set up, tailored to anticipated issues and informed by previous QRIs (42, 52, 55, 68, 69).

A multi-faceted, flexible approach will be adopted in Phase 1 using one or more of the following methods:

- Mapping of eligibility and recruitment pathways to collate basic data about the levels of eligibility and recruitment, and identify points at which patients opt in or out of the study;

For peer review only - http://bmjopen.bmj.com/site/about/guidelines.xhtml

UOB Confidential

- In-depth, semi-structured interviews with a purposive sample of staff/site members involved with aspects of study design/management and recruitment across centres. Interviews will explore perspectives on the RCT and issues around recruitment, including views about the study design and protocol, treatment options, existing evidence, and current practice. Interviews may also be undertaken with eligible patients to explore their views on the study, treatment options and provision of information. Interview topic guides will be used to ensure similar topic areas are covered across interviews, while still providing the scope for participants to raise issues of pertinence to them.
- Recording of consultations between healthcare staff and potentially eligible patients in which the study is discussed to explore information provision in relation to key study concepts and treatment options, recruitment techniques, engagement with patient treatment preferences, and randomisation decisions to identify recruitment difficulties and improve information provision. Consultations will be listened to documenting instances such as unclear, insufficient or imbalanced information provision and unintentional transferring of clinician treatment preferences to patients, as well as aspects of good practice.
- Review of study documentation at set up and as recruitment progresses taking account of accumulating interview and consultation data to ensure documents are clear and unbiased.
- Attendance at Trial Management Group (TMG) and investigator meetings to gain an overview of trial conduct and overarching challenges.

Interview and consultation recordings will be transcribed verbatim in full or in parts and edited to ensure anonymity of respondents. Data from Phase 1 will be managed using qualitative data analysis software (such as NVivo). Interviews and recruitment consultations, along with screening logs and study documentation, will be subject to simple counts, content, thematic and targeted conversation analyses to identify aspects of recruitment that are causing difficulties within and across study sites

For peer review only - http://bmjopen.bmj.com/site/about/guidelines.xhtml

UOB Confidential

(for further details see (70). Preliminary analysis will be used to inform strategies for Phase 2 of the QRI and further data collection.

An account of the anonymised findings from all the data will be fed back to the Chief Investigator (CI). The QRI team, with the CI and TMG, will formulate a plan of action grounded in these findings to improve recruitment and information provision, with its format dependent on the nature of recruitment barriers identified (Phase 2). Supportive and responsive group or individual feedback and training is likely to be a core component of the plan of action, including written recruitment 'tips' documents and suggested modifications to study pathways and patient-facing study material.

Phases 1 and 2 will be undertaken in an iterative and cyclical manner, continuing throughout the recruitment period with close monitoring of changes in screening log data and recruiter practice to optimise recruitment and informed consent, all in close collaboration with the CI and wider study team.

Study site/staff and potentially eligible patients will be provided with written information about the recording of consultations and interviews either as a stand-alone document (staff) or as part of the main PIL (patients) and invited to provide written consent. If the patient has yet to receive a PIL, a verbal explanation will be given and verbal consent sought initially at the start of the consultation, with the option to destroy recordings after receipt of PIL. Patient consent to the recordings/interviews is independent of their study participation decision. Data will be captured on encrypted devices and transferred, transcribed and stored in line with university data transfer and storage policies.

Data Management

Data will be entered onto the study REDCap database (66). The system will incorporate data entry and validation rules to reduce data entry errors, and management functions to facilitate auditing and data quality assurance. The Chief Investigator (CI) will have access to and act as custodian of the

For peer review only - http://bmjopen.bmj.com/site/about/guidelines.xhtml

UOB Confidential

full dataset. Participants' data will be held securely held on the in line with data protection legislation. Personal data will not be kept for longer than is required for the purpose for which it has been acquired. Data will be held in compliance with the sponsor's standard operating procedures. Anonymous data sets will be made 'open data' following publication and stored in the University of Bristol's Research Data storage facility.

Monitoring, Safety and Audit

As this is a low-risk external pilot trial, comparing two procedures both of which are in routine clinical practice, the trial is overseen and audited by an independent joint Trial Steering/Data Monitoring Committee (TSC/DMC). The TSC/DMC will comprise of an independent clinician, trials statistician, and PPI member. The TSC/DMC will meet once prior to recruitment of the first participant and convene at least annually to review adverse event data and any other ethical aspects that arise (see Appendix 3 for further details).

Research sites are responsible for reporting serious adverse events (SAEs) for their trial participants during the course of the trial on the electronic CRFs. The following adverse events are expected after surgery in this patient population:

- Anaesthetic complications, e.g. stroke or cardiac events such as myocardial infarction
- Return to theatre or readmission for complications of surgery
- Wound infection requiring treatment with antibiotics, readmission or reoperation
- Postoperative nausea and vomiting
- Pain
- Other infections (sepsis, septicaemia, abscess, respiratory)
- Other procedure specific complication including implant loss, mastectomy skin flap necrosis, infection, seroma
- Allergic or anaphylactic reaction of Patent V blue dye used for sentinel node biopsy

For peer review only - http://bmjopen.bmj.com/site/about/guidelines.xhtml
UOB Confidential

Adverse events will be documented and reported in accordance with the Sponsor's (North Bristol NHS Trust) Safety Reporting Standard Operating Procedure (SOP).

Protocol Amendments

Any amendments to the protocol will be reported to the appropriate regulatory bodies, with a full copy of the current protocol available for download from the study website. There are no amendments to date.

Ancillary and post-trial care

Participants will be treated according to standard care beyond their 12 months in the study.

Public and Patient Involvement (PPI)

PPI members sit on the Trial oversight committees and members of Independent Cancer Patients Voice have provided additional PPI input into all aspects of the study. PPI representatives have been actively involved in all stages of study design including the development of patient information leaflets and planning and refining recruitment strategies. Specifically, they have advised on how and when to approach women undergoing IBBR to minimise the burden of participation and on the selection of outcome measures. PPI involvement will ensure that the patients' perspective is considered and remains the focus of the study. The PPI members will also assist in writing lay summaries and sharing findings of the study with a wider audience. They will advise on methods and content of communication with participants including newsletters and social media.

Please see Appendix 3 for further Administrative Information.

For peer review only - http://bmjopen.bmj.com/site/about/guidelines.xhtml

UOB Confidential

For peer review only

Ethics and dissemination

The NHS Wales Research Ethics Committee 6 (20/WA/0338) reviewed and approved the study. Potential participants will be given at least 24 hours to decide whether they wish to take part and will provide written informed consent (either in person or electronically using the REDCap e-consent function) prior to entering the trial. Specific consent will be obtained for i) upload of pre and postoperative clinical photographs; ii) future contact for long-term follow up with PROMs or via routinely collected data to assess the long-term outcomes and costs of pre and subpectoral implant-based techniques and iii) the collection of anonymised clinical data about their care that will be kept indefinitely after the close of the study as publicly "open data" to support future research studies.

Results of the study will be presented at national and international meetings and published in peer reviewed journals. We will work with our PPI contributors to produce study summaries for patients. If the study is feasible, further funding will be sought for a full-scale trial comparing pre and subpectoral implant-based reconstructive techniques.

Trial Status: Open

Protocol: Version 2.1, 16th March 2021

Open to recruitment: Yes

Planned recruitment duration: 12-18 months

For peer review only

For peer review only - http://bmjopen.bmj.com/site/about/guidelines.xhtml

UOB Confidential

References

1. UK CR. Breast Cancer Statistics [Website]. 2018 [updated Accessed 18th Nov 2018. Available from: http://www.cancerresearchuk.org/health-professional/cancer-statistics/statistics-by-cancer-type/breast-cancer.

2. Malata CM, McIntosh SA, Purushotham AD. Immediate breast reconstruction after mastectomy for cancer. The British journal of surgery. 2000;87(11):1455-72.

3. Harcourt DM, Rumsey NJ, Ambler NR, Cawthorn SJ, Reid CD, Maddox PR, et al. The psychological effect of mastectomy with or without breast reconstruction: a prospective, multicenter study. Plast Reconstr Surg. 2003;111(3):1060-8.

4. NICE. Early and locally advanced breast cancer: diagnosis and management NICE guideline [NG101] 2018 [Available from: https://www.nice.org.uk/guidance/ng101.

5. Mennie JC, Mohanna PN, O'Donoghue JM, Rainsbury R, Cromwell DA. National trends in immediate and delayed post-mastectomy reconstruction procedures in England: A seven-year population-based cohort study. Eur J Surg Oncol. 2017;43(1):52-61.

6. Salzberg CA. Nonexpansive immediate breast reconstruction using human acellular tissue matrix graft (AlloDerm). Annals of Plastic Surgery. 2006;57(1):1-5.

7. Salzberg CA. Nonexpansive immediate breast reconstruction using human acellular tissue matrix graft (AlloDerm). Ann Plast Surg. 2006;57(1):1-5.

8. Gamboa-Bobadilla GM. Implant breast reconstruction using acellular dermal matrix. Ann Plast Surg. 2006;56(1):22-5.

9. Cassileth L, Kohanzadeh S, Amersi F. One-stage immediate breast reconstruction with implants: a new option for immediate reconstruction. Ann Plast Surg. 2012;69(2):134-8.

10. Breuing KH, Colwell AS. Inferolateral AlloDerm hammock for implant coverage in breast reconstruction. Ann Plast Surg. 2007;59(3):250-5.

11. Breuing KH, Warren SM. Immediate bilateral breast reconstruction with implants and inferolateral AlloDerm slings. Ann Plast Surg. 2005;55(3):232-9.

12. Zienowicz RJ, Karacaoglu E. Implant-based breast reconstruction with allograft. Plast Reconstr Surg. 2007;120(2):373-81.

13. Namnoum JD. Expander/implant reconstruction with AlloDerm: recent experience. Plast Reconstr Surg. 2009;124(2):387-94.

14. Ashikari RH, Ashikari AY, Kelemen PR, Salzberg CA. Subcutaneous mastectomy and immediate reconstruction for prevention of breast cancer for high-risk patients. Breast Cancer. 2008;15(3):185-91.

15. Dikmans REG, Negenborn VL, Bouman M-B, Winters HAH, Twisk JWR, Ruhé PQ, et al. Two-stage implant-based breast reconstruction compared with immediate one-stage implant-based breast reconstruction augmented with an acellular dermal matrix: an open-label, phase 4, multicentre, randomised, controlled trial. The Lancet Oncology. 2017;18(2):251-8.

16. Lohmander F, Lagergren J, Johansson H, Roy PG, Frisell J, Brandberg Y. Quality of life and patient satisfaction after implant-based breast reconstruction with or without acellular dermal matrix: randomized clinical trial. BJS Open. 2020;4(5):811-20.

For peer review only - http://bmjopen.bmj.com/site/about/guidelines.xhtml

UOB Confidential

17. Lohmander F, Lagergren J, Roy PG, Johansson H, Brandberg Y, Eriksen C, et al. Implant Based Breast Reconstruction With Acellular Dermal Matrix: Safety Data From an Open-label, Multicenter, Randomized, Controlled Trial in the Setting of Breast Cancer Treatment. Ann Surg. 2019;269(5):836-41.

18. Potter S, Conroy EJ, Cutress RI, Williamson PR, Whisker L, Thrush S, et al. Short-term safety outcomes of mastectomy and immediate implant-based breast reconstruction with and without mesh (iBRA): a multicentre, prospective cohort study. Lancet Oncol. 2019;20(2):254-66.

19. Sewart E, Turner NL, Conroy EJ, Cutress RI, Skillman J, Whisker L, et al. Patient-reported outcomes of immediate implant-based breast reconstruction with and without biological or synthetic mesh. BJS Open. 2021;5(1).

20. Whisker L, Barber M, Egbeare D, Gandhi A, Gilmour A, Harvey J, et al. Biological and synthetic mesh assisted breast reconstruction procedures: Joint guidelines from the Association of Breast Surgery and the British Association of Plastic, Reconstructive and Aesthetic Surgeons. Eur J Surg Oncol. 2021.

21. FDA. Acellular Dermal Matrix (ADM) Products Used in Implant-Based Breast Reconstruction Differ in Complication Rates: FDA Safety Communication 2021 [Available from: https://www.fda.gov/medical-devices/safety-communications/acellular-dermal-matrix-adm-products-used-implant-based-breast-reconstruction-differ-complication.

22. Tasoulis MK, Iqbal FM, Cawthorn S, MacNeill F, Vidya R. Subcutaneous implant breast reconstruction: Time to reconsider? European Journal of Surgical Oncology (EJSO). 2017;43(9):1636-46.

23. Cattelani L, Polotto S, Arcuri MF, Pedrazzi G, Linguadoca C, Bonati E. One-Step Prepectoral Breast Reconstruction With Dermal Matrix-Covered Implant Compared to Submuscular Implantation: Functional and Cost Evaluation. Clin Breast Cancer. 2018;18(4):e703-e11.

24. Berna G, Cawthorn SJ, Papaccio G, Balestrieri N. Evaluation of a novel breast reconstruction technique using the Braxon((R)) acellular dermal matrix: a new muscle-sparing breast reconstruction. ANZ J Surg. 2017;87(6):493-8.

25. Reitsamer R, Peintinger F. Prepectoral implant placement and complete coverage with porcine acellular dermal matrix: a new technique for direct-to-implant breast reconstruction after nipple-sparing mastectomy. J Plast Reconstr Aesthet Surg. 2015;68(2):162-7.

26. Casella D, Bernini M, Bencini L, Roselli J, Lacaria MT, Martellucci J, et al. TiLoop(R) Bra mesh used for immediate breast reconstruction: comparison of retropectoral and subcutaneous implant placement in a prospective single-institution series. Eur J Plast Surg. 2014;37(11):599-604.

27. Casella D, Calabrese C, Bianchi S, Meattini I, Bernini M. Subcutaneous Tissue Expander Placement with Synthetic Titanium-Coated Mesh in Breast Reconstruction: Long-term Results. Plast Reconstr Surg Glob Open. 2015;3(12):e577.

28. Bernini M, Calabrese C, Cecconi L, Santi C, Gjondedaj U, Roselli J, et al. Subcutaneous Direct-to-Implant Breast Reconstruction: Surgical, Functional, and Aesthetic Results after Long-Term Follow-Up. Plast Reconstr Surg Glob Open. 2015;3(12):e574.

For peer review only - http://bmjopen.bmj.com/site/about/guidelines.xhtml
UOB Confidential

29. Becker H, Lind JG, 2nd, Hopkins EG. Immediate Implant-based Prepectoral Breast Reconstruction Using a Vertical Incision. Plast Reconstr Surg Glob Open. 2015;3(6):e412.

30. Vidya R, Masia J, Cawthorn S, Berna G, Bozza F, Gardetto A, et al. Evaluation of the effectiveness of the prepectoral breast reconstruction with Braxon dermal matrix: First multicenter European report on 100 cases. Breast J. 2017;23(6):670-6.

31. Chandarana M, Harries S, Group NBAS. Multicentre study of prepectoral breast reconstruction using acellular dermal matrix. BJS Open. 2020;4:71-7.

32. Snyderman RK, Guthrie RH. Reconstruction of the female breast following radical mastectomy. Plast Reconstr Surg. 1971;47(6):565-7.

33. Gruber RP, Kahn RA, Lash H, Maser MR, Apfelberg DB, Laub DR. Breast reconstruction following mastectomy: a comparison of submuscular and subcutaneous techniques. Plast Reconstr Surg. 1981;67(3):312-7.

34. Rebowe RE, Allred LJ, Nahabedian MY. The Evolution from Subcutaneous to Prepectoral Prosthetic Breast Reconstruction. Plast Reconstr Surg Glob Open. 2018;6(6):e1797.

35. Wagner RD, Braun TL, Zhu H, Winocour S. A systematic review of complications in prepectoral breast reconstruction. J Plast Reconstr Aesthet Surg. 2019;72(7):1051-9.

36. Bettinger LN, Waters LM, Reese SW, Kutner SE, Jacobs DI. Comparative Study of Prepectoral and Subpectoral Expander-Based Breast Reconstruction and Clavien IIIb Score Outcomes. Plastic and reconstructive surgery Global open. 2017;5(7):e1433-e.

37. Baker BG, Irri R, MacCallum V, Chattopadhyay R, Murphy J, Harvey JR. A Prospective Comparison of Short-Term Outcomes of Subpectoral and Prepectoral Strattice-Based Immediate Breast Reconstruction. Plast Reconstr Surg. 2018;141(5):1077-84.

38. Chandarana MN, Jafferbhoy S, Marla S, Soumian S, Narayanan S. Acellular dermal matrix in implant-based immediate breast reconstructions: a comparison of prepectoral and subpectoral approach. Gland surgery. 2018;7(Suppl 1):S64-S9.

39. Thangarajah F, Treeter T, Krug B, Hellmich M, Eichler C, Hanstein B, et al. Comparison of Subpectoral versus Prepectoral Immediate Implant Reconstruction after Skin- and Nipple-Sparing Mastectomy in Breast Cancer Patients: A Retrospective Hospital-Based Cohort Study. Breast Care (Basel). 2019;14(6):382-7.

40. Manrique OJ, Banuelos J, Abu-Ghname A, Nguyen M-D, Tran NV, Martinez-Jorge J, et al. Surgical Outcomes of Prepectoral Versus Subpectoral Implant-based Breast Reconstruction in Young Women. Plastic and reconstructive surgery Global open. 2019;7(3):e2119-e.

41. Winters ZE, Emson M, Griffin C, Mills J, Hopwood P, Bidad N, et al. Learning from the QUEST multicentre feasibility randomization trials in breast reconstruction after mastectomy. The British journal of surgery. 2015;102(1):45-56.

42. Potter S, Mills N, Cawthorn SJ, Donovan J, Blazeby JM. Time to be BRAVE: is educating surgeons the key to unlocking the potential of randomised clinical trials in surgery? A qualitative study. Trials. 2014;15:80.

For peer review only - http://bmjopen.bmj.com/site/about/guidelines.xhtml

UOB Confidential

43. Mullan MH, Wilkins EG, Goldfarb S, Lowery JC, Smith DM, Wickman M, et al. Prospective analysis of psychosocial outcomes after breast reconstruction: cross-cultural comparisons of 1-year postoperative results. J Plast Reconstr Aesthet Surg. 2007;60(5):503-8.

44. Atisha D, Alderman AK, Lowery JC, Kuhn LE, Davis J, Wilkins EG. Prospective analysis of long-term psychosocial outcomes in breast reconstruction: two-year postoperative results from the Michigan Breast Reconstruction Outcomes Study. Ann Surg. 2008;247(6):1019-28.

45. Alderman AK, Wilkins EG, Kim HM, Lowery JC. Complications in postmastectomy breast reconstruction: two-year results of the Michigan Breast Reconstruction Outcome Study. Plast Reconstr Surg. 2002;109(7):2265-74.

46. Fogarty BJ, Brown AP, Miller R, Khan K. TRAM flap versus nonautologous breast reconstruction: what do patients really think? Plast Reconstr Surg. 2004;113(4):1146-52.

47. McCarthy CM, Lee CN, Halvorson EG, Riedel E, Pusic AL, Mehrara BJ, et al. The use of acellular dermal matrices in two-stage expander/implant reconstruction: a multicenter, blinded, randomized controlled trial. Plast Reconstr Surg. 2012;130(5 Suppl 2):57S-66S.

48. Potter S, Conroy E, Williamson P, Thrush S, Whisker LJ, Skillman JM, et al. The iBRA (implant Breast Reconstruction evAluation) Study: Protocol for a prospective multicentre cohort study to inform the feasibility, design and conduct of a pragmatic randomised clinical trial comparing new techniques of implant-based breast reconstruction. . Pilot and Feasibility Studies. 2016;in press.

49. Mylvaganam S, Conroy EJ, Williamson PR, Barnes NLP, Cutress RI, Gardiner MD, et al. Adherence to best practice consensus guidelines for implant-based breast reconstruction: Results from the iBRA national practice questionnaire survey. Eur J Surg Oncol. 2018;44(5):708-16.

50. Mylvaganam S, Conroy E, Williamson PR, Barnes NLP, Cutress RI, Gardiner MD, et al. Variation in the provision and practice of implant-based breast reconstruction in the UK: Results from the iBRA national practice questionnaire. Breast. 2017;35:182-90.

51. The i BRASG. P106. Appropriately-designed randomised trials may be an acceptable method for addressing uncertainties in implant-based breast reconstruction: Preliminary findings from the iBRA Randomisation Acceptability Survey. European Journal of Surgical Oncology. 2019;45(5):912.

52. Davies G, Mills N, Holcombe C, Potter S, i BRASG. Perceived barriers to randomised controlled trials in breast reconstruction: obstacle to trial initiation or opportunity to resolve? A qualitative study. Trials. 2020;21(1):316.

53. Harvey KL, Mills N, White P, Holcombe C, Potter S. The Pre-BRA (pre-pectoral Breast Reconstruction EvAluation) feasibility study: protocol for a mixed-methods IDEAL 2a/2b prospective cohort study to determine the safety and effectiveness of prepectoral implant-based breast reconstruction. 2020;10(1):e033641.

54. Donovan JL, Rooshenas L, Jepson M, Elliott D, Wade J, Avery K, et al. Optimising recruitment and informed consent in randomised controlled trials: the development and implementation of the Quintet Recruitment Intervention (QRI). Trials. 2016;17(1):283.

For peer review only - http://bmjopen.bmj.com/site/about/guidelines.xhtml
UOB Confidential

55. Rooshenas L, Scott LJ, Blazeby JM, Rogers CA, Tilling KM, Husbands S, et al. The QuinteT Recruitment Intervention supported five randomized trials to recruit to target: a mixed-methods evaluation. Journal of clinical epidemiology. 2019;106:108-20.

56. Chan AW, Tetzlaff JM, Gotzsche PC, Altman DG, Mann H, Berlin JA, et al. SPIRIT 2013 explanation and elaboration: guidance for protocols of clinical trials. Bmj. 2013;346:e7586.

57. Wilson C, Rooshenas L, Paramasivan S, Elliott D, Jepson M, Strong S, et al. Development of a framework to improve the process of recruitment to randomised controlled trials (RCTs): the SEAR (Screened, Eligible, Approached, Randomised) framework. Trials. 2018;19(1):50.

58. Blencowe NS, Mills N, Cook JA, Donovan JL, Rogers CA, Whiting P, et al. Standardizing and monitoring the delivery of surgical interventions in randomized clinical trials. British Journal of Surgery. 2016;103(10):1377-84.

59. Knight HJ, Musgrove JJ, Youssef MMG, Ferguson DJ, Olsen SB, Tillett RL. Significantly reducing implant loss rates in immediate implant-based breast reconstruction: A protocol and completed audit of quality assurance. J Plast Reconstr Aesthet Surg. 2020;73(6):1043-9.

60. Barr SP, Topps AR, Barnes NLP, Henderson J, Hignett S, Teasdale RL, et al. Infection prevention in breast implant surgery - A review of the surgical evidence, guidelines and a checklist. European Journal of Surgical Oncology. 2016;42:591-603.

61. Potter S, Davies C, Holcombe C, Weiler-Mithoff E, Skillman J, Vidya R, et al. International development and implementation of a core measurement set for research and audit studies in implant-based breast reconstruction: a study protocol. 2020;10(1):e035505.

62. Pusic A, Klassen A, Scott A, Klok J, ordeiro P G, Cano S. Development of a new patient-reported outcome measure for breast surgery: the BREAST-Q. Plastic and Reconstructive Surgery. 2009;124(2):345-53.

63. Herdman M, Gudex C, Lloyd A, Janssen M, Kind P, Parkin D, et al. Development and preliminary testing of the new five-level version of EQ-5D (EQ-5D-5L). Quality of life research : an international journal of quality of life aspects of treatment, care and rehabilitation. 2011;20(10):1727-36.

64. Al-Janabi H, Flynn TN, Coast J. Development of a self-report measure of capability wellbeing for adults: the ICECAP-A. Quality of life research : an international journal of quality of life aspects of treatment, care and rehabilitation. 2012;21(1):167-76.

65. Potter S, Holcombe C, Ward JA, Blazeby JM. Development of a core outcome set for research and audit studies in reconstructive breast surgery. Br J Surg. 2015;102(11):1360-71.

66. Harris PA, Taylor R, Thielke R, Payne J, Gonzalez N, Conde JG. Research electronic data capture (REDCap)--a metadata-driven methodology and workflow process for providing translational research informatics support. Journal of biomedical informatics. 2009;42(2):377-81.

67. Potter S, Davies C, Davies G, Rice C, Hollingworth W. The use of micro-costing in economic analyses of surgical interventions: a systematic review. Health Econ Rev. 2020;10(1):3.

For peer review only - http://bmjopen.bmj.com/site/about/guidelines.xhtml

UOB Confidential

68. Rooshenas L, Elliott D, Wade J, Jepson M, Paramasivan S, Strong S, et al. Conveying Equipoise during Recruitment for Clinical Trials: Qualitative Synthesis of Clinicians' Practices across Six Randomised Controlled Trials. PLoS Med. 2016;13(10):e1002147.

69. Donovan JL, de Salis I, Toerien M, Paramasivan S, Hamdy FC, Blazeby JM. The intellectual challenges and emotional consequences of equipoise contributed to the fragility of recruitment in six randomized controlled trials. J Clin Epidemiol. 2014;67(8):912-20.

70. Rooshenas L, Paramasivan S, Jepson M, Donovan JL. Intensive Triangulation of Qualitative Research and Quantitative Data to Improve Recruitment to Randomized Trials: The QuinteT Approach. Qualitative health research. 2019;29(5):672-9.

For peer review only

For peer review only - http://bmjopen.bmj.com/site/about/guidelines.xhtml

UOB Confidential

Statements

Author Statement

SP conceived the study and obtained funding; KR, NM, CM, CC, AL, WH, JT, CH, JS, KF, LW, RIC, ST, SC, PF and SP contributed to the study design; CM provided statistical support; NM and CC led the QRI; KR is the Trial Manager responsible for the day-to-day running of the trial. KR prepared the first draft of this manuscript. All authors have read and approved the final manuscript.

Funding

This work is funded by a National Institute for Health Research Clinician Scientist award to SP (CS-2016-16-019). The views expressed in this publication are those of the authors and not necessarily those of the NHS, the National Institute for Health Research or the Department of Health and Social Care.

Acknowledgements

This study is being undertaken with the support of the NIHR Biomedical Research Centre at University Hospitals Bristol and Weston NHS Foundation Trust and the University of Bristol. This study was designed and delivered in collaboration with the Bristol Trials Centre (BTC), a UKCRC Registered Clinical Trials Unit in receipt of National Institute for Health Research CTU support funding.

Trial steering and data monitoring committee members: Matthew Gardiner (chair), Dr Isabelle Smith (statistician) and Elizabeth Teasdale (PPI member).

Declaration of Interests

None

Word Count

4220/4000

For peer review only

For peer review only - http://bmjopen.bmj.com/site/about/guidelines.xhtml

UOB Confidential

For peer review only - http://bmjopen.bmj.com/site/about/guidelines.xhtml
UOB Confidential

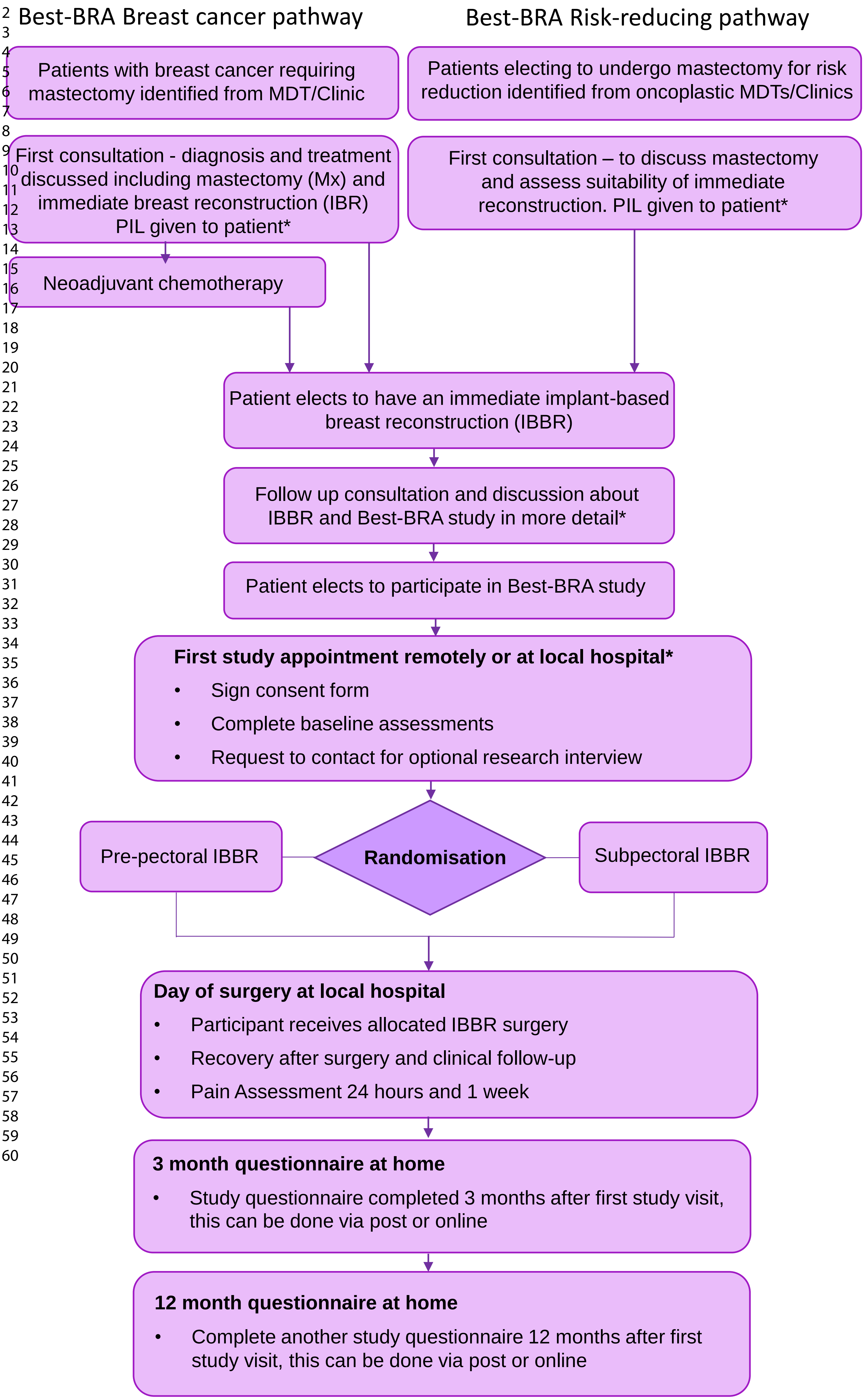

For peer review only - http://bmjopen.bmj.com/site/about/guidelines.xhtml

* These consultations will be recorded with participants' and health care professionals' consent

Best-BRA Study

(Is subpectoral or pre-pectoral implant placement **Best** in immediate **BR**e**A**st reconstruction?)

Patient Information Leaflet

We are inviting you to take part in a research study

- We would like to invite you to take part in a research study. Before you decide to take part, we need you to understand why the research is being done and what it will involve for you.
- Please take time to read the following information carefully and talk to others if you wish.
- Please ask questions using the contact details if anything you read is not clear or you would like further information.

Important things that you need to know

- We are inviting women who are having a mastectomy with an immediate implant-based breast reconstruction to take part in a research study called the Best-BRA study.
- We want to find out whether placing the implant underneath the chest muscle (subpectoral) or above the muscle (pre-pectoral) is best for women who are suitable for either procedure.
- Both procedures are standard practice commonly performed across the UK; neither are new or experimental.
- In the Best-BRA Study, we want to initially find out if such a study is possible before we then do a much larger definitive study.
- Women suitable for both procedures who take part will be allocated to one of two groups: subpectoral or pre-pectoral implant-based breast reconstruction
- We will look at women's satisfaction with their implant-based reconstruction by asking them to complete three study questionnaires for up to 12 months.
- Women who take part can leave the study at any time and will have no impact on the care they receive.
- In this research study we will use information from you and your medical records. We will only use information that we need for the research study.
- We will let very few people know your name or contact details, and only if they really need it for this study.

For peer review only - http://bmjopen.bmj.com/site/about/guidelines.xhtml

- Everyone involved in this study will keep your data safe and secure. We will also follow all privacy rules.
- At the end of the study we will save some of the clinical data in case we need to check it and for future research.
- We will make sure no-one can work out who you are from the reports we write.
- ***If you would like more information on what is involved, please turn over and read Section A***

Contact details:
Name: <INSERT Name of Local Research Nurse>,
Address: <Insert Local hospital address>
Tel: <INSERT Local hospital phone number>
Email: bestbra-study@bristol.ac.uk

For peer review only

For peer review only - http://bmjopen.bmj.com/site/about/guidelines.xhtml

PART A: Why is this study being done and what will happen if you take part?

What is the purpose of the study?

Breast cancer affects one in eight women in the UK and 40% of all women with breast cancer have a mastectomy (removal of the breast) as their surgical treatment. Losing a breast may affect women's self-esteem and body image and breast reconstruction is offered to improve their quality of life.

Breast reconstruction using implants is the most commonly performed procedure in the UK. There are two ways in which implants can be placed during implant-based breast reconstruction; the implants can be placed under the chest muscle (a subpectoral implant) or placed on top of the chest muscle (pre-pectoral implant). Both are standard procedures commonly performed across the UK.

In the Best-BRA study, we want to find out if surgeons and women would be willing to take part in a study to find out if putting the implant on top of the muscle or underneath it improves women's satisfaction with the outcome of their reconstruction. There is currently no good evidence which implant procedure is best for patients (see below).

**Subpectoral reconstruction**	**Pre-pectoral reconstruction**
Implant placed under the chest (pectoral) muscle, implant may or may not be partially covered in mesh	Implant placed on top of the chest (pectoral) muscle, implant usually covered in mesh
Will involve 1-2 nights in hospital	May involve 1 night in hospital or you may be allowed home on the same day
Your surgeon is likely to place 1 or more drains at the time of surgery	Your surgeon is likely to place 1 or more drains at the time of surgery
The implant may move when the chest muscle contracts (this is called animation)	As the implant is on top of the muscle it does not move as much when the chest wall muscle contracts
The muscle covers the implant so the edge of the implant and any ripples on the surface are less easy to see.	The edges of the implant and ripples on the implant surface may be easier to see
May require further surgery to improve the appearance of the reconstruction over time	May be require further surgery to the reconstruction (such as fat transfer) to hide the implant over time

We also want to capture how information about the study is conveyed to patients. This will help us understand how research studies are explained to people and if there are any improvements we can make.

Why have I been invited?

Your surgeon is working with the research team to understand more about which implant-based technique is best. You have been asked to take part in this study because you are having a mastectomy and are suitable for an immediate implant-based breast reconstruction.

We hope to recruit women having immediate implant-based breast reconstruction from UK hospitals.

Do I have to take part?

No. It is your choice whether or not to take part in the Best-BRA study. If you decide to not take part, the treatment and care you receive from your doctors will not be affected in any way. If you do decide to take part, you are also free to leave the study at any time you wish, without giving a reason.

What is involved in the study?

If you take part in the Best-BRA study, following your mastectomy, you will have an immediate implant-based breast reconstruction where either the implant will be placed on top of the chest (pre-pectoral) muscle or below it (subpectoral), both of which are standard procedures. As we do not know which is best for women, the type of surgery you will have will be allocated through a process called

For peer review only - http://bmjopen.bmj.com/site/about/guidelines.xhtml

randomisation. This means you will have an equal chance of having a pre-pectoral implant-based breast reconstruction procedure or a subpectoral implant-based breast reconstruction procedure. Randomisation is used as it creates groups of patients that are similar except for whether the implant is placed under or on top of the muscle. This will enable a fair comparison of the different techniques so we can assess at the end of the study which technique is best. Neither you, the surgeons, or the research team can choose which group you go in, as this could result in the groups being unequal and the findings unreliable. It is important that you only agree to take part if you are prepared to accept either of the two implant-based breast reconstruction procedures.

With your consent, we plan to record conversations where the Best-BRA study is discussed with you. We would like to see how the study is explained to you and if we can improve this. This is optional. As part of this, we may also invite some women to have an optional discussion (research 'interview') with a researcher to explore their views on the study and whether they chose to take part or not. This may take up to an hour, it will be over the telephone and will be recorded with consent.

If I decide to take part, what happens next?

The diagram on page 5 illustrates the flow of the study, which we detail here;

A) First discussion about the Best-BRA study

You will have an appointment with your surgeon and breast care nurse to discuss your surgery and the study in more detail. Following this discussion, a member of the local research team will meet with you at one of your routine clinical appointments, or telephone you, to discuss the study in more detail, answer any questions you may have and arrange your first study visit at your local hospital (see below).

With your consent we would like to record this discussion and any future discussions you have about the study, regardless of whether you decide to take part in the main study or not.

B) First study appointment at your local hospital

This appointment will be at your local hospital if it coincides with one of your routine appointments, otherwise it will be carried out over the phone or using video conferencing if you are happy to do this. At this appointment, a member of the local research team will confirm that you are still happy to take part.

You will be asked to:

- **Sign 2 consent forms** confirming that you agree to the recording of discussions (unless agreed earlier) and that you agree to take part in the study. The consent forms can be completed online or over the phone and they can be posted to you if you are not at the hospital;
- **Complete a study questionnaire** about your general health, how you feel about your body, your experiences and your quality of life before the surgery. The questionnaire should take no more than 20 minutes to complete.
- **Provide some additional information** for the purposes of the study, e.g. contact details, GP information, date of birth.
- If you are having **pre and post-operative photographs** taken as part of your care, you will be asked at this appointment if you are happy for the surgeon or breast care nurse to share these with the research team and they will be reviewed as part of the study. This is optional.

You will then be informed whether you will be in the "Pre-pectoral implant group" or the "Subpectoral implant Group".

We will inform your GP of your participation in the study and let them know which group you have been allocated to.

For peer review only - http://bmjopen.bmj.com/site/about/guidelines.xhtml

University of BRISTOL

NHS North Bristol NHS Trust

Your first consultation to discuss treatment options
Study will be introduced briefly*

- Read Patient Information Leaflet
- Ask any questions

A) First discussion about the Best-BRA study if you decide to have implant-based reconstruction*

At routine clinical appointment or via telephone

Research nurse will arrange your first study appointment

B) First study appointment remotely or at your local hospital*

- Discuss study and ask questions
- Sign consent form
- Request to contact for optional research interview
- Complete study questionnaires
- Allocation to study group

**Pre-pectoral** implant-based breast reconstruction	**OR**	**Subpectoral** implant-based breast reconstruction

C) Surgery at your local hospital

- Preparation for surgery by clinical team- this will include a pre-operative assessment clinic
- You will receive your allocated implant-based breast reconstruction surgery
- Recovery after surgery and clinical follow-up
- Pain Assessment 24 hours and 1 week

D) 3 month questionnaire at home

- Complete study questionnaire 3 months after your first study visit, this can be done via post or online

E) 12 month questionnaire at home

- Complete another study questionnaire 12 months after your first study visit, this can be done via post or online

* These consultations with your consent will be audio recorded

For peer review only - http://bmjopen.bmj.com/site/about/guidelines.xhtml

University of BRISTOL

NHS North Bristol NHS Trust

C) The day of your surgery

You will be invited to attend your local hospital to **have your surgery** (a mastectomy followed by either pre-pectoral or subpectoral implant-based breast reconstruction). The clinical team will prepare you for your surgery as they would during normal NHS care. A member of the research team will also record some other information from your hospital notes about your planned surgery.

The surgeons who will carry out your breast reconstruction in this study perform them regularly. You will receive the usual NHS treatment and aftercare at your hospital (e.g. medical tests, procedures, and follow-up appointments). If you have any doubts, concerns or questions about your operation you should talk to your consultant surgeon, before agreeing to the surgery or to take part in the research.

Please note: you may need to attend a pre-operative assessment clinic before your surgery. Your clinical team will advise you on local procedures.

24 hours and 1 week following your surgery we will ask you about any pain you are experiencing following the surgery. This will be a single question asking you to record this using a scale which can be completed online or with help from the research team.

D) Questionnaire to complete at home: 3 months after your first study visit

You will be asked to **complete a study questionnaire** about your general health, how you feel about your body, your experiences and your quality of life after the surgery. The questionnaire should take no longer than 20 minutes to complete and can be done by post (we will provide a pre-paid envelope) or online.

E) Questionnaire to complete at home: 12 months after your first study visit

12 months after your implant-based breast reconstruction, you will be asked to **complete another study questionnaire** about your general health, how you feel about your body, your experiences and your quality of life after the surgery.

What are the possible benefits of taking part?

This study will not benefit you directly but the information that you provide will help to improve the future management of patients who undergo implant breast reconstructive surgery. Also, some people enjoy being part of a research study because of the close contact with research staff and their opportunity to share their opinions and experiences of their condition and treatments.

What are the possible disadvantages and risks of taking part?

There should be no additional risks to routine NHS practice of either implant-based reconstruction procedure, and neither are new or experimental. You will have the same risks as anyone having immediate implant-based reconstruction. Your doctor will explain the risks and benefit of each procedure, and they will provide relevant hospital leaflets. The questionnaires will take approximately 20 minutes of your time.

What happens after the study is over?

After completing your 12 months questionnaire, you will return to standard NHS care. However, we would like to be able to contact you after this, with your permission. We would like to contact you again to a) check on your long-term health, for example by sending you other questionnaires to add information to what we already know about you, or by checking NHS medical records; and b) to ask you if you would like to take part in other relevant studies. You will not have to reply to any questionnaires or take part in other studies unless you want to at that time.

What if I change my mind and I don't want to carry on with the study?

For peer review only

For peer review only - http://bmjopen.bmj.com/site/about/guidelines.xhtml

University of BRISTOL · independent cancer patients' voice · NIHR National Institute for Health Research · BRISTOL TRIALS CENTRE

University of BRISTOL

NHS North Bristol NHS Trust

You are free to withdraw from the study at any point without giving reason. Your medical treatment will not be affected. If you wish to leave the study, please speak to your doctor or research nurse. If you decide you no longer wish to take part, any information we have collected up until the point you leave will be retained and used in the analysis of the trial results.

What about expenses and travel?

We will provide you with prepaid envelopes to return the study questionnaires if you opt to not complete them online or by telephone. We can offer to cover some travel expenses for study specific hospital visit(s).

What should I do now?

After reading this information sheet, we hope that you will be interested in taking part. You can contact the research nurse if you have any questions. If you would like to take part please also read section B of this information sheet.

PART B: Further general information about the study and what will happen to your data if you decide to take part

Will the information I provide be kept confidential?

Yes. We will follow ethical and legal practice and all information about you will be handled in confidence.

Any information that is collected about you during the study will be kept strictly confidential. All information collected for the study at any time will be stored using a 'study identity number' for confidentiality and will be kept secure using passwords on a University of Bristol server. The information will be handled in line with data protection requirements and will only be available to those responsible for maintaining research standards. We will need to use information from you and/or from your medical records for this research project. This information will include your:

- Initials
- NHS number
- Name
- Gender
- Date of birth
- Contact details (for example: postcode, telephone number, email address)

Your data will be stored and used in compliance with the relevant, current data protection laws; Data Protection Act 2018 and General Data Protection Regulation (GDPR). North Bristol NHS Trust is the sponsor for this study based in the United Kingdom. We will be using information from you and/or your medical records in order to undertake this study and North Bristol NHS Trust and the Bristol Trials Centre (BTC, University of Bristol) will act as joint data controllers for this study. This means that we are responsible for looking after your information safely and using it properly. The BTC at the University of Bristol, who manage the study on behalf of North Bristol NHS Trust, will keep identifiable information about you for 5 years after the study has finished, which is considered good practice for clinical trials. After that period, the data will be securely destroyed (except audio-recordings which will be retained for future studies with your consent).

Your rights to access, change or move your information are limited, as we need to manage your information in specific ways in order for the research to be reliable and accurate. If you withdraw from the study, we will keep the information about you that we have already obtained. To safeguard your rights, we will use the minimum personally identifiable information possible.

Other researchers may wish to access anonymised data from this study in the future. If you take part in the Best-BRA study, anonymous data collected in this study may be used in future studies with the necessary approval. This will not include names,

University of BRISTOL independent cancer patients' voice www.independentcancerpatientsvoice.org.uk NIHR National Institute for Health Research BRISTOL TRIALS CENTRE
For peer review only - http://bmjopen.bmj.com/site/about/guidelines.xhtml

addresses or dates of birth, and it will not be possible to identify you.

If you consent to recording of discussions with study staff and/or a research interview, then all recorded data will be handled in the strictest confidence. Data will be captured on an encrypted audio-recorder or using the video conferencing facilities, transferred securely to the Best-BRA study team at the University of Bristol, and held on a password protected computer. If video conferencing is used, only the audio part of the recording will be retained. Files will be labelled with a reference number (as opposed to your name or any personal information). Recordings will be transcribed (i.e. written word for word) by University of Bristol employees or their authorised representatives. Transcripts (i.e. a written account of what was said) will be anonymised so that you cannot be identified. Data will be analysed and used for training, teaching, research and publication purposes in Best-BRA and future studies. Any quotes used will be anonymised and extracts of recordings will be voice-modified so that you cannot be identified.

Anonymised transcripts of recordings may be made available to other researchers outside of the Best-BRA study via controlled access if they secure the necessary approvals. Data from the anonymised transcripts may be used for purposes not related to this study, but it will not be possible to identify you from them.

You may change your mind about having these consultations or the research interview recorded at any time without it affecting your legal rights or medical care, although any recordings collected before your withdrawal may be retained and used anonymously in a way that will not identify you. Agreeing to record your consultations or taking part in a research interview does not commit you to taking part in the Best-BRA research study.

You can find out more about how we use your information at:

- www.hra.nhs.uk/information-about-patients/
- Our leaflet available from www.nbt.nhs.uk/PatientResearchdata
- By asking one of the research team at bestbra-study@bristol.ac.uk.
- By contacting Helen Williamson (Head of Information Governance) at helen.e.williamson@nbt.nhs.uk or by ringing 0117 41 44767.

Your hospital will collect information from you and/or your medical records for this research study in accordance with our instructions.

Your hospital will use your name, NHS number and contact details to contact you about the research study, and make sure that relevant information about the study is recorded for your care, and to oversee the quality of the study. Individuals from North Bristol NHS Trust, the BTC and regulatory organisations may look at your medical and research records to check the accuracy of the research study. Your hospital will pass these details to the BTC along with the information collected from you and/or your medical records. The only people in North Bristol NHS Trust and the BTC who will have access to information that identifies you will be people who need to contact you to audit the data collection process. The people who analyse the information will not be able to identify you and will not be able to find out your name, NHS number or contact details.

Your hospital will keep identifiable information about you from this study for 5 years after the study has finished (this does not include the audio-recordings).

What will happen to the results of the research study?

The results will be published in medical journals, presented at conferences with other healthcare professionals and specialists, reported on open access databases and open platform research registries and made publicly available. A short version will also be available to you. No one will be able to identify you from any of the study reports.

For peer review only - http://bmjopen.bmj.com/site/about/guidelines.xhtml

Who is organising or sponsoring the research?

The study is being funded by the National Institute of Health Research (NIHR) (reference 2016-16-019) The research is being carried out by a group of experienced doctors and researchers at each of the hospitals involved in the study and the University of Bristol. This study is sponsored by North Bristol NHS Trust, and the Bristol Trials Centre are responsible for managing the study. The study is being conducted by Miss Shelley Potter, Consultant Senior Lecturer at the Centre for Surgical Research University of Bristol.

Who has reviewed the study?

This study has been reviewed by the Health Research Authority and NHS Research Ethics Committee <INSERT Name of REC Committee> who have provided approval for this study to be conducted in the NHS.

What if I have any concerns?

If you have a concern regarding your care as a patient, please discuss this with your surgeon or nurse. If you become unable or unwilling to continue taking part in the Best-BRA Study, we would withdraw you from it. Your medical treatment will continue as usual with your hospital team and GP.

In the unlikely event that something does go wrong, and you are harmed during the research study there are no special compensation arrangements. If you are harmed and this is due to someone's negligence, then you may have grounds for a legal action for compensation against North Bristol NHS Trust, but you may have to pay your legal costs. The normal National Health Service complaints mechanisms will still be available to you.

If you have any questions about the study, or any aspect of your treatment or health whilst on the study, please ask to speak to your Best-BRA study research nurse or surgeon <INSERT name, address, email address, telephone numbers including the 24 hour emergency contact number>.

Alternatively, you can contact the Trial Manager (email: bestbra-study@bristol.ac.uk; phone: 0117 9287351).

The Patient Advice and Liaison Service (PALS) /Advice & Complaints Team (ACT) [delete as appropriate] should be contacted for any complaints. Your local PALS/ACT [delete as appropriate] is:

<INSERT local details>

THANK YOU FOR TAKING THE TIME TO READ THIS INFORMATION.
PLEASE KEEP A COPY FOR YOUR RECORDS.

Funding Acknowledgement: *This project is funded by the National Institute for Health Research (NIHR) (project reference CS-2016-16-019). The views expressed are those of the author(s) and not necessarily those of the NIHR or the Department of health and Social Care.*

BTC Acknowledgement: *This study was designed and delivered in collaboration with the Bristol Trials Centre (BTC), a UKCRC registered clinical trials unit which, as part of the Bristol Trials Centre, is in receipt of National Institute for Health Research CTU support funding.*

For peer review only

For peer review only - http://bmjopen.bmj.com/site/about/guidelines.xhtml

University of BRISTOL | FUNDED BY NIHR National Institute for Health Research | BRISTOL TRIALS CENTRE | NHS North Bristol NHS Trust

Best-BRA Study
(Is subpectoral or pre-pectoral implant placement **Best** in immediate **BR**e**A**st reconstruction?)

Participant Consent Form

Participant ID:						

Please initial Relevant boxes

1. I confirm that I have read the patient Information leaflet <INSERT version & date> for the above study. I have had the opportunity to consider the information, ask questions and have had these answered satisfactorily. ☐

2. I understand that my participation is voluntary and that I am free to withdraw at any time without giving any reason, without my medical care or legal rights being affected. I understand that all information collected up until the point I withdraw will be retained for analysis. ☐

3. I understand that after the study ends, the research data (results) collected may be made "open data". This means the data will be publicly available and may be used for purposes not related to this study. However, any personal information that could identify me will be removed or changed before files are shared with other researchers or results are made public. This does not apply to QRI data that is 'controlled access' (see separate consent form regarding the audio-recording of consultations). ☐

4. I understand that relevant sections of my medical notes and data collected during the study, including personal identifiable data may be looked at by individuals from regulatory authorities, the University of Bristol study research team or from the NHS Trust, to ensure that the research is conducted appropriately. I give permission for these individuals to have access to my records. ☐

5. I agree that my GP will be told that I am taking part in this research study. ☐

6. I give consent for the data collected in this trial to be used in future ethically approved studies on the understanding that all information will continue to be kept securely and remain confidential. ☐

Please continue on next page

For peer review only

For peer review only - http://bmjopen.bmj.com/site/about/guidelines.xhtml

7. *OPTIONAL: I agree for my pre-operative and post-operative photographs if they have been taken, to be shared securely with the research team and to be analysed as part of the study* ☐

8. *OPTIONAL: I agree to being contacted in the future by the research team to provide information about my long-term health status. I understand I can then decide to not take part in any future studies if I change my mind.* ☐

7. I agree to take part in the above Best-BRA Study. ☐

______________________________	______________________________	______________________________
**Name of patient**	**Signature**	**Date**
______________________________	______________________________	______________________________
**Name of person taking consent**<br>You must have signed the<br>Site Signature & Delegation Log	**Signature**	**Date**

Original to be kept in the Investigator Site File, 1 copy in hospital notes, 1 copy to the patient, 1 copy to the Trial Office

Funding Acknowledgement: *This project is funded by the National Institute for Health Research (NIHR) HTA programme (project reference 17/95/03). The views expressed are those of the author(s) and not necessarily those of the NIHR or the Department of health and Social Care.*

BRTC Acknowledgement: *This study was designed and delivered in collaboration with the Bristol Randomised Trials Collaboration (BRTC), a UKCRC registered clinical trials unit which, as part of the Bristol Trials Centre, is in receipt of National Institute for Health Research CTU support funding.*

For peer review only - http://bmjopen.bmj.com/site/about/guidelines.xhtml

For peer review only

Appendix 3: Administrative information

Title

Best-BRA (Is subpectoral or pre-pectoral implant placement best in immediate breast reconstruction?): a protocol for a pilot randomised controlled trial of subpectoral versus pre-pectoral immediate implant-based breast reconstruction in women following mastectomy.

Trial registration number

ISRCTN: 15397185 (12 January 2021)

World Health Organization Trial Registration Data Set

Data category	Information
Primary registry and trial identifying number	ISRCTN 10081873
Date of registration in primary registry	12 January 2021
Secondary identifying numbers	IRAS: 279460<br>NHS REC: 20/WA/0338
Source(s) of monetary or material support	National Institute for Health Research (NIHR)
Primary sponsor	North Bristol NHS Trust
Secondary sponsor(s)	Not applicable
Contact for public queries	Dr Kirsty Roberts, bestbra-study@bristol.ac.uk, 07989981816
Contact for scientific queries	Ms Shelley Potter, shelley.potter@bristol.ac.uk, 0117 9287218
Public title	Best-BRA
Scientific title	Is subpectoral or pre-pectoral implant placement best in immediate breast reconstruction? The Best-BRA pilot RCT
Countries of recruitment	England, Wales, Scotland, Northern Ireland
Health condition(s) or problem(s) studied	Breast cancer
Intervention(s)	Subpectoral or pre-pectoral immediate implant-based breast reconstruction following mastectomy
Key inclusion and exclusion criteria	Inclusion: Women aged 18 or over; Require a mastectomy for breast cancer (invasive or preinvasive disease) or risk-reduction; Elect to undergo immediate IBBR; Considered eligible for either pre or subpectoral reconstruction by the surgical team.<br>Exclusion: Delayed reconstruction; Revisional surgery
Study type	Intervention
Date of first enrolment	To be determined

For peer review only

Target sample size	Pilot trial so not applicable
Recruitment status	Open to recruitment
Primary outcome(s)	Recruitment (number of sites recruiting; proportion of eligible women approached that are randomised, women recruited per site per month), adherence to trial allocation and outcomes completion rates will be reviewed at 12 months. At 12 months the parameters required for a fully powered trial will also be assessed (e.g number of sites and sample size).
Key secondary outcomes	Feasibility of collecting the following outcomes:<br>• The difference between groups in satisfaction with breasts using the validated BREAST-Q questionnaire at 12 months<br>• Differences between the groups in pain scores at 24 hours and 1 week<br>• The difference between groups in surgical complications, in particular implant loss, infection, re-admission and reoperation at 3 and 12 months<br>• The difference between groups in the need for additional surgery to the reconstruction or the contralateral breast at 12 months<br>• Differences between the groups in objective cosmetic outcome at 12 months assessed using patient photographs<br>• The difference between groups in their average EQ-5D-5L health related quality of life score and ICECAP-A scores at 1 year<br>• The difference between groups with respect to other key patient reported outcome domains included in the breast reconstruction core outcome set[1] including physical well-being; emotional well-being; and animation assessed using appropriate subscales of the BREAST-Q<br>• The cost effectiveness of pre-pectoral and subpectoral IBBR

For peer review only

Protocol version

Version 2.1 (16 March 2021)

Version		Notes
Number	Date	
2.1	16.03.21	This was approved by REC/HRA

Funding

NIHR

Contributorship

See main manuscript

Sponsor contact information

Trial sponsor: North Bristol NHS Trust
Sponsor's reference: 4716
Contact name: Mr Paolo Buscemi
Address: Research & Innovation, North Bristol NHS Trust, Level 3, Learning & Research building, Southmead Hospital, Westbury on Trym, Bristol, BS10 5NB
Tel: 0117 41 49345
E-mail: researchSponsor@nbt.nhs.uk

Role of study sponsor and funder

The funder and sponsor had no role in the design of this study and will not have any role during its execution, analyses, interpretation of the data, or decision to submit results.

Committees

The Trial Management Group (TMG) comprises all investigators, the trial manager, research and staff, with input from a patient/public representative. Members of the TMG will contribute to the trial in the following ways: trial design and methods; participant recruitment and trial conduct; trial management; trial logistics and cost management; economic evaluation; qualitative (QRI) study; statistical data analysis; and publication. The TMG will meet on a regular basis to oversee the management of the trial. The TMG will be provided with detailed information by the centre staff regarding trial progress. Meetings will be via videoconference facilities.

This study was designed and is being delivered in collaboration with the Bristol Trials Centre (BTC), a UKCRC registered clinical trials unit which is in receipt of National Institute for Health Research CTU support funding. Members of the BTC will attend the TMG.

Because this is a low-risk trial, the funder has agreed that the roles of both guiding the Trial Management Group and monitoring trial data will be undertaken by a single Trial Steering/Data Monitoring Committee (TS/DMC). The TS/DMC will meet at least three times over the course of the study and comprises three independent members: a chairperson, a statistician, and a patient representative. Their role will be to provide overall supervision of the trial on behalf of the funder, with a focus on progress of the trial, adherence to the protocol, patient safety and consideration of new information. The committee will review the accruing data and assess whether there are any safety issues that should be brought to the Sponsor's or the participants' attention or any reasons for the trial not to continue. Terms of reference will be drawn up and agreed with members of the TS/DMC.

For peer review only

STANDARD PROTOCOL ITEMS: RECOMMENDATIONS FOR INTERVENTIONAL TRIALS

SPIRIT 2013 Checklist: Recommended items to address in a clinical trial protocol and related documents*

Section/item	Item No	Description	Location
**Administrative information**			
Title	1	Descriptive title identifying the study design, population, interventions, and, if applicable, trial acronym	Page 1
Trial registration	2a	Trial identifier and registry name. If not yet registered, name of intended registry	Page 4
	2b	All items from the World Health Organization Trial Registration Data Set	Appendix 1
Protocol version	3	Date and version identifier	Appendix 1
Funding	4	Sources and types of financial, material, and other support	Page 25
Roles and responsibilities	5a	Names, affiliations, and roles of protocol contributors	Page 25
	5b	Name and contact information for the trial sponsor	Appendix 1
	5c	Role of study sponsor and funders, if any, in study design; collection, management, analysis, and interpretation of data; writing of the report; and the decision to submit the report for publication, including whether they will have ultimate authority over any of these activities	Page 25/Appendix 1
	5d	Composition, roles, and responsibilities of the coordinating centre, steering committee, endpoint adjudication committee, data management team, and other individuals or groups overseeing the trial, if applicable (see Item 21a for data monitoring committee)	Page 16
**Introduction**			

For peer review only - http://bmjopen.bmj.com/site/about/guidelines.xhtml

Background and rationale	6a	Description of research question and justification for undertaking the trial, including summary of relevant studies (published and unpublished) examining benefits and harms for each intervention	Page 5
	6b	Explanation for choice of comparators	Page 6/7
Objectives	7	Specific objectives or hypotheses	Page 7
Trial design	8	Description of trial design including type of trial (eg, parallel group, crossover, factorial, single group), allocation ratio, and framework (eg, superiority, equivalence, noninferiority, exploratory)	Page 8
**Methods: Participants, interventions, and outcomes**			
Study setting	9	Description of study settings (eg, community clinic, academic hospital) and list of countries where data will be collected. Reference to where list of study sites can be obtained	Page 8
Eligibility criteria	10	Inclusion and exclusion criteria for participants. If applicable, eligibility criteria for study centres and individuals who will perform the interventions (eg, surgeons, psychotherapists)	Page 9,10
Interventions	11a	Interventions for each group with sufficient detail to allow replication, including how and when they will be administered	Pages 9-10
	11b	Criteria for discontinuing or modifying allocated interventions for a given trial participant (eg, drug dose change in response to harms, participant request, or improving/worsening disease)	N/A
	11c	Strategies to improve adherence to intervention protocols, and any procedures for monitoring adherence (eg, drug tablet return, laboratory tests)	Page 16
	11d	Relevant concomitant care and interventions that are permitted or prohibited during the trial	Pages 9-11

For peer review only - http://bmjopen.bmj.com/site/about/guidelines.xhtml

Outcomes	12	Primary, secondary, and other outcomes, including the specific measurement variable (eg, systolic blood pressure), analysis metric (eg, change from baseline, final value, time to event), method of aggregation (eg, median, proportion), and time point for each outcome. Explanation of the clinical relevance of chosen efficacy and harm outcomes is strongly recommended	Pages 11-12, Table 1
Participant timeline	13	Time schedule of enrolment, interventions (including any run-ins and washouts), assessments, and visits for participants. A schematic diagram is highly recommended (see Figure)	Pages 8-10, Figure 1 and Table 1
Sample size	14	Estimated number of participants needed to achieve study objectives and how it was determined, including clinical and statistical assumptions supporting any sample size calculations	Page 11 & 13
Recruitment	15	Strategies for achieving adequate participant enrolment to reach target sample size	Pages 8 & 14-16 (QRI)
**Methods: Assignment of interventions (for controlled trials)**			
Allocation:			
Sequence generation	16a	Method of generating the allocation sequence (eg, computer-generated random numbers), and list of any factors for stratification. To reduce predictability of a random sequence, details of any planned restriction (eg, blocking) should be provided in a separate document that is unavailable to those who enrol participants or assign interventions	Page 10
Allocation concealment mechanism	16b	Mechanism of implementing the allocation sequence (eg, central telephone; sequentially numbered, opaque, sealed envelopes), describing any steps to conceal the sequence until interventions are assigned	Page 10
Implementation	16c	Who will generate the allocation sequence, who will enrol participants, and who will assign participants to interventions	Page 10
Blinding (masking)	17a	Who will be blinded after assignment to interventions (eg, trial participants, care providers, outcome assessors, data analysts), and how	n/a

	17b	If blinded, circumstances under which unblinding is permissible, and procedure for revealing a participant's allocated intervention during the trial	n/a
**Methods: Data collection, management, and analysis**			
Data collection methods	18a	Plans for assessment and collection of outcome, baseline, and other trial data, including any related processes to promote data quality (eg, duplicate measurements, training of assessors) and a description of study instruments (eg, questionnaires, laboratory tests) along with their reliability and validity, if known. Reference to where data collection forms can be found, if not in the protocol	Page 12-13 & 16 and Table 1
	18b	Plans to promote participant retention and complete follow-up, including list of any outcome data to be collected for participants who discontinue or deviate from intervention protocols	Page 13
Data management	19	Plans for data entry, coding, security, and storage, including any related processes to promote data quality (eg, double data entry; range checks for data values). Reference to where details of data management procedures can be found, if not in the protocol	Pages 15
Statistical methods	20a	Statistical methods for analysing primary and secondary outcomes. Reference to where other details of the statistical analysis plan can be found, if not in the protocol	Page 13
	20b	Methods for any additional analyses (eg, subgroup and adjusted analyses)	N/A
	20c	Definition of analysis population relating to protocol non-adherence (eg, as randomised analysis), and any statistical methods to handle missing data (eg, multiple imputation)	N/A
**Methods: Monitoring**			
Data monitoring	21a	Composition of data monitoring committee (DMC); summary of its role and reporting structure; statement of whether it is independent from the sponsor and competing interests; and reference to where further details about its charter can be found, if not in the protocol. Alternatively, an explanation of why a DMC is not needed	Page 16

For peer review only - http://bmjopen.bmj.com/site/about/guidelines.xhtml

	21b	Description of any interim analyses and stopping guidelines, including who will have access to these interim results and make the final decision to terminate the trial	n/a
Harms	22	Plans for collecting, assessing, reporting, and managing solicited and spontaneously reported adverse events and other unintended effects of trial interventions or trial conduct	Pages 16-17
Auditing	23	Frequency and procedures for auditing trial conduct, if any, and whether the process will be independent from investigators and the sponsor	Page 16
**Ethics and dissemination**			
Research ethics approval	24	Plans for seeking research ethics committee/institutional review board (REC/IRB) approval	Page 4 % 18
Protocol amendments	25	Plans for communicating important protocol modifications (eg, changes to eligibility criteria, outcomes, analyses) to relevant parties (eg, investigators, REC/IRBs, trial participants, trial registries, journals, regulators)	Page 17
Consent or assent	26a	Who will obtain informed consent or assent from potential trial participants or authorised surrogates, and how (see Item 32)	Pages 8-9,
	26b	Additional consent provisions for collection and use of participant data and biological specimens in ancillary studies, if applicable	N/A
Confidentiality	27	How personal information about potential and enrolled participants will be collected, shared, and maintained in order to protect confidentiality before, during, and after the trial	Pages 16
Declaration of interests	28	Financial and other competing interests for principal investigators for the overall trial and each study site	Page 25
Access to data	29	Statement of who will have access to the final trial dataset, and disclosure of contractual agreements that limit such access for investigators	Pages 16
Ancillary and post-trial care	30	Provisions, if any, for ancillary and post-trial care, and for compensation to those who suffer harm from trial participation	Page 10- 11

For peer review only - http://bmjopen.bmj.com/site/about/guidelines.xhtml
For peer review only

Dissemination policy	31a	Plans for investigators and sponsor to communicate trial results to participants, healthcare professionals, the public, and other relevant groups (eg, via publication, reporting in results databases, or other data sharing arrangements), including any publication restrictions	Page 18
	31b	Authorship eligibility guidelines and any intended use of professional writers	N/A
	31c	Plans, if any, for granting public access to the full protocol, participant-level dataset, and statistical code	Page 16
**Appendices**			
Informed consent materials	32	Model consent form and other related documentation given to participants and authorised surrogates	Appendix 2 & Appendix 3
Biological specimens	33	Plans for collection, laboratory evaluation, and storage of biological specimens for genetic or molecular analysis in the current trial and for future use in ancillary studies, if applicable	N/A

*It is strongly recommended that this checklist be read in conjunction with the SPIRIT 2013 Explanation & Elaboration for important clarification on the items. Amendments to the protocol should be tracked and dated. The SPIRIT checklist is copyrighted by the SPIRIT Group under the Creative Commons "Attribution-NonCommercial-NoDerivs 3.0 Unported" license.

For peer review only
